# A causal relationship between irritability and cardiovascular diseases: a Mendelian randomization study

**DOI:** 10.3389/fcvm.2023.1174329

**Published:** 2023-05-30

**Authors:** Dihui Cai, Yin Fu, Yongfei Song, Hui Lin, Yanna Ba, Jiangfang Lian

**Affiliations:** Department of Cardiology, Lihuili Hospital Affiliated to Ningbo University, Ningbo University, Ningbo Institute of Innovation for Combined Medicine and Engineering, Ningbo, China

**Keywords:** cardiovascular disease, irritability, coronary artery disease, heart failure, Mendelian randomization

## Abstract

**Background:**

Observational studies have suggested that irritability is associated with a higher risk of cardiovascular disease (CVD). However, the potential causal association is not clear. Therefore, we used Mendelian randomization (MR) analysis to assess the causal association of irritability with CVD risk.

**Methods:**

A two-sample MR analysis was performed to confirm the causal association of irritability with the risk of several common CVDs. The exposure data were derived from the UK biobank involving 90,282 cases and 232,386 controls, and outcome data were collected from the published genome-wide association studies (GWAS) and FinnGen database. Inverse-variance weighted (IVW), MR-Egger, and weighted median methods were performed to assess the causal association. Furthermore, the mediating effect of smoking, insomnia, and depressed affect was explored by using a two-step MR.

**Results:**

The MR analysis indicated that genetically predicted irritability increased the risk of CVD, including coronary artery disease (CAD) (Odds ratio, OR: 2.989; 95% confidence interval, CI: 1.521–5.874, *p* = 0.001), myocardial infarction (MI) (OR: 2.329, 95% CI: 1.145–4.737, *p* = 0.020), coronary angioplasty (OR: 5.989, 95% CI: 1.696–21.153, *p* = 0.005), atrial fibrillation (AF) (OR: 4.646, 95% CI: 1.268–17.026, *p* = 0.02), hypertensive heart disease (HHD) (OR: 8.203; 95% CI: 1.614–41.698, *p* = 0.011), non-ischemic cardiomyopathy (NIC) (OR: 5.186; 95% CI: 1.994–13.487, *p* = 0.001), heart failure (HF) (OR: 2.253; 95% CI: 1.327–3.828, *p* = 0.003), stroke (OR: 2.334; 95% CI: 1.270–4.292, *p* = 0.006), ischemic stroke (IS) (OR: 2.249; 95% CI: 1.156–4.374, *p* = 0.017), and ischemic stroke of large-artery atherosclerosis ISla (OR: 14.326; 95% CI: 2.750–74.540, *p* = 0.002). The analysis also indicated that smoking, insomnia, and depressed affect play an important role in the process of irritability leading to cardiovascular disease.

**Conclusion:**

Our findings support the first genetic evidence of the causality of genetically predicted irritability with the risk of developing into CVDs. Our results deliver a viewpoint that more early active interventions to manage an individual's anger and related unhealthy lifestyle habits are needed to prevent the occurrence of adverse cardiovascular events.

## 1. Introduction

Cardiovascular disease (CVD) is a general term for cardiac and vascular diseases such as coronary artery disease (CAD), hypertension, atrial fibrillation (AF), heart failure (HF), and stroke (1, 2). CVDs are the main cause of morbidity, disability, and mortality worldwide, causing a substantial burden on the economy, society, and healthcare. CVDs caused approximately 19 million deaths globally in 2020, with an increase of 18.7% in 10 years (3). The identification of risk factors is expected to mitigate the occurrence and development of CVDs through early prevention and intervention (4).

Irritability is best described by impulsiveness, anger, and hostility and is a tendency to overreact to aversive stimuli with negative affect (5). Some observational studies have shown that irritability is significantly related to various CVDs. A recent cohort study showed that irritability is independently associated with severe CAD (6). In addition, some prospective studies suggested that the level of anger had a dose–response relationship with CAD (7, 8). Meanwhile, irritability also increased the risk of AF (9), HF (10), hypertension (11), and stroke (12).

Although the increasing evidence indicates that irritability is a risk factor for CVDs, it is unclear whether this association merely stems from irritability. Since most findings are based on observational studies, the role of confounding factors and reverse causality cannot be ignored (13). Clarifying the causality between irritability and CVDs is helpful in better preventing the potential risk of adverse cardiovascular events.

Mendelian randomization (MR) analysis is an epidemiological tool that confirms a causal association between a trait and an outcome by applying genetic variants as proxies (14). Because genetic variants have the excellent feature that it is randomly allocated when passed from parents to offspring, the potential confounders and reverse causation bias of observational studies are effectively reduced and circumvented to reinforce causal inference (15).

Nevertheless, so far, no MR studies have focused on the causal inference between irritability and CVDs. In this study, the objective is to assess the potential causal association between irritability and various CVDs by implementing a two-sample MR analysis and a two-step MR analysis.

## 2. Methods

### 2.1. Study design and data sources

**Figure 1:**
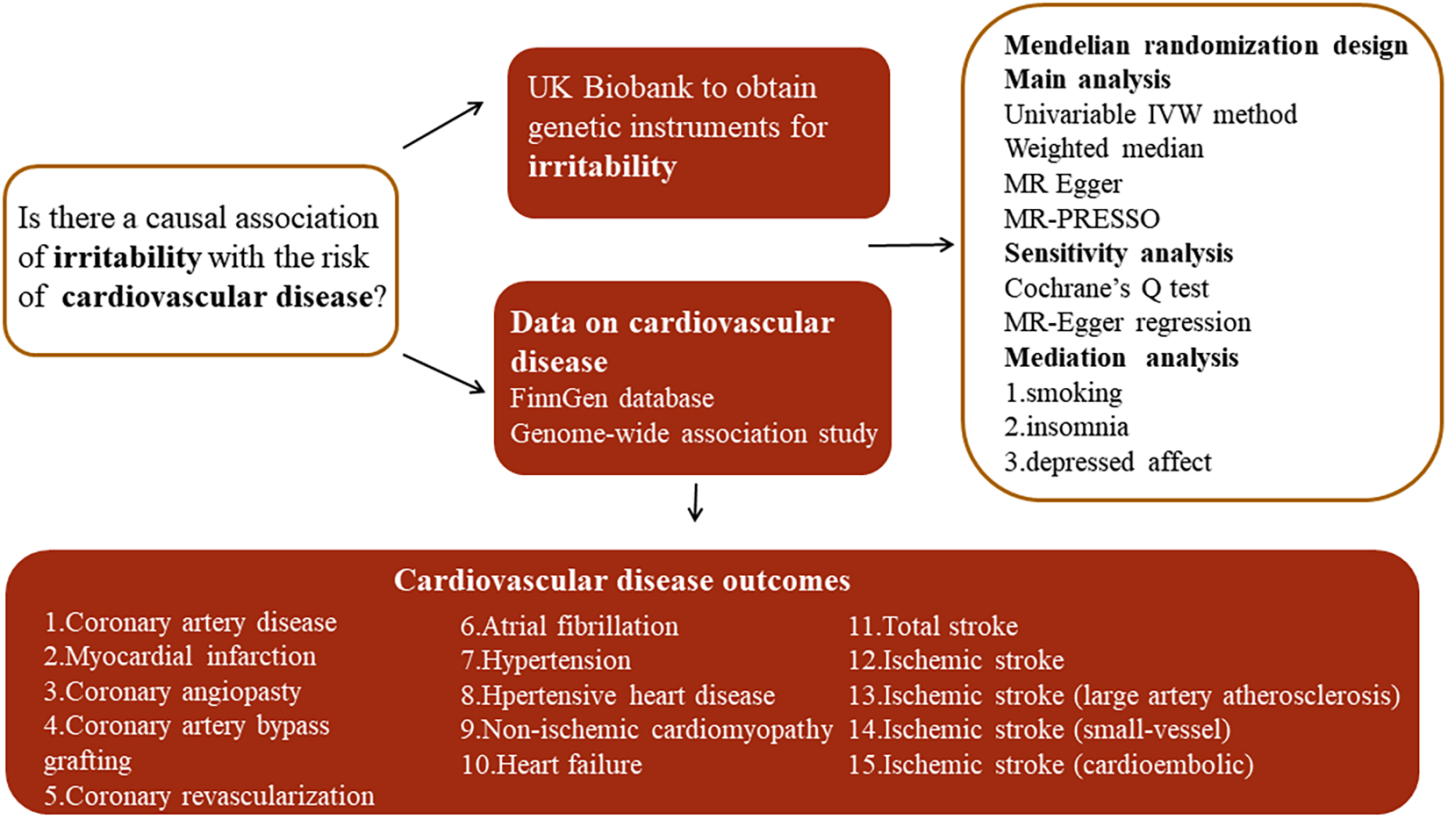
Overview of the study design. MR-PRESSO, MR pleiotropy residual sum and outlier test; IVW, inverse variance weighted.

A summary of the study design is presented in Figure 1. Two-sample MR analyses were performed to evaluate the causality between genetic susceptibility to irritability and the risk of several CVDs. Moreover, we used a two-step MR to estimate whether the causal relationship is mediated by CVD risk factors such as smoking, insomnia, and depressed affect.

Genetic variants (single-nucleotide polymorphisms (SNPs)) were applied as instrumental variables (IVs) to explore the causal relationship. The whole process followed the three main assumptions in MR analysis: (a) IVs are robustly related to exposure factors; (b) IVs are not associated with confounding factors; and (c) IVs influence the risk of the outcomes only via exposure (10). Summary-level data for irritability were obtained from the UK Biobank (UKB) involving 90,282 cases and 232,386 controls. The question “Are you an irritable person?” was used to reflect irritability in the data sources. The data for various CVDs were derived from recently publicly available genome-wide association studies (GWASs) and the FinnGen GWAS database, including coronary artery disease (CAD), myocardial infarction (MI), coronary angioplasty (CA), coronary artery bypass grafting (CABG), coronary revascularization (CR), atrial fibrillation (AF), hypertension (HTN), hypertensive heart disease (HHD), non-ischemic cardiomyopathy (NIC), stroke, ischemic stroke (IS), ischemic stroke of large-artery atherosclerosis (ISla), ischemic stroke of small-vessel (ISsv), and cardioembolic ischemic stroke (ISce). All summary statistics associated with irritability and CVDs in the present study were retrieved from the IEU OpenGWAS Project (https://gwas.mrcieu.ac.uk/), and a detailed description is listed in Table 1.

**Table 1:** Data sources.

Phenotypes	Data source	Phenotypic code	Cases/controls	Ancestry
Exposures				
Irritability	UK Biobank	ukb-a-47	90,282/232,386	European
Outcome				
Coronary artery disease	UK Biobank + CARDIoGRAMplusC4D	ebi-a-GCST005195	122,733/424,528	European
Myocardial infarction	UK Biobank + CARDIoGRAMplusC4D	ebi-a-GCST011365	14,825/44,000	European
Coronary angioplasty	FinnGen	finn-b-I9_ANGIO	7,920/187,840	European
Coronary artery bypass grafting	FinnGen	finn-b-I9_CABG	5,779/187,840	European
Coronary revascularization	FinnGen	finn-b-I9_REVASC	12,271/187,840	European
Heart failure	Shah S. et al.	ebi-a-GCST009541	47,309/930,014	European
Non-ischemic cardiomyopathy	FinnGen	finn-b-I9_NONISCHCARDMYOP	11,400/175,752	European
Atrial fibrillation	FinnGen	finn-b-I9_AF	22,068/116,926	European
Hypertension	FinnGen	finn-b-I9_HYPTENS	55,917/162,837	European
Hypertensive heart disease	FinnGen	finn-b-I9_HYPTENSHD	3,938/162,837	European
Stroke	Malik R. et al.	ebi-a-GCST006906	40,585/406,111	European
Ischemic stroke	Malik R. et al.	ebi-a-GCST006908	34,217/406,111	European
Ischemic stroke (large-artery atherosclerosis)	Malik R. et al.	ebi-a-GCST006907	4,373/406,111	European
Ischemic stroke (small-vessel)	Malik R. et al.	ebi-a-GCST006909	5,386/406,111	European
Ischemic stroke (cardioembolic)	Malik R. et al.	ebi-a-GCST006910	7,193/406,111	European
Mediator				
Smoking initiation	GSCAN	ieu-b-4877	311,629/321,173	European
Insomnia complaints	Hammerschlag AR. et al	ebi-a-GCST004695	12,863/19,521	European
Depressed affect	Nagel M. et al	ebi-a-GCST006475	357,957	European

### 2.2. Selection of instrumental variables

SNPs were identified as IVs at the genome-wide significance threshold (*p* < 5 × 10^−8^). To ensure independence between the included SNPs as IVs, we set the linkage disequilibrium (LD) threshold for grouping to *r*^2^ < 0.001 and a genetic distance of 10,000 kb. PhenoScanner (16) was used to search for each included SNP to estimate the associations with potential confounders (BMI, hypertension, smoking, insomnia, and depressed affect) and exclude those strongly associated with the confounders (*p* < 5 × 10^−8^). The details of the used SNPs between exposure (irritability, smoking, insomnia, and depressed affect) and outcomes (CVDs) are given in Supplementary Table S1–S43. *F*-statistics were used to eliminate the weak instrument bias of the used SNPs. An *F*-statistic >10 indicated the correlation between the IVs, and exposure was considered sufficiently strong and avoided a weak instrument bias (17).

### 2.3. Statistical analyses

R packages (TwoSampleMR and MR-PRESSO) and R software (version 4.2.0) were performed for the statistical analysis. Multiple MR methods were employed to assess the causal association between irritability and CVDs. Each method has different assumptions for achieving effective IVs. Inverse-variance weighted (IVW) was used as the main method in MR analyses, as the results of the IVW method are generally most reliable when the total IVs are valid (13). MR-pleiotropy residual sum and outlier (MR-PRESSO) were used to discover outliers in IVW linear regression and correct MR estimates after removing outliers when they exist (18). In addition, weighted median (19) and MR-Egger (20) methods were applied as secondary supplements. Several methods were implemented for sensitivity analysis to confirm the validity and robustness of the results. The heterogeneity of the used SNPs was assessed through Cochrane's *Q* test; if heterogeneity existed (*p* < 0.05), the multiplicative random effects IVW was considered as the main method to obtain a robust and conservative result. Moreover, MR-Egger regression analysis was performed to assess whether a directional pleiotropy of the used IVs existed by its intercept.

### 2.4. Mediation MR analysis

**Figure 2:**
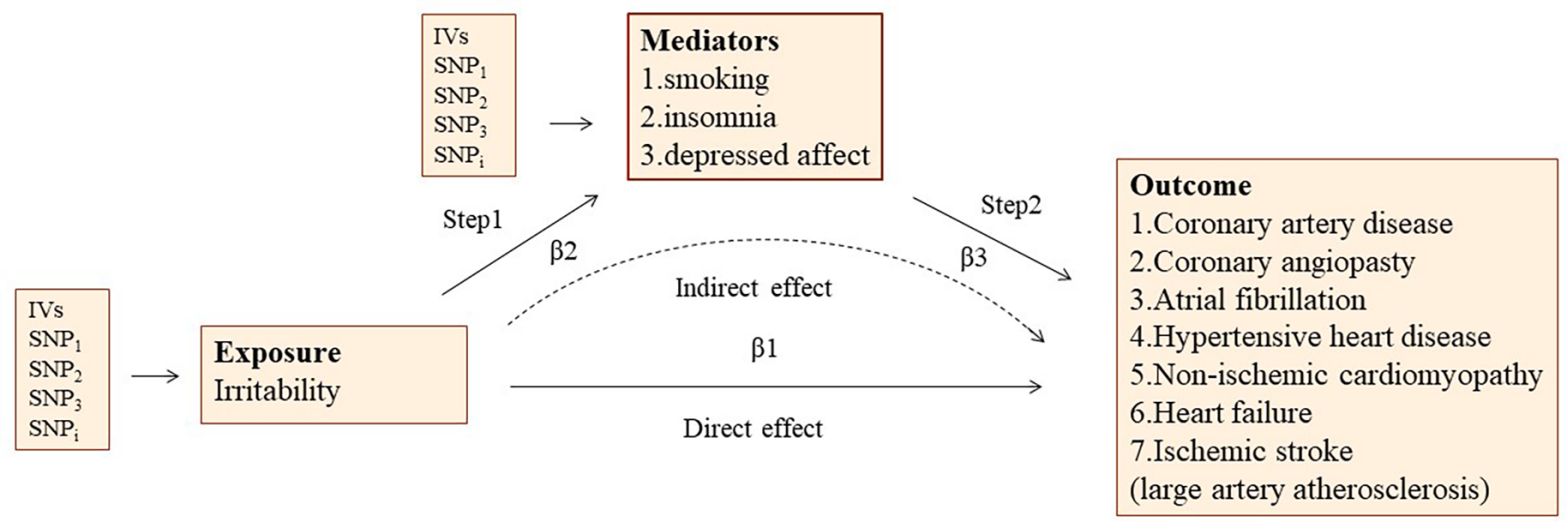
The overview of two-step mediation analysis of irritability on various CVDs via potential mediators. IVs, instrumental variables. In the first step, the causality of irritability on potential mediators was confirmed. In the second step, IVs significantly associated with the mediators are used to assess the causal effect between mediators and the risk of CVDs causally associated with irritability. Direct effect, the effect of irritability on CVD risk after adjusting for the mediators (β1–β2*β3). Indirect effect, the effect of irritability on CVD risk through the mediator (β2*β3).

A design of mediation analysis is shown in Figure 2. Some recognized risk factors of CVD are significantly associated with irritability, such as smoking, insomnia, and depressed affect. Therefore, a two-step MR was used to estimate to what extent the causal relationship was mediated by these risk factors. In the first step, the causality of irritability on these potential mediators was confirmed. In the second step, we used the IVs that were significantly associated with the mediators to assess the causal association between the mediators and the risk of CVDs causally associated with irritability.

## 3. Results

### 3.1. Selection of instrumental variables

Detailed characteristics of SNPs selected to predict irritability are displayed in Supplementary Table S1. All SNPs satisfied established screening criteria in Methods. There was no weak tool bias in the IV strength test since the *F*-statistic of all SNPs >10.

### 3.2. Causal estimates of genetic susceptibility to irritability and CVD risk

**Figure 3:**
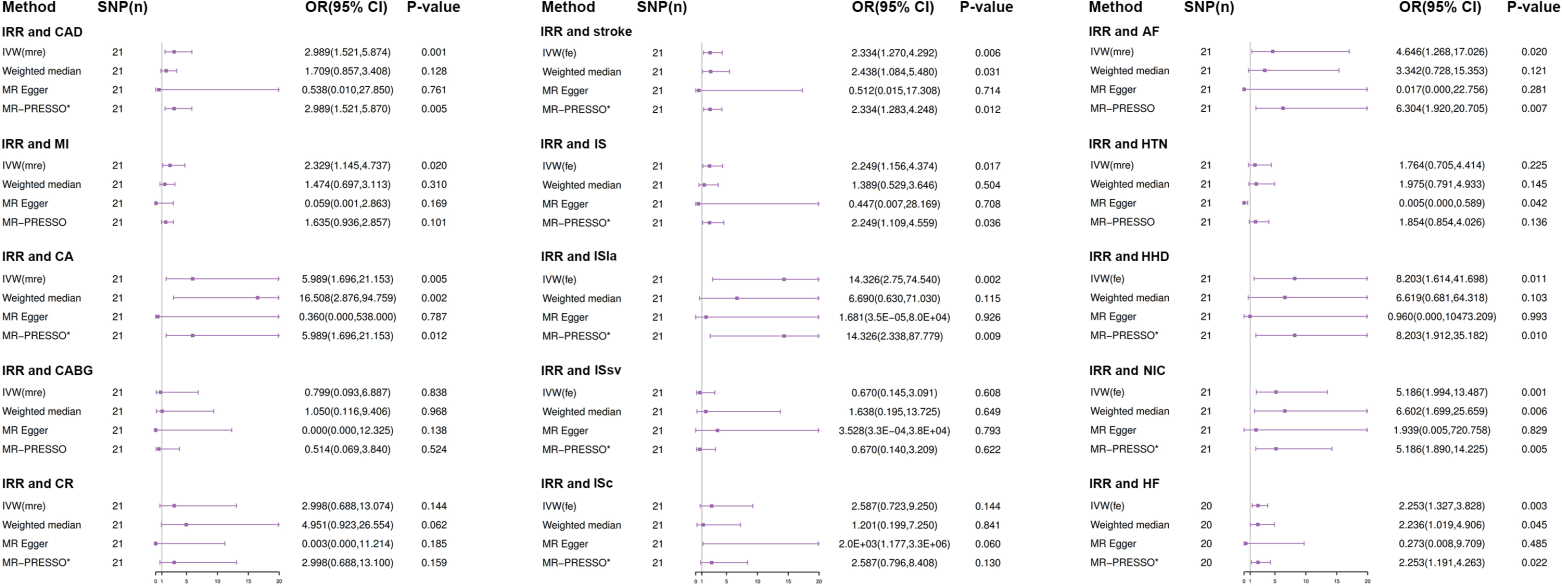
Results from Mendelian randomization analysis of irritability and CVD risk. SNP, Single-nucleotide polymorphism; OR, odd ratio; CI, confidence interval; IVW (fixed effects), fixed effects inverse-variance weighted; IVW (MR effects), multiplicative random inverse-variance weighted; MR-PRESSO, MR-pleiotropy residual sum and outlier; *No outlier was detected; IRR, irritability; CAD, coronary artery disease; MI, myocardial infarction; CA, coronary angioplasty; CABG, coronary artery bypass grafting; CR, coronary revascularization (CA or CABG); AF, atrial fibrillation; HTN, hypertension; HHF, hypertensive heart disease; NIC, non-ischemic cardiomyopathy; HF, heart failure; IS, ischemic stroke; ISla, ischemic stroke (large-artery atherosclerosis); ISsv, ischemic stroke (small-vessel); ISce, ischemic stroke (cardioembolic).

The genetically predicted results between irritability and various CVDs are presented in Figure 3. The IVW analyses as the main analysis method indicated that genetically predicted irritability was positively associated with the risk of several CVDs, including CAD (OR: 2.989, 95% CI: 1.521–5.874, *p* = 0.001), MI (OR: 2.329, 95% CI: 1.145–4.737, *p* = 0.020), CA (OR: 5.989, 95% CI: 1.696–21.153, *p* = 0.005), AF (OR: 4.646, 95% CI: 1.268–17.026, *p* 0.02), HHD (OR: 8.203, 95% CI: 1.614–41.698, *p* = 0.011), NIC (OR: 5.186, 95% CI: 1.994–13.487, *p* = 0.001), HF (OR: 2.253, 95% CI: 1.327–3.828, *p* = 0.003), stroke (OR: 2.334, 95% CI: 1.270–4.292, *p* = 0.006), IS (OR: 2.249, 95% CI: 1.156–4.374, *p* = 0.017), and ISla (OR: 14.326, 95% CI: 2.750–74.540, *p* = 0.002). Nevertheless, insufficient evidence indicated that genetically predicted irritability had a remarkable causal relationship with the prevalence of other CVDs such as CABG, CR, HTN, ISsv, and ISce (Figure 3).

The effect estimator of most statistical analysis models remained generally directionally consistent with IVW analysis, such as weighted median, MR-Egger, and MR-PRESSO. In all the causal analyses, most MR-PRESSO analysis methods did not detect outliers, demonstrating the reliability of the causal relationship estimates in this study (Figure 3). Moreover, scatter plots showed the associations between irritability and various CVD risks (Supplementary Figure S1).

### 3.3. MR sensitivity analysis

In Cochran's *Q* test, we found that there was heterogeneity between the used SNPs in part analyses of causal effects (*p*-values of Cochran's *Q* statistics <0.05) such as CAD, MI, CA, CABG, CR, AF, and HTN. Consequently, multiplicative random effects IVW was used in these causal effect analyses, and IVW analysis was implemented with a fixed-effects model in other effect analyses (Table 2). In addition, apart from HTN, we did not observe that the intercepts of the MR-Egger analysis deviated from zero in all studies, demonstrating that there was no horizontal pleiotropy (Table 2 and Supplementary Figure S1).

**Table 2:** Heterogeneity and pleiotropy tests for the associations between irritability with various cVDs.

MR analysis	SNP (*n*)	Heterogeneity test			Pleiotropy test		
		*Q*	*Q*_df	*Q*-pval	Egger_intercept	SE	*p*
IRR–CAD	21	44.620	20	0.001	0.013	0.015	0.398
IRR–MI	21	42.320	20	0.003	0.028	0.015	0.075
IRR–CA	21	58.657	19	<0.001	0.009	0.018	0.640
IRR–CABG	21	43.676	20	0.002	0.071	0.046	0.141
IRR–CR	21	36.865	20	0.012	0.052	0.031	0.112
IRR–AF	21	36.197	20	0.015	0.040	0.028	0.137
IRR–HTN	21	47.566	20	<0.001	0.045	0.018	0.024
IRR–HHD	21	16.039	20	0.714	0.017	0.036	0.651
IRR–NIC	21	22.291	20	0.325	0.008	0.023	0.744
IRR–HF	20	27.517	19	0.093	0.016	0.014	0.255
IRR–stroke	21	19.323	20	0.501	0.012	0.014	0.402
IRR–IS	21	22.568	20	0.310	0.012	0.016	0.447
IRR–Isla	21	24.162	20	0.235	0.016	0.042	0.697
IRR–ISsv	21	20.989	20	0.398	−0.013	0.036	0.726
IRR–Isce	21	17.111	20	0.646	−0.051	0.029	0.091

IRR, irritability; CAD, coronary artery disease; MI, myocardial infarction; CA, coronary angioplasty; CABG, coronary artery bypass grafting; CR, coronary revascularization (CA or CABG); AF, atrial fibrillation; HTN, hypertension; HHF, hypertensive heart disease; INC, non-ischemic cardiomyopathy; HF, heart failure; IS, ischemic stroke; ISla, ischemic stroke (large-artery atherosclerosis); ISsv, ischemic stroke (small-vessel); ISce, ischemic stroke (cardioembolic).

### 3.4. Mediation MR analysis results

Considering the higher propensity for smoking, insomnia, and depressed affect in the irritable population, these factors may be mediators causing the adverse effect of irritability on various CVDs. We assessed the mediating pathways of smoking initiation, insomnia, and depressed affect in irritability on the risk of several CVDs by performing a two-step MR analysis (Figure 2).

In the first step, we investigated the causal effect of irritability on smoking initiation (IVW: OR: 1.911, 95% CI: 1.276–2.886, *p* = 0.002), irritability on insomnia (IVW: OR: 4.324, 95% CI: 2.359–7.929, *p* < 0.001), and irritability on depressed affect (IVW: OR: 3.327, 95% CI: 2.682–4.127, *p* < 0.001) (Table 3 and Supplementary Table S44).

**Table 3:** The mediation analysis results of irritability on cardiovascular diseases via smoking, insomnia, and depressed affect.

	Number of SNPs	OR (95% CI)	*p*
Irritability on mediators (smoking, insomnia, depressed affect)			
Smoking	21	1.919 (1.276,2.886)	**0** **.** **002**
Insomnia	21	4.324 (2.359,7.929)	**<0**.**001**
Depressed affect	20	3.327 (2.682,4.127)	**<0**.**001**
Smoking on cardiovascular diseases			
Coronary artery disease	74	1.218 (1.104,1.343)	**<0**.**001**
Coronary angioplasty	73	1.443 (1.192,1.748)	**<0**.**001**
Atrial fibrillation	73	1.126 (0.970,1.307)	0.119
Hypertensive heart disease	73	1.034 (0.804,1.330)	0.795
Non-ischemic cardiomyopathy	73	1.164 (1.004,1.349)	**0**.**044**
Heart failure	73	1.197 (1.107,1.295)	**<0**.**001**
Ischemic stroke (large-artery atherosclerosis)	74	1.302 (1.015,1.672)	**0**.**038**
Insomnia on cardiovascular diseases			
Coronary artery disease	22	1.082 (1.015,1.152)	**0**.**015**
Coronary angioplasty	22	1.007 (0.849,1.195)	0.934
Atrial fibrillation	22	1.011 (0.854,1.198)	0.898
Hypertensive heart disease	22	1.004 (0.802,1.255)	0.975
Non-ischemic cardiomyopathy	22	1.180 (1.034,1.347)	**0**.**014**
Heart failure	22	1.038 (0.966,1.116)	0.310
Ischemic stroke (large-artery atherosclerosis)	23	1.125 (0.801,1.580)	0.496
Depressed affect on cardiovascular diseases			
Coronary artery disease	38	1.279 (1.094,1.495)	**0**.**002**
Coronary angioplasty	34	1.480 (0.939,2.333)	0.091
Atrial fibrillation	34	1.352 (0.877,2.084)	0.173
Hypertensive heart disease	34	1.950 (1.074,3.540)	**0**.**028**
Non-ischemic cardiomyopathy	34	1.285 (0.795,2.079)	0.306
Heart failure	34	1.260 (1.055,1.505)	**0**.**011**
Ischemic stroke (large-artery atherosclerosis)	38	1.047 (0.595,1.843)	0.873

Summary MR analysis of the IVW methods for the effect of irritability on mediators and the effect of mediators on cardiovascular diseases. SNP, single-nucleotide polymorphism; OR, odds ratio; CI, confidence interval.

Bold values represent *p* <0.05.

In a second step, we confirmed the causal effect association of mediators on coronary artery disease (CAD), coronary angioplasty (CA), atrial fibrillation (AF), hypertensive heart disease (HHD), non-ischemic cardiomyopathy (NIC), heart failure (HF), and ischemic stroke of large-artery atherosclerosis (ISla). MR-Egger and weighted median estimations were consistent with IVW MR analysis in the direction (Table 3 and Supplementary Table S45–S47).

Finally, we assessed the causal effect of irritability on several CVDs through the mediating effect of smoking (irritability on CAD: mediated proportion = 12%, OR = 1.137; irritability on CA: mediated proportion = 13%, OR = 1.270; irritability on NIC: mediated proportion = 6%, OR = 1.104; irritability on HF: mediated proportion = 14%, OR = 1.125; irritability on ISla: mediated proportion = 6%, OR = 1.188), insomnia (irritability on CAD: mediated proportion = 10%, OR = 1.122; irritability on NIC: mediated proportion = 15%, OR = 1.274), and depressed affect (irritability on CAD: mediated proportion = 27%, OR = 1.344; irritability on CA: mediated proportion = 26%, OR = 1.602; irritability on HHD: mediated proportion = 38%, OR = 2.232; irritability on HF: mediated proportion = 34%, OR = 1.320) (Table 4).

**Table 4:** The mediation effect of irritability on cardiovascular diseases via smoking, insomnia, and depressed affect.

Trait	Total effect (OR)	Direct effect (OR)	Mediation effect (OR)	Proportion mediated (%)
Smoking (IRR on CAD)	2.989	2.629	1.137	12
Smoking (IRR on CA)	5.989	4.715	1.270	13
Smoking (IRR on NIC)	5.186	4.698	1.104	6
Smoking (IRR on HF)	2.253	2.004	1.125	14
Smoking (IRR on ISla)	14.326	12.060	1.188	6
Insomnia (IRR on CAD)	2.989	2.665	1.122	10
Insomnia (IRR on NIC)	5.186	4.070	1.274	15
Depressed affect (IRR on CAD)	2.989	2.224	1.344	27
Depressed affect (IRR on CA)	5.989	3.738	1.602	26
Depressed affect (IRR on HHD)	8.203	3.676	2.232	38
Depressed affect (IRR on HF)	2.253	1.707	1.320	34

Total effect = β1; mediation effect = β2*β3; direct effect = β1–β2*β3; proportion mediated = (β2*β3)/β1. IRR, irritability; CAD, coronary artery disease; CA, coronary angioplasty; AF, atrial fibrillation; HHF, hypertensive heart disease; INC, non-ischemic cardiomyopathy; HF, heart failure; ISla, ischemic stroke (large-artery atherosclerosis); OR, odds ratio.

## 4. Discussion

To our knowledge, many observational studies have found evidence of a relationship between irritability and CVD risk. With the various MR analyses, we systematically confirmed the potential causal relationships between irritability susceptibility and a broad range of CVD risks for the first time. The MR evidence of our study indicates that genetic predisposition to irritability has adverse causal associations with many CVD risks, including CAD, AF, HHD, NIC, HF, and ISla. In addition, insufficient MR evidence supports a potential causal association between genetic predisposition to irritability and the risk of HTN, ISsv, and ISce. Given the fact that the irritable population has a high propensity for smoking (21), insomnia (22), and depressed affect (23, 24), the results of the mediation MR analysis in our study show that these factors play an important role in the process of irritability leading to cardiovascular disease. The MR results are robust due to the use of different MR analysis methods and strong instruments.

Recently, increasing attention has been focused on the role of psychosocial factors in CVDs. Irritability is generally best described by impulsiveness, anger, and hostility and is a tendency to overreact to aversive stimuli with negative affect. Observational studies have shown that irritability is significantly related to an increased risk of various CVDs, which was supported by the present MR analysis.

In specific CVDs, our estimates were consistent with the findings of previous studies.

A cohort study of 225 patients with suspected CAD found that high irritable scores had a direct relationship with the presence of significant luminal stenosis (6). Another cohort study, including 506 women with suspected CAD, reported that hostile affect and aggressive responses were associated with poorer event-free survival and a higher risk of adverse events (25). In addition, a prospective study indicated that anger increased the risk of CAD during an average of 10.9 years of follow-up (8). We used MR analysis to further prove their relevance. Irritability increased the risk of CAD and MI; also, irritable people are more likely to accept to undergo coronary angioplasty. Moreover, we found that smoking, insomnia, and depressed affect mediated the occurrence of CAD.

In a study involving 6,292 Japanese people, it was found that men with higher “anger-in” scores had a 50% increased risk of hypertension compared with those with lower scores, but no such association was found in women (26). A recent study suggested that there should be focused attention on anger as a potential risk factor for hypertension (27). We did not find any association between irritability and hypertension in our MR study, but we found that irritable people had a tendency to smoke. Men are more inclined to smoke and smoking is a risk factor for hypertension. This may partly explain why the association of anger with hypertension is significant only in men. Moreover, a study confirmed that anger may cause left ventricular hypertrophy in hypertensive patients (28). We confirmed the causal relationship between irritability and hypertensive heart disease. Although irritability is not directly related to hypertension, irritability-related bad behaviors are risk factors for hypertension. They may play a significant mediatory role in the development of hypertension.

A meta-analysis demonstrated that the incidence of AF was increased by 15% for patients with anger (9). A Swedish cohort study also found that anger increased the risk of AF (29). In contrast, another study showed that no significant associations were observed between anger, anxiety, and chronic stress and the development of AF (30). Our results of MR analysis provided evidence that genetically predicted irritability increased the incidence of AF.

A Swedish cohort study found that anger increased the risk of HF (HR = 1.19) (29). A prospective study containing 13,171 black and white participants found that the incidence of HF was greater among participants with high trait anger (HR: 1.44) compared with those with low or moderate trait anger. Adjustment for comorbidities and depressive symptoms attenuated the estimated relative hazard in men to 1.26 (10). An angry temperament may cause a rise in the levels of catecholamine, resulting in transient left ventricular dysfunction. Recently, anger has been found associated with worse resting left ventricular diastolic pressure (31). These findings are consistent with our findings. Our results indicated that irritability increased the risk of NIC and HF. The potential association of irritability with HF was partly mediated by depressed affect (34%).

Anger has been suggested as a triggering factor for stroke (32). A study reported that Finnish male patients with the highest level of expressed anger were at twice the risk of stroke of men who reported the lowest level of anger, after adjustments for risk factors (12). Another study including 13,851 black and white participants obtained similar results that participants with high trait anger had a 2.82 times greater risk for hemorrhagic and ischemic strokes combined and a 2.93 times greater risk for ischemic strokes alone than their counterparts with low trait anger during an average of 6.4 years follow-up (33). Our results demonstrated that irritability increased the risk of total stroke, IS, and ISla. Smoking plays a part of the intermediary role in the process of disease formation occurrence of ISla.

Several hypotheses have explained the association between an angry temperament and CVD. Highly hostile individuals constantly have a high level of catecholamine and cortisol secretion in response to anger-provoking stimuli (5) and they display increased cortisol excretion during daytime hours along with an activation of the hypothalamic–hypophyseal axis (34, 35). This causes endothelial injury, increased vascular rigidity, and the disruption of vulnerable plaques. In addition, an angry temperament may lead to augmented platelet aggregation, decreased fibrinolytic potential, and increased plasma viscosity by enhancing inflammatory and prothrombotic responses (36). Moreover, behavioral factors may also have a significant impact on the relationship between irritability and CVD risk. Our two-step MR analysis revealed that irritability is significantly linked to an increased risk of smoking, insomnia, and depressed affect, which have all been identified as major risk factors for CVD. Controlling these behavioral factors in irritable individuals could be an effective measure to reduce the incidence of CVD in this population. These mechanisms theoretically can help us explain our observed association between irritability and various CVDs, but more comprehensive and reliable studies are needed to confirm these hypotheses.

There are several noteworthy advantages in our study. The current evidence on the relationship between irritability and the risk of CVDs is almost based on observational studies, and the findings may be limited by sample size, residual confusion, and reverse causality. Due to the random allocation of genetic variants, the MR analysis can help obtain a more robust causal effect between exposure and outcome by the avoidance of these limitations. In the present MR study, multiple MR methods were performed to confirm the causality between irritability and the risk of CVDs for the first time. Moreover, several sensitivity analyses were used to estimate heterogeneity and pleiotropy. Lastly, we used a two-step MR to perform a mediation analysis and indicated that the causal relationship of irritability on various CVDs may be mediated by smoking, insomnia, and depression.

Inevitably, certain limitations should be acknowledged. First, all participants involved in our study were Europeans. However, risk factors, prevalence, and mortality are different in different ethnicities. Therefore, in other ethnicities and populations, our findings possibly cannot explain the potential causality of irritability and CVDs. In addition, we tried our best to avoid sample overlap, but the datasets of CAD and MI were partly prepared from UKB data, which may cause slightly biased MR estimates. Therefore, we further confirmed the causality of irritability by highlighting the necessity of angioplasty intervention, because coronary angioplasty is the main diagnosis and treatment method for patients with coronary heart disease. Finally, a part MR-PRESSO analysis method detected outliers (AF, MI, and CABG), but the result of the MR-Egger regression analysis showed that there was no horizontal pleiotropy. Therefore, the results of the MR analysis should be interpreted with caution.

## 5. Conclusions

This MR study supports the first genetic evidence that genetically predicted irritability may be implicated in the development of CVD. We found the existence of adverse associations between irritability and the risk of CAD, AF, HF, HHD, NIC, and ISla, and these associations may partly be driven by smoking, insomnia, and depressed affect. Our results deliver a viewpoint that the identification of irritability as a risk factor or trigger of CVD is important because more early active interventions to manage an individual's anger may prevent the occurrence of cardiovascular adverse events. Moreover, correcting the unhealthy lifestyle habits of irritable individuals is an effective clinical intervention for reducing the risk of CVD.

## Data Availability

The original contributions presented in the study are included in the article/**Supplementary Material**, and further inquiries can be directed to the corresponding author.
