# Supplementary material for: A causal relationship between irritability and cardiovascular diseases: a Mendelian randomization study
Source: Front Cardiovasc Med. 2023 May 30;10:1174329. doi: 10.3389/fcvm.2023.1174329 (PMC10267866; doi:10.3389/fcvm.2023.1174329)
Supplement: Supplementary file 1 [file Datasheet1.docx]

Supplementary Material

Supplementary Table 1: Characteristics of SNPs used as instrumental variables for irritability.

Supplementary Table 2: Characteristics of SNPs used as instrumental variables for smoking.

Supplementary Table 3: Characteristics of SNPs used as instrumental variables for insomnia.

Supplementary Table 4: Characteristics of SNPs used as instrumental variables for depressed affect.

Supplementary Table 5: Mendelian randomization analysis of irritability on CAD.

Supplementary Table 6: Mendelian randomization analysis of irritability on MI.

Supplementary Table 7: Mendelian randomization analysis of irritability on CA.

Supplementary Table 8: Mendelian randomization analysis of irritability on CABG.

Supplementary Table 9: Mendelian randomization analysis of irritability on RE.

Supplementary Table 10: Mendelian randomization analysis of irritability on AF.

Supplementary Table 11: Mendelian randomization analysis of irritability on HTN.

Supplementary Table 12: Mendelian randomization analysis of irritability on HHD.

Supplementary Table 13: Mendelian randomization analysis of irritability on NIC.

Supplementary Table 14: Mendelian randomization analysis of irritability on HF.

Supplementary Table 15: Mendelian randomization analysis of irritability on stroke.

Supplementary Table 16: Mendelian randomization analysis of irritability on IS.

Supplementary Table 17: Mendelian randomization analysis of irritability on ISla.

Supplementary Table 18: Mendelian randomization analysis of irritability on ISsv.

Supplementary Table 19: Mendelian randomization analysis of irritability on ISce.

Supplementary Table 20: Mendelian randomization analysis of irritability on smoking.

Supplementary Table 21: Mendelian randomization analysis of irritability on insomnia.

Supplementary Table 22: Mendelian randomization analysis of irritability on depressed affect.

Supplementary Table 23: Mendelian randomization analysis of smoking on CAD.

Supplementary Table 24: Mendelian randomization analysis of smoking on CA.

Supplementary Table 25: Mendelian randomization analysis of smoking on AF.

Supplementary Table 26: Mendelian randomization analysis of smoking on HHD.

Supplementary Table 27: Mendelian randomization analysis of smoking on NIC.

Supplementary Table 28: Mendelian randomization analysis of smoking on HF.

Supplementary Table 29: Mendelian randomization analysis of smoking on ISla.

Supplementary Table 30: Mendelian randomization analysis of insomnia on CAD.

Supplementary Table 31: Mendelian randomization analysis of insomnia on CA.

Supplementary Table 32: Mendelian randomization analysis of insomnia on AF.

Supplementary Table 33: Mendelian randomization analysis of insomnia on HHD.

Supplementary Table 34: Mendelian randomization analysis of insomnia on NIC.

Supplementary Table 35: Mendelian randomization analysis of insomnia on HF.

Supplementary Table 36: Mendelian randomization analysis of insomnia on ISla.

Supplementary Table 37: Mendelian randomization analysis of depressed affect on CAD.

Supplementary Table 38: Mendelian randomization analysis of depressed affect on CA.

Supplementary Table 39: Mendelian randomization analysis of depressed affect on AF.

Supplementary Table 40: Mendelian randomization analysis of depressed affect on HHD.

Supplementary Table 41: Mendelian randomization analysis of depressed affect on NIC.

Supplementary Table 42: Mendelian randomization analysis of depressed affect on HF.

Supplementary Table 43: Mendelian randomization analysis of depressed affect on ISla.

Supplementary Table 44. MR results for the relationship between irritability and mediators

Supplementary Table 45. MR results for the relationship between smoking and CVDs

Supplementary Table 46. MR results for the relationship between insomnia and CVDs

Supplementary Table 47. MR results for the relationship between depressed affect and CVDs

Supplementary Figure 1. Scatter plot using all IVs of irritability on CVDs.

Supplementary Figure 2. Scatter plot using all IVs of smoking on CVDs.

Supplementary Figure 3. Scatter plot using all IVs of insomnia on CVDs.

Supplementary Figure 4. Scatter plot using all IVs of depressed affect on CVDs.

Abbreviation: CVD, cardiovascular disease; CAD, coronary artery disease; MI, myocardial infarction; CA, coronary angiopasty; CABG, coronary artery bypass grafting; CR, coronary revascularization (CA or CABG); AF, atrial fibrillation; HTN, hypertension; HHF, hypertensive heart disease; INC, non-ischemic cardiomyopathy; HF, heart failure; IS, ischemic stroke; ISla, ischemic stroke (large artery atherosclerosis); ISsv, ischemic stroke (small-vessel); ISce, ischemic stroke (cardioembolic); MR, Mendelian randomization; A1= effect_allele; A2= other_allele.

EAF: Effect allele frequency; SE：Standard error. F-statistics= (Beta/SE)^2^

Table 1: Characteristics of SNPs used as instrumental variables for irritability.

|  | SNP | chr | pos | effect_  allele | other_  allele | EAF | Beta | SE | pval | F-statistic |
| --- | --- | --- | --- | --- | --- | --- | --- | --- | --- | --- |
| 1 | rs871852 | 2 | 43674354 | C | G | 0.4938 | 0.0062 | 0.0011 | 3.05E-08 | 30.68 |
| 2 | rs343936 | 2 | 44992550 | C | T | 0.1685 | -0.0082 | 0.0015 | 3.97E-08 | 30.17 |
| 3 | rs12612050 | 2 | 122926268 | A | G | 0.4548 | 0.0062 | 0.0011 | 3.91E-08 | 30.20 |
| 4 | rs3772556 | 3 | 105249211 | T | C | 0.7062 | -0.0071 | 0.0012 | 6.47E-09 | 33.69 |
| 5 | rs10463587 | 5 | 107744061 | G | C | 0.2191 | 0.0079 | 0.0014 | 4.70E-09 | 34.31 |
| 6 | rs9390366 | 6 | 100864938 | G | T | 0.2262 | 0.0073 | 0.0013 | 4.82E-08 | 29.79 |
| 7 | rs776472 | 7 | 114352862 | T | C | 0.5664 | -0.0067 | 0.0011 | 2.97E-09 | 35.21 |
| 8 | rs16884419 | 8 | 89579649 | A | G | 0.2363 | 0.0087 | 0.0013 | 4.44E-11 | 43.41 |
| 9 | rs999483 | 9 | 135301389 | G | T | 0.2499 | 0.0077 | 0.0013 | 2.27E-09 | 35.73 |
| 10 | rs10905638 | 10 | 9951081 | C | G | 0.4435 | 0.0064 | 0.0011 | 1.15E-08 | 32.58 |
| 11 | rs58446129 | 13 | 66582410 | T | C | 0.1462 | 0.0096 | 0.0016 | 1.08E-09 | 37.17 |
| 12 | rs4903249 | 14 | 75083881 | T | C | 0.4877 | 0.0063 | 0.0011 | 1.85E-08 | 31.65 |
| 13 | rs7181641 | 15 | 61744669 | G | A | 0.1483 | 0.0094 | 0.0016 | 2.62E-09 | 35.45 |
| 14 | rs199497 | 17 | 44866602 | C | T | 0.1745 | 0.0098 | 0.0015 | 1.70E-10 | 40.79 |
| 15 | rs34340612 | 18 | 42232686 | A | G | 0.2160 | -0.0079 | 0.0014 | 6.83E-09 | 33.58 |
| 16 | rs7235757 | 18 | 53067954 | A | G | 0.3113 | 0.0081 | 0.0012 | 2.13E-11 | 44.85 |
| 17 | rs17781446 | 18 | 26678108 | T | C | 0.0690 | 0.0121 | 0.0022 | 4.83E-08 | 29.79 |
| 18 | rs6058104 | 20 | 33271052 | A | G | 0.8241 | -0.0101 | 0.0015 | 5.69E-12 | 47.44 |
| 19 | rs20551 | 22 | 41548008 | G | A | 0.2880 | 0.0071 | 0.0012 | 6.98E-09 | 33.54 |
| 20 | rs4308307 | 3 | 49193216 | T | C | 0.6216 | -0.0067 | 0.0012 | 5.72E-09 | 33.93 |
| 21 | rs7805419 | 7 | 12282451 | C | T | 0.4138 | 0.0062 | 0.0011 | 4.83E-08 | 29.79 |

Table 2: Characteristics of SNPs used as instrumental variables for smoking.

|  | SNP | chr | pos | effect_  allele | other_  allele | EAF | Beta | SE | pval | F-statistic |
| --- | --- | --- | --- | --- | --- | --- | --- | --- | --- | --- |
| 1 | rs3001723 | 1 | 44037685 | A | G | 0.321 | 0.034 | 0.004 | 8.12E-18 | 73.90 |
| 2 | rs7555507 | 1 | 73766037 | T | C | 0.496 | -0.024 | 0.004 | 1.14E-11 | 46.10 |
| 3 | rs12025237 | 1 | 154205120 | C | A | 0.124 | -0.033 | 0.005 | 6.52E-10 | 38.20 |
| 4 | rs2050586 | 1 | 87905828 | C | G | 0.355 | -0.021 | 0.004 | 3.00E-08 | 30.70 |
| 5 | rs4674993 | 2 | 226332033 | G | A | 0.207 | -0.025 | 0.004 | 1.32E-08 | 32.30 |
| 6 | rs578584 | 2 | 45143175 | T | A | 0.605 | 0.029 | 0.004 | 1.50E-15 | 63.60 |
| 7 | rs35702515 | 2 | 137542847 | T | G | 0.162 | 0.025 | 0.004 | 2.43E-09 | 35.60 |
| 8 | rs12474587 | 2 | 162802993 | T | G | 0.404 | 0.028 | 0.004 | 1.25E-14 | 59.50 |
| 9 | rs2107300 | 2 | 200937901 | G | C | 0.845 | -0.027 | 0.005 | 3.27E-08 | 30.50 |
| 10 | rs7585579 | 2 | 60024857 | G | C | 0.505 | 0.022 | 0.004 | 1.88E-09 | 36.10 |
| 11 | rs1445649 | 2 | 155682556 | C | T | 0.525 | 0.024 | 0.004 | 1.68E-11 | 45.30 |
| 12 | rs11712680 | 3 | 75009019 | C | A | 0.174 | -0.027 | 0.005 | 3.51E-09 | 34.90 |
| 13 | rs1869243 | 3 | 5724536 | C | T | 0.481 | 0.020 | 0.004 | 2.97E-08 | 30.70 |
| 14 | rs962625 | 4 | 28473524 | G | A | 0.240 | 0.024 | 0.004 | 4.37E-09 | 34.50 |
| 15 | rs10001365 | 4 | 147797214 | A | G | 0.405 | -0.025 | 0.004 | 6.65E-12 | 47.10 |
| 16 | rs1160685 | 4 | 94052854 | G | C | 0.478 | 0.021 | 0.004 | 7.20E-09 | 33.50 |
| 17 | rs6893752 | 5 | 60374912 | G | A | 0.766 | -0.024 | 0.004 | 3.25E-09 | 35.00 |
| 18 | rs12186738 | 5 | 103816655 | T | G | 0.154 | -0.033 | 0.005 | 3.42E-11 | 43.90 |
| 19 | rs1385108 | 5 | 154839646 | T | C | 0.239 | 0.025 | 0.004 | 3.00E-09 | 35.20 |
| 20 | rs4044321 | 5 | 166989513 | G | A | 0.642 | -0.028 | 0.004 | 6.08E-14 | 56.30 |
| 21 | rs4352629 | 5 | 87756821 | T | C | 0.492 | -0.028 | 0.004 | 1.22E-14 | 59.50 |
| 22 | rs72789632 | 5 | 106834363 | T | C | 0.120 | -0.033 | 0.005 | 5.02E-10 | 38.70 |
| 23 | rs9401770 | 6 | 98748008 | A | G | 0.273 | 0.028 | 0.004 | 3.47E-12 | 48.40 |
| 24 | rs222449 | 6 | 52916062 | T | A | 0.793 | -0.025 | 0.004 | 1.08E-08 | 32.70 |
| 25 | rs10498846 | 6 | 67405337 | T | C | 0.473 | 0.021 | 0.004 | 6.62E-09 | 33.60 |
| 26 | rs10279261 | 7 | 133589846 | A | G | 0.619 | -0.021 | 0.004 | 5.00E-09 | 34.20 |
| 27 | rs10260968 | 7 | 1889773 | A | G | 0.597 | -0.020 | 0.004 | 1.75E-08 | 31.70 |
| 28 | rs4236259 | 7 | 1708080 | G | T | 0.499 | -0.025 | 0.004 | 3.35E-12 | 48.50 |
| 29 | rs2140114 | 7 | 3407568 | T | C | 0.518 | -0.023 | 0.004 | 4.70E-10 | 38.80 |
| 30 | rs3801289 | 7 | 96638267 | C | A | 0.351 | -0.022 | 0.004 | 3.74E-09 | 34.80 |
| 31 | rs1565735 | 8 | 27426077 | A | T | 0.212 | -0.038 | 0.004 | 3.42E-17 | 71.10 |
| 32 | rs1899896 | 8 | 93201036 | T | C | 0.286 | 0.026 | 0.004 | 1.04E-11 | 46.30 |
| 33 | rs13261666 | 8 | 59814666 | T | G | 0.522 | -0.027 | 0.004 | 3.90E-14 | 57.20 |
| 34 | rs12545053 | 8 | 65073605 | G | A | 0.397 | 0.020 | 0.004 | 2.43E-08 | 31.10 |
| 35 | rs2631024 | 8 | 91995577 | G | A | 0.737 | -0.023 | 0.004 | 1.18E-08 | 32.50 |
| 36 | rs4543592 | 9 | 3014254 | C | T | 0.468 | 0.022 | 0.004 | 7.46E-10 | 37.90 |
| 37 | rs2378662 | 9 | 86707289 | A | G | 0.556 | 0.021 | 0.004 | 4.16E-09 | 34.50 |
| 38 | rs10114490 | 9 | 11070165 | A | G | 0.198 | -0.026 | 0.005 | 1.81E-08 | 31.70 |
| 39 | rs10905461 | 10 | 8803551 | C | T | 0.718 | -0.024 | 0.004 | 7.35E-09 | 33.40 |
| 40 | rs7921378 | 10 | 63674885 | C | G | 0.463 | -0.025 | 0.004 | 8.26E-13 | 51.20 |
| 41 | rs12356821 | 10 | 104563808 | C | G | 0.140 | 0.039 | 0.005 | 6.27E-15 | 60.80 |
| 42 | rs9423279 | 10 | 125680419 | G | C | 0.641 | -0.021 | 0.004 | 3.21E-08 | 30.60 |
| 43 | rs7929518 | 11 | 85980958 | G | A | 0.765 | 0.024 | 0.004 | 1.56E-08 | 32.00 |
| 44 | rs4523689 | 11 | 7950797 | G | A | 0.408 | -0.021 | 0.004 | 1.55E-08 | 32.00 |
| 45 | rs11057005 | 12 | 16748721 | G | A | 0.430 | -0.021 | 0.004 | 4.85E-09 | 34.20 |
| 46 | rs1971318 | 12 | 121389500 | T | C | 0.141 | 0.029 | 0.005 | 7.06E-09 | 33.50 |
| 47 | rs7322872 | 13 | 100548329 | T | C | 0.782 | -0.026 | 0.004 | 3.58E-09 | 34.80 |
| 48 | rs3904512 | 13 | 38357471 | A | G | 0.429 | -0.021 | 0.004 | 3.23E-09 | 35.00 |
| 49 | rs9540729 | 13 | 66947124 | T | A | 0.501 | -0.020 | 0.004 | 3.82E-08 | 30.20 |
| 50 | rs76214862 | 14 | 29500130 | C | A | 0.202 | -0.025 | 0.005 | 3.99E-08 | 30.20 |
| 51 | rs12441907 | 15 | 83922387 | A | C | 0.186 | -0.029 | 0.005 | 1.06E-10 | 41.70 |
| 52 | rs4785836 | 16 | 65604652 | C | T | 0.398 | -0.020 | 0.004 | 2.26E-08 | 31.30 |
| 53 | rs7197072 | 16 | 717085 | T | C | 0.238 | -0.025 | 0.004 | 2.77E-09 | 35.30 |
| 54 | rs1050847 | 16 | 87443734 | T | C | 0.505 | -0.022 | 0.004 | 1.67E-09 | 36.30 |
| 55 | rs4781977 | 16 | 17572674 | C | T | 0.205 | -0.024 | 0.004 | 4.54E-08 | 29.90 |
| 56 | rs11078713 | 17 | 7795972 | G | A | 0.454 | -0.020 | 0.004 | 2.23E-08 | 31.30 |
| 57 | rs7224742 | 17 | 30657058 | T | C | 0.595 | -0.021 | 0.004 | 1.43E-08 | 32.10 |
| 58 | rs6508144 | 18 | 50026142 | G | C | 0.563 | -0.021 | 0.004 | 7.97E-09 | 33.30 |
| 59 | rs11872397 | 18 | 72535282 | A | G | 0.252 | -0.025 | 0.004 | 1.43E-09 | 36.60 |
| 60 | rs72896886 | 18 | 42632652 | C | G | 0.144 | -0.027 | 0.005 | 2.75E-08 | 30.90 |
| 61 | rs76608582 | 19 | 4474725 | A | C | 0.039 | -0.050 | 0.008 | 1.94E-09 | 36.00 |
| 62 | rs117143374 | 21 | 40555561 | C | T | 0.120 | 0.029 | 0.005 | 2.76E-08 | 30.90 |
| 63 | rs134529 | 22 | 28781758 | C | T | 0.349 | -0.020 | 0.004 | 4.85E-08 | 29.80 |
| 64 | rs7969559 | 12 | 69655167 | G | A | 0.688 | -0.024 | 0.004 | 7.31E-10 | 37.90 |
| 65 | rs1154693 | 3 | 117804154 | G | A | 0.856 | 0.033 | 0.005 | 3.12E-11 | 44.10 |
| 66 | rs240963 | 6 | 111644332 | C | T | 0.836 | -0.041 | 0.005 | 2.16E-17 | 72.00 |
| 67 | rs266047 | 2 | 104088751 | A | G | 0.529 | -0.031 | 0.004 | 3.36E-16 | 66.60 |
| 68 | rs6433897 | 2 | 182034448 | C | T | 0.754 | 0.022 | 0.004 | 3.16E-08 | 30.60 |
| 69 | rs6669839 | 1 | 50625979 | T | C | 0.204 | 0.026 | 0.004 | 3.36E-09 | 35.00 |
| 70 | rs13030994 | 2 | 146143090 | A | G | 0.485 | 0.036 | 0.004 | 3.56E-24 | 103.00 |
| 71 | rs13145728 | 4 | 140927812 | C | G | 0.358 | -0.023 | 0.004 | 2.14E-10 | 40.30 |
| 72 | rs1435741 | 15 | 47935843 | A | G | 0.425 | 0.029 | 0.004 | 2.64E-16 | 67.10 |
| 73 | rs1555445 | 20 | 31175258 | T | A | 0.337 | 0.023 | 0.004 | 3.65E-09 | 34.80 |
| 74 | rs2046850 | 1 | 210304319 | T | C | 0.187 | -0.025 | 0.004 | 3.03E-08 | 30.70 |
| 75 | rs2186122 | 1 | 66470206 | T | A | 0.561 | 0.026 | 0.004 | 3.61E-13 | 52.80 |

Table 3: Characteristics of SNPs used as instrumental variables for insomnia.

|  | SNP | chr | pos | effect_  allele | other_  allele | EAF | Beta | SE | pval | F-statistic |
| --- | --- | --- | --- | --- | --- | --- | --- | --- | --- | --- |
| 1 | rs865199 | 1 | 208028592 | G | A | 0.932 | 0.086 | 0.019 | 4.08E-06 | 21.23 |
| 2 | rs3923386 | 1 | 212042517 | T | C | 0.521 | -0.044 | 0.009 | 2.93E-06 | 21.86 |
| 3 | rs2206301 | 1 | 22492738 | C | T | 0.625 | -0.050 | 0.010 | 3.22E-07 | 26.12 |
| 4 | rs113851554 | 2 | 66750564 | T | G | 0.056 | 0.178 | 0.020 | 2.14E-18 | 76.56 |
| 5 | rs145265872 | 2 | 181285398 | T | C | 0.046 | -0.107 | 0.023 | 3.02E-06 | 21.80 |
| 6 | rs116523352 | 3 | 154335733 | T | G | 0.019 | -0.174 | 0.036 | 1.76E-06 | 22.84 |
| 7 | rs6790885 | 3 | 94947003 | A | G | 0.503 | -0.044 | 0.010 | 3.59E-06 | 21.47 |
| 8 | rs1552291 | 4 | 39642737 | G | A | 0.629 | 0.047 | 0.010 | 1.93E-06 | 22.66 |
| 9 | rs114740697 | 5 | 26633947 | G | A | 0.026 | 0.143 | 0.031 | 4.58E-06 | 21.00 |
| 10 | rs72802225 | 5 | 151249430 | G | A | 0.014 | 0.204 | 0.043 | 1.89E-06 | 22.71 |
| 11 | rs115780514 | 5 | 112400135 | G | T | 0.019 | -0.166 | 0.034 | 1.17E-06 | 23.63 |
| 12 | rs140007258 | 6 | 53255355 | A | T | 0.017 | 0.177 | 0.038 | 2.85E-06 | 21.91 |
| 13 | rs1547668 | 6 | 33775446 | G | A | 0.796 | -0.054 | 0.012 | 3.61E-06 | 21.46 |
| 14 | rs79278977 | 8 | 95227580 | T | C | 0.014 | -0.194 | 0.042 | 3.53E-06 | 21.50 |
| 15 | rs62515690 | 8 | 51387554 | A | G | 0.015 | 0.199 | 0.043 | 4.56E-06 | 21.02 |
| 16 | rs2673609 | 8 | 133388216 | G | C | 0.360 | 0.045 | 0.010 | 4.98E-06 | 20.85 |
| 17 | rs1994140 | 11 | 48387186 | G | A | 0.565 | -0.046 | 0.009 | 9.99E-07 | 23.93 |
| 18 | rs112383419 | 11 | 113116100 | A | G | 0.061 | 0.094 | 0.020 | 2.40E-06 | 22.25 |
| 19 | rs117149835 | 12 | 113008891 | C | T | 0.013 | 0.198 | 0.043 | 3.49E-06 | 21.53 |
| 20 | rs7332332 | 13 | 53972489 | G | A | 0.497 | -0.044 | 0.009 | 3.52E-06 | 21.51 |
| 21 | rs147256205 | 14 | 53969392 | A | C | 0.014 | 0.188 | 0.041 | 4.63E-06 | 20.99 |
| 22 | rs208827 | 15 | 89942331 | G | C | 0.636 | 0.051 | 0.010 | 2.24E-07 | 26.82 |
| 23 | rs149251474 | 18 | 62570476 | G | C | 0.022 | 0.158 | 0.034 | 2.77E-06 | 21.97 |
| 24 | rs738475 | 22 | 28391786 | G | C | 0.259 | 0.051 | 0.011 | 2.54E-06 | 22.14 |

Table 4: Characteristics of SNPs used as instrumental variables for depressed affect.

|  | SNP | chr | pos | effect_  allele | other_  allele | EAF | Beta | SE | pval | F-statistic |
| --- | --- | --- | --- | --- | --- | --- | --- | --- | --- | --- |
| 1 | rs75650221 | 1 | 174421994 | T | C | 0.038 | -0.036 | 0.006 | 3.08E-09 | 35.14 |
| 2 | rs11209175 | 1 | 68374828 | T | C | 0.635 | -0.015 | 0.002 | 1.65E-09 | 36.35 |
| 3 | rs12137936 | 1 | 72720357 | G | C | 0.314 | 0.014 | 0.002 | 2.94E-08 | 30.74 |
| 4 | rs12030991 | 1 | 107226359 | C | G | 0.762 | 0.015 | 0.003 | 3.05E-08 | 30.67 |
| 5 | rs76923064 | 2 | 157157148 | C | T | 0.013 | 0.056 | 0.010 | 3.24E-08 | 30.57 |
| 6 | rs11693031 | 2 | 212677931 | G | A | 0.321 | -0.016 | 0.003 | 4.59E-10 | 38.84 |
| 7 | rs2042555 | 2 | 148555489 | A | G | 0.415 | 0.014 | 0.002 | 9.45E-10 | 37.42 |
| 8 | rs62172117 | 2 | 144168667 | A | G | 0.357 | -0.014 | 0.002 | 6.19E-09 | 33.79 |
| 9 | rs59382200 | 3 | 116959274 | G | A | 0.401 | -0.016 | 0.002 | 2.44E-11 | 44.58 |
| 10 | rs9858071 | 3 | 16845406 | C | T | 0.288 | 0.016 | 0.003 | 3.25E-10 | 39.53 |
| 11 | rs836927 | 3 | 107201428 | A | C | 0.427 | 0.015 | 0.002 | 4.11E-10 | 39.08 |
| 12 | rs9852417 | 3 | 65161154 | A | C | 0.590 | -0.013 | 0.002 | 2.84E-08 | 30.79 |
| 13 | rs13122395 | 4 | 101750653 | A | G | 0.326 | 0.014 | 0.002 | 3.63E-08 | 30.36 |
| 14 | rs6818081 | 4 | 176840816 | T | C | 0.148 | 0.018 | 0.003 | 2.83E-08 | 30.83 |
| 15 | rs10020288 | 4 | 28687842 | A | G | 0.360 | -0.013 | 0.002 | 2.56E-08 | 31.04 |
| 16 | rs77087420 | 4 | 123122856 | G | A | 0.055 | -0.033 | 0.005 | 1.06E-10 | 41.71 |
| 17 | rs7714426 | 5 | 120019068 | G | C | 0.849 | -0.018 | 0.003 | 1.41E-08 | 32.18 |
| 18 | rs55965054 | 5 | 24032378 | T | C | 0.559 | -0.014 | 0.002 | 2.67E-09 | 35.43 |
| 19 | rs6900114 | 6 | 64809537 | A | G | 0.265 | -0.015 | 0.003 | 2.30E-08 | 31.20 |
| 20 | rs28893517 | 6 | 29565373 | G | A | 0.068 | 0.028 | 0.005 | 5.16E-10 | 38.62 |
| 21 | rs60393230 | 9 | 122655283 | G | A | 0.596 | 0.014 | 0.002 | 3.57E-09 | 34.86 |
| 22 | rs10156548 | 9 | 23318433 | C | G | 0.643 | -0.018 | 0.002 | 2.55E-13 | 53.55 |
| 23 | rs2149351 | 9 | 120501644 | G | T | 0.758 | -0.016 | 0.003 | 2.27E-09 | 35.71 |
| 24 | rs7912226 | 10 | 107652144 | A | T | 0.397 | -0.015 | 0.002 | 8.12E-10 | 37.70 |
| 25 | rs35738585 | 11 | 113386347 | G | T | 0.434 | -0.017 | 0.002 | 1.73E-13 | 54.29 |
| 26 | rs297346 | 11 | 16355771 | G | A | 0.638 | -0.016 | 0.002 | 1.81E-11 | 45.19 |
| 27 | rs11605020 | 11 | 126980865 | A | G | 0.514 | 0.014 | 0.002 | 3.59E-09 | 34.81 |
| 28 | rs77607745 | 13 | 69575184 | C | A | 0.190 | 0.016 | 0.003 | 4.95E-08 | 29.75 |
| 29 | rs3843954 | 13 | 58548511 | C | G | 0.292 | 0.014 | 0.003 | 3.82E-08 | 30.23 |
| 30 | rs1782179 | 14 | 41693940 | C | T | 0.727 | 0.015 | 0.003 | 1.50E-08 | 32.08 |
| 31 | rs10144845 | 14 | 75237770 | T | C | 0.683 | 0.016 | 0.002 | 1.05E-10 | 41.73 |
| 32 | rs35755513 | 15 | 64648186 | T | C | 0.074 | 0.024 | 0.004 | 4.12E-08 | 30.10 |
| 33 | rs7175083 | 15 | 78006842 | C | T | 0.522 | -0.015 | 0.002 | 2.21E-10 | 40.30 |
| 34 | rs9930139 | 16 | 7289570 | C | A | 0.588 | 0.014 | 0.002 | 1.19E-08 | 32.52 |
| 35 | rs12938775 | 17 | 2574821 | A | G | 0.503 | -0.013 | 0.002 | 2.49E-08 | 31.06 |
| 36 | rs4632195 | 18 | 50746748 | T | C | 0.513 | 0.016 | 0.002 | 6.63E-12 | 47.13 |
| 37 | rs599550 | 18 | 53252388 | A | G | 0.850 | 0.027 | 0.003 | 3.69E-17 | 70.96 |
| 38 | rs4578918 | 20 | 44721656 | C | T | 0.740 | -0.018 | 0.003 | 1.03E-11 | 46.29 |

| **SNP** | **A1** | **A2** | **EAF** | **irritability** | | | **CAD** | | |
| --- | --- | --- | --- | --- | --- | --- | --- | --- | --- |
|  |  |  |  | **Beta** | **SE** | ***P-*val** | **Beta** | **SE** | ***P-*val** |
| rs10463587 | G | C | 0.219 | 0.008 | 0.001 | 4.70E-09 | 0.010 | 0.008 | 0.220 |
| rs10905638 | C | G | 0.444 | 0.006 | 0.001 | 1.15E-08 | 0.014 | 0.007 | 0.042 |
| rs12612050 | A | G | 0.455 | 0.006 | 0.001 | 3.91E-08 | 0.001 | 0.007 | 0.940 |
| rs16884419 | A | G | 0.236 | 0.009 | 0.001 | 4.44E-11 | 0.008 | 0.008 | 0.300 |
| rs17781446 | T | C | 0.069 | 0.012 | 0.002 | 4.83E-08 | 0.001 | 0.014 | 0.930 |
| rs199497 | C | T | 0.175 | 0.010 | 0.002 | 1.70E-10 | 0.040 | 0.010 | 0.000 |
| rs20551 | G | A | 0.288 | 0.007 | 0.001 | 6.98E-09 | 0.003 | 0.008 | 0.700 |
| rs34340612 | A | G | 0.216 | -0.008 | 0.001 | 6.83E-09 | -0.003 | 0.009 | 0.710 |
| rs343936 | C | T | 0.169 | -0.008 | 0.001 | 3.97E-08 | 0.011 | 0.009 | 0.230 |
| rs3772556 | T | C | 0.706 | -0.007 | 0.001 | 6.47E-09 | -0.004 | 0.008 | 0.620 |
| rs4308307 | T | C | 0.622 | -0.007 | 0.001 | 5.72E-09 | -0.007 | 0.007 | 0.330 |
| rs4903249 | T | C | 0.488 | 0.006 | 0.001 | 1.85E-08 | 0.020 | 0.007 | 0.004 |
| rs58446129 | T | C | 0.146 | 0.010 | 0.002 | 1.08E-09 | 0.036 | 0.010 | 0.000 |
| rs6058104 | A | G | 0.824 | -0.010 | 0.001 | 5.69E-12 | 0.009 | 0.009 | 0.340 |
| rs7181641 | G | A | 0.148 | 0.009 | 0.002 | 2.62E-09 | -0.003 | 0.010 | 0.780 |
| rs7235757 | A | G | 0.311 | 0.008 | 0.001 | 2.13E-11 | 0.003 | 0.008 | 0.650 |
| rs776472 | T | C | 0.566 | -0.007 | 0.001 | 2.97E-09 | 0.002 | 0.007 | 0.790 |
| rs7805419 | C | T | 0.414 | 0.006 | 0.001 | 4.83E-08 | 0.026 | 0.007 | 0.000 |
| rs871852 | C | G | 0.494 | 0.006 | 0.001 | 3.05E-08 | 0.013 | 0.007 | 0.072 |
| rs9390366 | G | T | 0.226 | 0.007 | 0.001 | 4.82E-08 | 0.004 | 0.008 | 0.640 |
| rs999483 | G | T | 0.250 | 0.008 | 0.001 | 2.27E-09 | 0.007 | 0.008 | 0.370 |

Supplementary Table 5: Mendelian randomization analysis of irritability on CAD.

Supplementary Table 6: Mendelian randomization analysis of irritability on MI.

| **SNP** | **A1** | **A2** | **EAF** | **irritability** | | | **MI** | | |
| --- | --- | --- | --- | --- | --- | --- | --- | --- | --- |
|  |  |  |  | **Beta** | **SE** | ***P-*val** | **Beta** | **SE** | ***P-*val** |
| rs10463587 | G | C | 0.217 | 0.008 | 0.001 | 4.70E-09 | 0.010 | 0.009 | 0.292 |
| rs10905638 | C | G | 0.430 | 0.006 | 0.001 | 1.15E-08 | -0.002 | 0.007 | 0.813 |
| rs12612050 | A | G | 0.470 | 0.006 | 0.001 | 3.91E-08 | 0.002 | 0.007 | 0.799 |
| rs16884419 | A | G | 0.250 | 0.009 | 0.001 | 4.44E-11 | 0.003 | 0.009 | 0.740 |
| rs17781446 | T | C | 0.066 | 0.012 | 0.002 | 4.83E-08 | -0.024 | 0.015 | 0.113 |
| rs199497 | C | T | 0.157 | 0.010 | 0.002 | 1.70E-10 | 0.044 | 0.011 | 0.000 |
| rs20551 | G | A | 0.274 | 0.007 | 0.001 | 6.98E-09 | 0.008 | 0.008 | 0.331 |
| rs34340612 | A | G | 0.203 | -0.008 | 0.001 | 6.83E-09 | -0.015 | 0.009 | 0.110 |
| rs343936 | C | T | 0.170 | -0.008 | 0.001 | 3.97E-08 | 0.003 | 0.010 | 0.738 |
| rs3772556 | T | C | 0.702 | -0.007 | 0.001 | 6.47E-09 | -0.011 | 0.008 | 0.188 |
| rs4308307 | T | C | 0.623 | -0.007 | 0.001 | 5.72E-09 | -0.012 | 0.008 | 0.120 |
| rs4903249 | T | C | 0.468 | 0.006 | 0.001 | 1.85E-08 | 0.006 | 0.007 | 0.418 |
| rs58446129 | T | C | 0.165 | 0.010 | 0.002 | 1.08E-09 | 0.006 | 0.010 | 0.530 |
| rs6058104 | A | G | 0.806 | -0.010 | 0.001 | 5.69E-12 | 0.013 | 0.010 | 0.177 |
| rs7181641 | G | A | 0.147 | 0.009 | 0.002 | 2.62E-09 | -0.006 | 0.011 | 0.562 |
| rs7235757 | A | G | 0.323 | 0.008 | 0.001 | 2.13E-11 | -0.003 | 0.008 | 0.746 |
| rs776472 | T | C | 0.552 | -0.007 | 0.001 | 2.97E-09 | 0.000 | 0.008 | 0.995 |
| rs7805419 | C | T | 0.424 | 0.006 | 0.001 | 4.83E-08 | 0.028 | 0.008 | 0.000 |
| rs871852 | C | G | 0.491 | 0.006 | 0.001 | 3.05E-08 | 0.020 | 0.007 | 0.009 |
| rs9390366 | G | T | 0.236 | 0.007 | 0.001 | 4.82E-08 | 0.003 | 0.009 | 0.745 |
| rs999483 | G | T | 0.228 | 0.008 | 0.001 | 2.27E-09 | 0.017 | 0.010 | 0.080 |

Supplementary Table 7: Mendelian randomization analysis of irritability on CA.

| **SNP** | **A1** | **A2** | **EAF** | **irritability** | | | **CA** | | |
| --- | --- | --- | --- | --- | --- | --- | --- | --- | --- |
|  |  |  |  | **Beta** | **SE** | ***P-*val** | **Beta** | **SE** | ***P-*val** |
| rs10463587 | G | C | 0.205 | 0.008 | 0.001 | 4.70E-09 | 0.024 | 0.024 | 0.309 |
| rs10905638 | C | G | 0.421 | 0.006 | 0.001 | 1.15E-08 | 0.020 | 0.019 | 0.311 |
| rs12612050 | A | G | 0.497 | 0.006 | 0.001 | 3.91E-08 | -0.009 | 0.019 | 0.650 |
| rs16884419 | A | G | 0.241 | 0.009 | 0.001 | 4.44E-11 | 0.058 | 0.022 | 0.009 |
| rs17781446 | T | C | 0.101 | 0.012 | 0.002 | 4.83E-08 | 0.005 | 0.032 | 0.871 |
| rs199497 | C | T | 0.105 | 0.010 | 0.002 | 1.70E-10 | 0.039 | 0.032 | 0.226 |
| rs20551 | G | A | 0.315 | 0.007 | 0.001 | 6.98E-09 | 0.015 | 0.021 | 0.463 |
| rs34340612 | A | G | 0.253 | -0.008 | 0.001 | 6.83E-09 | 0.030 | 0.022 | 0.172 |
| rs343936 | C | T | 0.173 | -0.008 | 0.001 | 3.97E-08 | 0.025 | 0.025 | 0.318 |
| rs3772556 | T | C | 0.706 | -0.007 | 0.001 | 6.47E-09 | 0.009 | 0.021 | 0.678 |
| rs4308307 | T | C | 0.671 | -0.007 | 0.001 | 5.72E-09 | -0.019 | 0.020 | 0.355 |
| rs4903249 | T | C | 0.462 | 0.006 | 0.001 | 1.85E-08 | 0.018 | 0.019 | 0.343 |
| rs58446129 | T | C | 0.186 | 0.010 | 0.002 | 1.08E-09 | 0.027 | 0.024 | 0.263 |
| rs6058104 | A | G | 0.887 | -0.010 | 0.001 | 5.69E-12 | 0.031 | 0.030 | 0.305 |
| rs7181641 | G | A | 0.118 | 0.009 | 0.002 | 2.62E-09 | 0.005 | 0.030 | 0.867 |
| rs7235757 | A | G | 0.264 | 0.008 | 0.001 | 2.13E-11 | 0.033 | 0.022 | 0.126 |
| rs776472 | T | C | 0.564 | -0.007 | 0.001 | 2.97E-09 | -0.037 | 0.019 | 0.054 |
| rs7805419 | C | T | 0.340 | 0.006 | 0.001 | 4.83E-08 | 0.012 | 0.020 | 0.548 |
| rs871852 | C | G | 0.457 | 0.006 | 0.001 | 3.05E-08 | 0.032 | 0.019 | 0.091 |
| rs9390366 | G | T | 0.297 | 0.007 | 0.001 | 4.82E-08 | 0.022 | 0.021 | 0.294 |
| rs999483 | G | T | 0.262 | 0.008 | 0.001 | 2.27E-09 | 0.003 | 0.022 | 0.883 |

Supplementary Table 8: Mendelian randomization analysis of irritability on CABG.

| **SNP** | **A1** | **A2** | **EAF** | **irritability** | | | **CABG** | | |
| --- | --- | --- | --- | --- | --- | --- | --- | --- | --- |
|  |  |  |  | **Beta** | **SE** | ***P-*val** | **Beta** | **SE** | ***P-*val** |
| rs10463587 | G | C | 0.204 | 0.008 | 0.001 | 4.70E-09 | -0.002 | 0.028 | 0.940 |
| rs10905638 | C | G | 0.421 | 0.006 | 0.001 | 1.15E-08 | -0.015 | 0.023 | 0.506 |
| rs12612050 | A | G | 0.497 | 0.006 | 0.001 | 3.91E-08 | 0.001 | 0.022 | 0.982 |
| rs16884419 | A | G | 0.241 | 0.009 | 0.001 | 4.44E-11 | 0.005 | 0.026 | 0.844 |
| rs17781446 | T | C | 0.101 | 0.012 | 0.002 | 4.83E-08 | 0.040 | 0.037 | 0.284 |
| rs199497 | C | T | 0.106 | 0.010 | 0.002 | 1.70E-10 | 0.062 | 0.037 | 0.099 |
| rs20551 | G | A | 0.315 | 0.007 | 0.001 | 6.98E-09 | 0.001 | 0.024 | 0.952 |
| rs34340612 | A | G | 0.253 | -0.008 | 0.001 | 6.83E-09 | 0.028 | 0.026 | 0.281 |
| rs343936 | C | T | 0.173 | -0.008 | 0.001 | 3.97E-08 | 0.073 | 0.030 | 0.013 |
| rs3772556 | T | C | 0.706 | -0.007 | 0.001 | 6.47E-09 | 0.030 | 0.025 | 0.218 |
| rs4308307 | T | C | 0.671 | -0.007 | 0.001 | 5.72E-09 | -0.022 | 0.024 | 0.353 |
| rs4903249 | T | C | 0.462 | 0.006 | 0.001 | 1.85E-08 | 0.011 | 0.022 | 0.611 |
| rs58446129 | T | C | 0.185 | 0.010 | 0.002 | 1.08E-09 | -0.016 | 0.029 | 0.564 |
| rs6058104 | A | G | 0.887 | -0.010 | 0.001 | 5.69E-12 | 0.091 | 0.035 | 0.009 |
| rs7181641 | G | A | 0.118 | 0.009 | 0.002 | 2.62E-09 | -0.053 | 0.035 | 0.131 |
| rs7235757 | A | G | 0.263 | 0.008 | 0.001 | 2.13E-11 | -0.013 | 0.025 | 0.616 |
| rs776472 | T | C | 0.564 | -0.007 | 0.001 | 2.97E-09 | -0.027 | 0.023 | 0.239 |
| rs7805419 | C | T | 0.341 | 0.006 | 0.001 | 4.83E-08 | 0.068 | 0.024 | 0.004 |
| rs871852 | C | G | 0.457 | 0.006 | 0.001 | 3.05E-08 | 0.047 | 0.022 | 0.034 |
| rs9390366 | G | T | 0.297 | 0.007 | 0.001 | 4.82E-08 | 0.028 | 0.024 | 0.246 |
| rs999483 | G | T | 0.262 | 0.008 | 0.001 | 2.27E-09 | -0.054 | 0.026 | 0.036 |

Supplementary Table 9: Mendelian randomization analysis of irritability on RE.

| **SNP** | **A1** | **A2** | **EAF** | **irritability** | | | **RE** | | |
| --- | --- | --- | --- | --- | --- | --- | --- | --- | --- |
|  |  |  |  | **Beta** | **SE** | ***P-*val** | **Beta** | **SE** | ***P-*val** |
| rs10463587 | G | C | 0.205 | 0.008 | 0.001 | 4.70E-09 | 0.018 | 0.021 | 0.392 |
| rs10905638 | C | G | 0.421 | 0.006 | 0.001 | 1.15E-08 | 0.009 | 0.017 | 0.600 |
| rs12612050 | A | G | 0.498 | 0.006 | 0.001 | 3.91E-08 | -0.002 | 0.017 | 0.912 |
| rs16884419 | A | G | 0.241 | 0.009 | 0.001 | 4.44E-11 | 0.034 | 0.019 | 0.082 |
| rs17781446 | T | C | 0.101 | 0.012 | 0.002 | 4.83E-08 | 0.015 | 0.028 | 0.580 |
| rs199497 | C | T | 0.106 | 0.010 | 0.002 | 1.70E-10 | 0.047 | 0.028 | 0.094 |
| rs20551 | G | A | 0.315 | 0.007 | 0.001 | 6.98E-09 | 0.012 | 0.018 | 0.490 |
| rs34340612 | A | G | 0.253 | -0.008 | 0.001 | 6.83E-09 | 0.030 | 0.019 | 0.116 |
| rs343936 | C | T | 0.173 | -0.008 | 0.001 | 3.97E-08 | 0.040 | 0.022 | 0.071 |
| rs3772556 | T | C | 0.706 | -0.007 | 0.001 | 6.47E-09 | 0.027 | 0.018 | 0.145 |
| rs4308307 | T | C | 0.671 | -0.007 | 0.001 | 5.72E-09 | -0.021 | 0.018 | 0.247 |
| rs4903249 | T | C | 0.462 | 0.006 | 0.001 | 1.85E-08 | 0.021 | 0.017 | 0.205 |
| rs58446129 | T | C | 0.186 | 0.010 | 0.002 | 1.08E-09 | -0.003 | 0.021 | 0.903 |
| rs6058104 | A | G | 0.887 | -0.010 | 0.001 | 5.69E-12 | 0.046 | 0.026 | 0.081 |
| rs7181641 | G | A | 0.118 | 0.009 | 0.002 | 2.62E-09 | -0.024 | 0.026 | 0.360 |
| rs7235757 | A | G | 0.264 | 0.008 | 0.001 | 2.13E-11 | 0.021 | 0.019 | 0.277 |
| rs776472 | T | C | 0.564 | -0.007 | 0.001 | 2.97E-09 | -0.030 | 0.017 | 0.070 |
| rs7805419 | C | T | 0.341 | 0.006 | 0.001 | 4.83E-08 | 0.047 | 0.018 | 0.007 |
| rs871852 | C | G | 0.457 | 0.006 | 0.001 | 3.05E-08 | 0.035 | 0.017 | 0.037 |
| rs9390366 | G | T | 0.297 | 0.007 | 0.001 | 4.82E-08 | 0.026 | 0.018 | 0.149 |
| rs999483 | G | T | 0.262 | 0.008 | 0.001 | 2.27E-09 | -0.012 | 0.019 | 0.522 |

Supplementary Table 10: Mendelian randomization analysis of irritability on AF.

| **SNP** | **A1** | **A2** | **EAF** | **irritability** | | | **AF** | | |
| --- | --- | --- | --- | --- | --- | --- | --- | --- | --- |
|  |  |  |  | **Beta** | **SE** | ***P-*val** | **Beta** | **SE** | ***P-*val** |
| rs10463587 | G | C | 0.205 | 0.008 | 0.001 | 4.7E-09 | 0.007 | 0.018 | 0.693 |
| rs10905638 | C | G | 0.421 | 0.006 | 0.001 | 1.15E-08 | 0.027 | 0.015 | 0.073 |
| rs12612050 | A | G | 0.498 | 0.006 | 0.001 | 3.91E-08 | -0.009 | 0.015 | 0.542 |
| rs16884419 | A | G | 0.243 | 0.009 | 0.001 | 4.44E-11 | 0.050 | 0.017 | 0.004 |
| rs17781446 | T | C | 0.101 | 0.012 | 0.002 | 4.83E-08 | -0.022 | 0.025 | 0.366 |
| rs199497 | C | T | 0.107 | 0.010 | 0.002 | 1.7E-10 | 0.034 | 0.024 | 0.169 |
| rs20551 | G | A | 0.314 | 0.007 | 0.001 | 6.98E-09 | 0.030 | 0.016 | 0.058 |
| rs34340612 | A | G | 0.253 | -0.008 | 0.001 | 6.83E-09 | 0.016 | 0.017 | 0.353 |
| rs343936 | C | T | 0.172 | -0.008 | 0.001 | 3.97E-08 | 0.044 | 0.020 | 0.026 |
| rs3772556 | T | C | 0.706 | -0.007 | 0.001 | 6.47E-09 | -0.011 | 0.016 | 0.482 |
| rs4308307 | T | C | 0.671 | -0.007 | 0.001 | 5.72E-09 | -0.025 | 0.016 | 0.115 |
| rs4903249 | T | C | 0.462 | 0.006 | 0.001 | 1.85E-08 | 0.008 | 0.015 | 0.617 |
| rs58446129 | T | C | 0.185 | 0.010 | 0.002 | 1.08E-09 | 0.007 | 0.019 | 0.722 |
| rs6058104 | A | G | 0.887 | -0.010 | 0.001 | 5.69E-12 | 0.029 | 0.023 | 0.218 |
| rs7181641 | G | A | 0.117 | 0.009 | 0.002 | 2.62E-09 | 0.044 | 0.023 | 0.059 |
| rs7235757 | A | G | 0.263 | 0.008 | 0.001 | 2.13E-11 | 0.002 | 0.017 | 0.887 |
| rs776472 | T | C | 0.565 | -0.007 | 0.001 | 2.97E-09 | -0.028 | 0.015 | 0.060 |
| rs7805419 | C | T | 0.340 | 0.006 | 0.001 | 4.83E-08 | 0.008 | 0.016 | 0.622 |
| rs871852 | C | G | 0.457 | 0.006 | 0.001 | 3.05E-08 | 0.043 | 0.015 | 0.004 |
| rs9390366 | G | T | 0.298 | 0.007 | 0.001 | 4.82E-08 | 0.017 | 0.016 | 0.297 |
| rs999483 | G | T | 0.262 | 0.008 | 0.001 | 2.27E-09 | 0.009 | 0.017 | 0.617 |

Supplementary Table 11: Mendelian randomization analysis of irritability on HTN.

| **SNP** | **A1** | **A2** | **EAF** | **irritability** | | | **HTN** | | |
| --- | --- | --- | --- | --- | --- | --- | --- | --- | --- |
|  |  |  |  | **Beta** | **SE** | ***P-*val** | **Beta** | **SE** | ***P-*val** |
| rs10463587 | G | C | 0.205 | 0.008 | 0.001 | 4.70E-09 | -0.001 | 0.011 | 0.946 |
| rs10905638 | C | G | 0.421 | 0.006 | 0.001 | 1.15E-08 | 0.003 | 0.009 | 0.737 |
| rs12612050 | A | G | 0.498 | 0.006 | 0.001 | 3.91E-08 | -0.011 | 0.009 | 0.245 |
| rs16884419 | A | G | 0.241 | 0.009 | 0.001 | 4.44E-11 | -0.007 | 0.011 | 0.494 |
| rs17781446 | T | C | 0.101 | 0.012 | 0.002 | 4.83E-08 | -0.021 | 0.015 | 0.171 |
| rs199497 | C | T | 0.106 | 0.010 | 0.002 | 1.70E-10 | -0.023 | 0.015 | 0.136 |
| rs20551 | G | A | 0.315 | 0.007 | 0.001 | 6.98E-09 | 0.011 | 0.010 | 0.255 |
| rs34340612 | A | G | 0.254 | -0.008 | 0.001 | 6.83E-09 | 0.030 | 0.011 | 0.005 |
| rs343936 | C | T | 0.173 | -0.008 | 0.001 | 3.97E-08 | 0.011 | 0.012 | 0.370 |
| rs3772556 | T | C | 0.707 | -0.007 | 0.001 | 6.47E-09 | -0.008 | 0.010 | 0.455 |
| rs4308307 | T | C | 0.671 | -0.007 | 0.001 | 5.72E-09 | -0.016 | 0.010 | 0.099 |
| rs4903249 | T | C | 0.462 | 0.006 | 0.001 | 1.85E-08 | 0.021 | 0.009 | 0.025 |
| rs58446129 | T | C | 0.186 | 0.010 | 0.002 | 1.08E-09 | 0.016 | 0.012 | 0.162 |
| rs6058104 | A | G | 0.887 | -0.010 | 0.001 | 5.69E-12 | 0.011 | 0.014 | 0.444 |
| rs7181641 | G | A | 0.117 | 0.009 | 0.002 | 2.62E-09 | 0.018 | 0.014 | 0.196 |
| rs7235757 | A | G | 0.264 | 0.008 | 0.001 | 2.13E-11 | 0.006 | 0.010 | 0.533 |
| rs776472 | T | C | 0.564 | -0.007 | 0.001 | 2.97E-09 | -0.019 | 0.009 | 0.041 |
| rs7805419 | C | T | 0.341 | 0.006 | 0.001 | 4.83E-08 | 0.033 | 0.010 | 0.001 |
| rs871852 | C | G | 0.457 | 0.006 | 0.001 | 3.05E-08 | 0.015 | 0.009 | 0.109 |
| rs9390366 | G | T | 0.297 | 0.007 | 0.001 | 4.82E-08 | -0.001 | 0.010 | 0.926 |
| rs999483 | G | T | 0.262 | 0.008 | 0.001 | 2.27E-09 | 0.021 | 0.011 | 0.050 |

Supplementary Table 12: Mendelian randomization analysis of irritability on HHD.

| **SNP** | **A1** | **A2** | **EAF** | **irritability** | | | **HHD** | | |
| --- | --- | --- | --- | --- | --- | --- | --- | --- | --- |
|  |  |  |  | **Beta** | **SE** | ***P-*val** | **Beta** | **SE** | ***P-*val** |
| rs10463587 | G | C | 0.205 | 0.008 | 0.001 | 4.70E-09 | 0.049 | 0.031 | 0.115 |
| rs10905638 | C | G | 0.421 | 0.006 | 0.001 | 1.15E-08 | 0.012 | 0.025 | 0.632 |
| rs12612050 | A | G | 0.498 | 0.006 | 0.001 | 3.91E-08 | 0.030 | 0.025 | 0.226 |
| rs16884419 | A | G | 0.242 | 0.009 | 0.001 | 4.44E-11 | 0.006 | 0.029 | 0.834 |
| rs17781446 | T | C | 0.102 | 0.012 | 0.002 | 4.83E-08 | 0.028 | 0.041 | 0.502 |
| rs199497 | C | T | 0.106 | 0.010 | 0.002 | 1.70E-10 | -0.008 | 0.042 | 0.845 |
| rs20551 | G | A | 0.314 | 0.007 | 0.001 | 6.98E-09 | 0.013 | 0.027 | 0.634 |
| rs34340612 | A | G | 0.253 | -0.008 | 0.001 | 6.83E-09 | 0.008 | 0.029 | 0.779 |
| rs343936 | C | T | 0.173 | -0.008 | 0.001 | 3.97E-08 | -0.033 | 0.033 | 0.316 |
| rs3772556 | T | C | 0.707 | -0.007 | 0.001 | 6.47E-09 | 0.044 | 0.027 | 0.107 |
| rs4308307 | T | C | 0.671 | -0.007 | 0.001 | 5.72E-09 | 0.010 | 0.027 | 0.705 |
| rs4903249 | T | C | 0.461 | 0.006 | 0.001 | 1.85E-08 | 0.015 | 0.025 | 0.562 |
| rs58446129 | T | C | 0.185 | 0.010 | 0.002 | 1.08E-09 | 0.051 | 0.032 | 0.109 |
| rs6058104 | A | G | 0.887 | -0.010 | 0.001 | 5.69E-12 | 0.005 | 0.039 | 0.908 |
| rs7181641 | G | A | 0.117 | 0.009 | 0.002 | 2.62E-09 | 0.004 | 0.039 | 0.910 |
| rs7235757 | A | G | 0.264 | 0.008 | 0.001 | 2.13E-11 | 0.026 | 0.028 | 0.353 |
| rs776472 | T | C | 0.565 | -0.007 | 0.001 | 2.97E-09 | -0.071 | 0.025 | 0.005 |
| rs7805419 | C | T | 0.340 | 0.006 | 0.001 | 4.83E-08 | 0.032 | 0.026 | 0.230 |
| rs871852 | C | G | 0.457 | 0.006 | 0.001 | 3.05E-08 | 0.008 | 0.025 | 0.759 |
| rs9390366 | G | T | 0.297 | 0.007 | 0.001 | 4.82E-08 | 0.008 | 0.027 | 0.762 |
| rs999483 | G | T | 0.262 | 0.008 | 0.001 | 2.27E-09 | 0.015 | 0.029 | 0.604 |

Supplementary Table 13: Mendelian randomization analysis of irritability on NIC.

| **SNP** | **A1** | **A2** | **EAF** | **irritability** | | | **NIC** | | |
| --- | --- | --- | --- | --- | --- | --- | --- | --- | --- |
|  |  |  |  | **Beta** | **SE** | ***P-*val** | **Beta** | **SE** | ***P-*val** |
| rs10463587 | G | C | 0.204 | 0.008 | 0.001 | 4.70E-09 | -0.016 | 0.018 | 0.378 |
| rs10905638 | C | G | 0.421 | 0.006 | 0.001 | 1.15E-08 | 0.016 | 0.015 | 0.297 |
| rs12612050 | A | G | 0.497 | 0.006 | 0.001 | 3.91E-08 | -0.017 | 0.015 | 0.238 |
| rs16884419 | A | G | 0.241 | 0.009 | 0.001 | 4.44E-11 | 0.025 | 0.017 | 0.147 |
| rs17781446 | T | C | 0.101 | 0.012 | 0.002 | 4.83E-08 | 0.048 | 0.024 | 0.048 |
| rs199497 | C | T | 0.106 | 0.010 | 0.002 | 1.70E-10 | -0.043 | 0.024 | 0.078 |
| rs20551 | G | A | 0.315 | 0.007 | 0.001 | 6.98E-09 | 0.024 | 0.016 | 0.135 |
| rs34340612 | A | G | 0.253 | -0.008 | 0.001 | 6.83E-09 | -0.005 | 0.017 | 0.784 |
| rs343936 | C | T | 0.173 | -0.008 | 0.001 | 3.97E-08 | -0.014 | 0.019 | 0.469 |
| rs3772556 | T | C | 0.706 | -0.007 | 0.001 | 6.47E-09 | -0.001 | 0.016 | 0.932 |
| rs4308307 | T | C | 0.671 | -0.007 | 0.001 | 5.72E-09 | -0.024 | 0.016 | 0.119 |
| rs4903249 | T | C | 0.462 | 0.006 | 0.001 | 1.85E-08 | 0.008 | 0.015 | 0.575 |
| rs58446129 | T | C | 0.185 | 0.010 | 0.002 | 1.08E-09 | 0.000 | 0.019 | 0.998 |
| rs6058104 | A | G | 0.887 | -0.010 | 0.001 | 5.69E-12 | -0.025 | 0.023 | 0.271 |
| rs7181641 | G | A | 0.118 | 0.009 | 0.002 | 2.62E-09 | 0.035 | 0.023 | 0.121 |
| rs7235757 | A | G | 0.264 | 0.008 | 0.001 | 2.13E-11 | 0.001 | 0.017 | 0.931 |
| rs776472 | T | C | 0.565 | -0.007 | 0.001 | 2.97E-09 | -0.032 | 0.015 | 0.030 |
| rs7805419 | C | T | 0.340 | 0.006 | 0.001 | 4.83E-08 | 0.012 | 0.016 | 0.436 |
| rs871852 | C | G | 0.456 | 0.006 | 0.001 | 3.05E-08 | 0.030 | 0.015 | 0.042 |
| rs9390366 | G | T | 0.297 | 0.007 | 0.001 | 4.82E-08 | 0.024 | 0.016 | 0.140 |
| rs999483 | G | T | 0.262 | 0.008 | 0.001 | 2.27E-09 | 0.011 | 0.017 | 0.533 |

Supplementary Table 14: Mendelian randomization analysis of irritability on HF.

| **SNP** | **A1** | **A2** | **EAF** | **irritability** | | | **HF** | | |
| --- | --- | --- | --- | --- | --- | --- | --- | --- | --- |
|  |  |  |  | **Beta** | **SE** | ***P-*val** | **Beta** | **SE** | ***P-*val** |
| rs10463587 | G | C | 0.219 | 0.008 | 0.001 | 4.70E-09 | 0.001 | 0.011 | 0.956 |
| rs10905638 | C | G | 0.444 | 0.006 | 0.001 | 1.15E-08 | 0.007 | 0.008 | 0.364 |
| rs12612050 | A | G | 0.455 | 0.006 | 0.001 | 3.91E-08 | 0.003 | 0.008 | 0.678 |
| rs16884419 | A | G | 0.236 | 0.009 | 0.001 | 4.44E-11 | 0.010 | 0.009 | 0.292 |
| rs17781446 | T | C | 0.069 | 0.012 | 0.002 | 4.83E-08 | -0.013 | 0.016 | 0.396 |
| rs199497 | C | T | 0.175 | 0.010 | 0.002 | 1.70E-10 | 0.024 | 0.011 | 0.030 |
| rs20551 | G | A | 0.288 | 0.007 | 0.001 | 6.98E-09 | 0.000 | 0.009 | 0.977 |
| rs34340612 | A | G | 0.216 | -0.008 | 0.001 | 6.83E-09 | -0.001 | 0.010 | 0.921 |
| rs343936 | C | T | 0.169 | -0.008 | 0.001 | 3.97E-08 | -0.018 | 0.011 | 0.104 |
| rs3772556 | T | C | 0.706 | -0.007 | 0.001 | 6.47E-09 | 0.006 | 0.009 | 0.522 |
| rs4308307 | T | C | 0.622 | -0.007 | 0.001 | 5.72E-09 | -0.006 | 0.008 | 0.435 |
| rs4903249 | T | C | 0.488 | 0.006 | 0.001 | 1.85E-08 | -0.003 | 0.008 | 0.670 |
| rs58446129 | T | C | 0.146 | 0.010 | 0.002 | 1.08E-09 | -0.013 | 0.011 | 0.243 |
| rs6058104 | A | G | 0.824 | -0.010 | 0.001 | 5.69E-12 | 0.010 | 0.011 | 0.348 |
| rs7181641 | G | A | 0.148 | 0.009 | 0.002 | 2.62E-09 | 0.009 | 0.011 | 0.427 |
| rs7235757 | A | G | 0.311 | 0.008 | 0.001 | 2.13E-11 | 0.019 | 0.009 | 0.030 |
| rs776472 | T | C | 0.566 | -0.007 | 0.001 | 2.97E-09 | -0.012 | 0.008 | 0.143 |
| rs7805419 | C | T | 0.414 | 0.006 | 0.001 | 4.83E-08 | 0.020 | 0.008 | 0.013 |
| rs871852 | C | G | 0.494 | 0.006 | 0.001 | 3.05E-08 | 0.005 | 0.008 | 0.576 |
| rs9390366 | G | T | 0.226 | 0.007 | 0.001 | 4.82E-08 | 0.028 | 0.009 | 0.003 |

Supplementary Table 15: Mendelian randomization analysis of irritability on stroke.

| **SNP** | **A1** | **A2** | **EAF** | **irritability** | | | **stroke** | | |
| --- | --- | --- | --- | --- | --- | --- | --- | --- | --- |
|  |  |  |  | **Beta** | **SE** | ***P-*val** | **Beta** | **SE** | ***P-*val** |
| rs10463587 | G | C | 0.216 | 0.008 | 0.001 | 4.70E-09 | 0.015 | 0.011 | 0.181 |
| rs10905638 | C | G | 0.438 | 0.006 | 0.001 | 1.15E-08 | 0.007 | 0.009 | 0.443 |
| rs12612050 | A | G | 0.471 | 0.006 | 0.001 | 3.91E-08 | 0.010 | 0.009 | 0.261 |
| rs16884419 | A | G | 0.245 | 0.009 | 0.001 | 4.44E-11 | -0.011 | 0.011 | 0.329 |
| rs17781446 | T | C | 0.066 | 0.012 | 0.002 | 4.83E-08 | 0.007 | 0.019 | 0.693 |
| rs199497 | C | T | 0.175 | 0.010 | 0.002 | 1.70E-10 | 0.015 | 0.015 | 0.295 |
| rs20551 | G | A | 0.280 | 0.007 | 0.001 | 6.98E-09 | -0.001 | 0.010 | 0.959 |
| rs34340612 | A | G | 0.220 | -0.008 | 0.001 | 6.83E-09 | 0.000 | 0.011 | 0.990 |
| rs343936 | C | T | 0.163 | -0.008 | 0.001 | 3.97E-08 | -0.030 | 0.013 | 0.025 |
| rs3772556 | T | C | 0.706 | -0.007 | 0.001 | 6.47E-09 | -0.012 | 0.010 | 0.231 |
| rs4308307 | T | C | 0.626 | -0.007 | 0.001 | 5.72E-09 | 0.006 | 0.010 | 0.520 |
| rs4903249 | T | C | 0.503 | 0.006 | 0.001 | 1.85E-08 | 0.020 | 0.009 | 0.030 |
| rs58446129 | T | C | 0.149 | 0.010 | 0.002 | 1.08E-09 | 0.008 | 0.013 | 0.523 |
| rs6058104 | A | G | 0.845 | -0.010 | 0.001 | 5.69E-12 | -0.009 | 0.013 | 0.457 |
| rs7181641 | G | A | 0.144 | 0.009 | 0.002 | 2.62E-09 | 0.012 | 0.012 | 0.310 |
| rs7235757 | A | G | 0.302 | 0.008 | 0.001 | 2.13E-11 | -0.008 | 0.010 | 0.448 |
| rs776472 | T | C | 0.579 | -0.007 | 0.001 | 2.97E-09 | 0.003 | 0.009 | 0.718 |
| rs7805419 | C | T | 0.407 | 0.006 | 0.001 | 4.83E-08 | 0.023 | 0.009 | 0.016 |
| rs871852 | C | G | 0.493 | 0.006 | 0.001 | 3.05E-08 | 0.005 | 0.010 | 0.608 |
| rs9390366 | G | T | 0.221 | 0.007 | 0.001 | 4.82E-08 | 0.007 | 0.011 | 0.543 |
| rs999483 | G | T | 0.246 | 0.008 | 0.001 | 2.27E-09 | -0.006 | 0.014 | 0.687 |

Supplementary Table 16: Mendelian randomization analysis of irritability on IS.

| **SNP** | **A1** | **A2** | **EAF** | **irritability** | | | **IS** | | |
| --- | --- | --- | --- | --- | --- | --- | --- | --- | --- |
|  |  |  |  | **Beta** | **SE** | ***P-*val** | **Beta** | **SE** | ***P-*val** |
| rs10463587 | G | C | 0.217 | 0.008 | 0.001 | 4.70E-09 | 0.019 | 0.012 | 0.115 |
| rs10905638 | C | G | 0.438 | 0.006 | 0.001 | 1.15E-08 | 0.002 | 0.010 | 0.848 |
| rs12612050 | A | G | 0.471 | 0.006 | 0.001 | 3.91E-08 | 0.002 | 0.010 | 0.811 |
| rs16884419 | A | G | 0.245 | 0.009 | 0.001 | 4.44E-11 | -0.016 | 0.012 | 0.182 |
| rs17781446 | T | C | 0.066 | 0.012 | 0.002 | 4.83E-08 | 0.001 | 0.020 | 0.977 |
| rs199497 | C | T | 0.175 | 0.010 | 0.002 | 1.70E-10 | 0.026 | 0.016 | 0.104 |
| rs20551 | G | A | 0.280 | 0.007 | 0.001 | 6.98E-09 | 0.000 | 0.011 | 0.992 |
| rs34340612 | A | G | 0.220 | -0.008 | 0.001 | 6.83E-09 | 0.001 | 0.012 | 0.944 |
| rs343936 | C | T | 0.163 | -0.008 | 0.001 | 3.97E-08 | -0.025 | 0.015 | 0.090 |
| rs3772556 | T | C | 0.707 | -0.007 | 0.001 | 6.47E-09 | -0.014 | 0.011 | 0.210 |
| rs4308307 | T | C | 0.626 | -0.007 | 0.001 | 5.72E-09 | 0.007 | 0.011 | 0.537 |
| rs4903249 | T | C | 0.502 | 0.006 | 0.001 | 1.85E-08 | 0.023 | 0.010 | 0.018 |
| rs58446129 | T | C | 0.149 | 0.010 | 0.002 | 1.08E-09 | -0.001 | 0.014 | 0.968 |
| rs6058104 | A | G | 0.845 | -0.010 | 0.001 | 5.69E-12 | -0.006 | 0.014 | 0.648 |
| rs7181641 | G | A | 0.147 | 0.009 | 0.002 | 2.62E-09 | 0.023 | 0.014 | 0.104 |
| rs7235757 | A | G | 0.302 | 0.008 | 0.001 | 2.13E-11 | -0.009 | 0.011 | 0.398 |
| rs776472 | T | C | 0.578 | -0.007 | 0.001 | 2.97E-09 | -0.001 | 0.010 | 0.902 |
| rs7805419 | C | T | 0.407 | 0.006 | 0.001 | 4.83E-08 | 0.026 | 0.010 | 0.010 |
| rs871852 | C | G | 0.492 | 0.006 | 0.001 | 3.05E-08 | 0.003 | 0.011 | 0.802 |
| rs9390366 | G | T | 0.220 | 0.007 | 0.001 | 4.82E-08 | 0.010 | 0.012 | 0.422 |
| rs999483 | G | T | 0.246 | 0.008 | 0.001 | 2.27E-09 | -0.004 | 0.015 | 0.802 |

Supplementary Table 17: Mendelian randomization analysis of irritability on ISla.

| **SNP** | **A1** | **A2** | **EAF** | **irritability** | | | **ISla** | | |
| --- | --- | --- | --- | --- | --- | --- | --- | --- | --- |
|  |  |  |  | **Beta** | **SE** | ***P-*val** | **Beta** | **SE** | ***P-*val** |
| rs10463587 | G | C | 0.216 | 0.008 | 0.001 | 4.70E-09 | 0.008 | 0.030 | 0.782 |
| rs10905638 | C | G | 0.439 | 0.006 | 0.001 | 1.15E-08 | -0.014 | 0.025 | 0.581 |
| rs12612050 | A | G | 0.468 | 0.006 | 0.001 | 3.91E-08 | 0.029 | 0.025 | 0.242 |
| rs16884419 | A | G | 0.250 | 0.009 | 0.001 | 4.44E-11 | 0.037 | 0.028 | 0.187 |
| rs17781446 | T | C | 0.065 | 0.012 | 0.002 | 4.83E-08 | 0.004 | 0.051 | 0.934 |
| rs199497 | C | T | 0.177 | 0.010 | 0.002 | 1.70E-10 | 0.009 | 0.040 | 0.833 |
| rs20551 | G | A | 0.278 | 0.007 | 0.001 | 6.98E-09 | 0.029 | 0.028 | 0.304 |
| rs34340612 | A | G | 0.221 | -0.008 | 0.001 | 6.83E-09 | -0.025 | 0.030 | 0.414 |
| rs343936 | C | T | 0.163 | -0.008 | 0.001 | 3.97E-08 | -0.059 | 0.036 | 0.107 |
| rs3772556 | T | C | 0.712 | -0.007 | 0.001 | 6.47E-09 | -0.037 | 0.027 | 0.171 |
| rs4308307 | T | C | 0.625 | -0.007 | 0.001 | 5.72E-09 | 0.039 | 0.026 | 0.137 |
| rs4903249 | T | C | 0.500 | 0.006 | 0.001 | 1.85E-08 | 0.052 | 0.024 | 0.032 |
| rs58446129 | T | C | 0.150 | 0.010 | 0.002 | 1.08E-09 | -0.005 | 0.035 | 0.892 |
| rs6058104 | A | G | 0.845 | -0.010 | 0.001 | 5.69E-12 | 0.012 | 0.034 | 0.730 |
| rs7181641 | G | A | 0.149 | 0.009 | 0.002 | 2.62E-09 | 0.115 | 0.035 | 0.001 |
| rs7235757 | A | G | 0.305 | 0.008 | 0.001 | 2.13E-11 | 0.013 | 0.027 | 0.637 |
| rs776472 | T | C | 0.574 | -0.007 | 0.001 | 2.97E-09 | -0.016 | 0.025 | 0.528 |
| rs7805419 | C | T | 0.406 | 0.006 | 0.001 | 4.83E-08 | 0.061 | 0.025 | 0.016 |
| rs871852 | C | G | 0.491 | 0.006 | 0.001 | 3.05E-08 | -0.006 | 0.026 | 0.827 |
| rs9390366 | G | T | 0.224 | 0.007 | 0.001 | 4.82E-08 | -0.001 | 0.030 | 0.972 |
| rs999483 | G | T | 0.245 | 0.008 | 0.001 | 2.27E-09 | 0.021 | 0.038 | 0.583 |

Supplementary Table 18: Mendelian randomization analysis of irritability on ISsv.

| **SNP** | **A1** | **A2** | **EAF** | **irritability** | | | **ISsv** | | |
| --- | --- | --- | --- | --- | --- | --- | --- | --- | --- |
|  |  |  |  | **Beta** | **SE** | ***P-*val** | **Beta** | **SE** | ***P-*val** |
| rs10463587 | G | C | 0.218 | 0.008 | 0.001 | 4.70E-09 | 0.030 | 0.028 | 0.284 |
| rs10905638 | C | G | 0.440 | 0.006 | 0.001 | 1.15E-08 | -0.013 | 0.023 | 0.567 |
| rs12612050 | A | G | 0.468 | 0.006 | 0.001 | 3.91E-08 | 0.007 | 0.023 | 0.753 |
| rs16884419 | A | G | 0.244 | 0.009 | 0.001 | 4.44E-11 | -0.012 | 0.027 | 0.655 |
| rs17781446 | T | C | 0.066 | 0.012 | 0.002 | 4.83E-08 | 0.023 | 0.048 | 0.627 |
| rs199497 | C | T | 0.179 | 0.010 | 0.002 | 1.70E-10 | 0.050 | 0.035 | 0.160 |
| rs20551 | G | A | 0.280 | 0.007 | 0.001 | 6.98E-09 | -0.016 | 0.027 | 0.549 |
| rs34340612 | A | G | 0.218 | -0.008 | 0.001 | 6.83E-09 | 0.046 | 0.028 | 0.102 |
| rs343936 | C | T | 0.164 | -0.008 | 0.001 | 3.97E-08 | 0.030 | 0.032 | 0.347 |
| rs3772556 | T | C | 0.710 | -0.007 | 0.001 | 6.47E-09 | -0.040 | 0.025 | 0.117 |
| rs4308307 | T | C | 0.626 | -0.007 | 0.001 | 5.72E-09 | 0.060 | 0.025 | 0.018 |
| rs4903249 | T | C | 0.501 | 0.006 | 0.001 | 1.85E-08 | 0.008 | 0.023 | 0.712 |
| rs58446129 | T | C | 0.149 | 0.010 | 0.002 | 1.08E-09 | -0.026 | 0.033 | 0.420 |
| rs6058104 | A | G | 0.839 | -0.010 | 0.001 | 5.69E-12 | -0.009 | 0.031 | 0.765 |
| rs7181641 | G | A | 0.149 | 0.009 | 0.002 | 2.62E-09 | 0.008 | 0.034 | 0.813 |
| rs7235757 | A | G | 0.307 | 0.008 | 0.001 | 2.13E-11 | -0.002 | 0.024 | 0.924 |
| rs776472 | T | C | 0.577 | -0.007 | 0.001 | 2.97E-09 | 0.014 | 0.024 | 0.550 |
| rs7805419 | C | T | 0.408 | 0.006 | 0.001 | 4.83E-08 | 0.021 | 0.024 | 0.376 |
| rs871852 | C | G | 0.494 | 0.006 | 0.001 | 3.05E-08 | 0.007 | 0.023 | 0.751 |
| rs9390366 | G | T | 0.221 | 0.007 | 0.001 | 4.82E-08 | 0.004 | 0.028 | 0.887 |
| rs999483 | G | T | 0.243 | 0.008 | 0.001 | 2.27E-09 | -0.060 | 0.034 | 0.080 |

Supplementary Table 19: Mendelian randomization analysis of irritability on ISce.

| **SNP** | **A1** | **A2** | **EAF** | **irritability** | | | **ISce** | | |
| --- | --- | --- | --- | --- | --- | --- | --- | --- | --- |
|  |  |  |  | **Beta** | **SE** | ***P-*val** | **Beta** | **SE** | ***P-*val** |
| rs10463587 | G | C | 0.216 | 0.008 | 0.001 | 4.70E-09 | 0.011 | 0.023 | 0.630 |
| rs10905638 | C | G | 0.439 | 0.006 | 0.001 | 1.15E-08 | -0.018 | 0.019 | 0.359 |
| rs12612050 | A | G | 0.472 | 0.006 | 0.001 | 3.91E-08 | -0.014 | 0.019 | 0.468 |
| rs16884419 | A | G | 0.245 | 0.009 | 0.001 | 4.44E-11 | -0.003 | 0.022 | 0.904 |
| rs17781446 | T | C | 0.066 | 0.012 | 0.002 | 4.83E-08 | 0.043 | 0.040 | 0.280 |
| rs199497 | C | T | 0.177 | 0.010 | 0.002 | 1.70E-10 | 0.057 | 0.030 | 0.061 |
| rs20551 | G | A | 0.279 | 0.007 | 0.001 | 6.98E-09 | 0.000 | 0.022 | 0.995 |
| rs34340612 | A | G | 0.221 | -0.008 | 0.001 | 6.83E-09 | 0.015 | 0.023 | 0.523 |
| rs343936 | C | T | 0.160 | -0.008 | 0.001 | 3.97E-08 | 0.001 | 0.028 | 0.973 |
| rs3772556 | T | C | 0.709 | -0.007 | 0.001 | 6.47E-09 | 0.025 | 0.021 | 0.233 |
| rs4308307 | T | C | 0.627 | -0.007 | 0.001 | 5.72E-09 | -0.029 | 0.020 | 0.142 |
| rs4903249 | T | C | 0.504 | 0.006 | 0.001 | 1.85E-08 | -0.003 | 0.019 | 0.876 |
| rs58446129 | T | C | 0.150 | 0.010 | 0.002 | 1.08E-09 | 0.055 | 0.027 | 0.041 |
| rs6058104 | A | G | 0.847 | -0.010 | 0.001 | 5.69E-12 | -0.030 | 0.027 | 0.257 |
| rs7181641 | G | A | 0.146 | 0.009 | 0.002 | 2.62E-09 | 0.003 | 0.028 | 0.906 |
| rs7235757 | A | G | 0.302 | 0.008 | 0.001 | 2.13E-11 | -0.012 | 0.021 | 0.554 |
| rs776472 | T | C | 0.578 | -0.007 | 0.001 | 2.97E-09 | -0.021 | 0.020 | 0.289 |
| rs7805419 | C | T | 0.404 | 0.006 | 0.001 | 4.83E-08 | 0.015 | 0.019 | 0.434 |
| rs871852 | C | G | 0.492 | 0.006 | 0.001 | 3.05E-08 | -0.006 | 0.020 | 0.769 |
| rs9390366 | G | T | 0.220 | 0.007 | 0.001 | 4.82E-08 | 0.025 | 0.023 | 0.285 |
| rs999483 | G | T | 0.247 | 0.008 | 0.001 | 2.27E-09 | 0.011 | 0.028 | 0.709 |

Supplementary Table 20: Mendelian randomization analysis of irritability on smoking.

| **SNP** | **A1** | **A2** | **EAF** | **irritability** | | | **smoking** | | |
| --- | --- | --- | --- | --- | --- | --- | --- | --- | --- |
|  |  |  |  | **Beta** | **SE** | ***P-*val** | **Beta** | **SE** | ***P-*val** |
| rs10463587 | G | C | 0.219 | 0.008 | 0.001 | 4.70E-09 | 0.010 | 0.004 | 0.025 |
| rs10905638 | C | G | 0.435 | 0.006 | 0.001 | 1.15E-08 | 0.009 | 0.004 | 0.015 |
| rs12612050 | A | G | 0.478 | 0.006 | 0.001 | 3.91E-08 | -0.003 | 0.004 | 0.414 |
| rs16884419 | A | G | 0.248 | 0.009 | 0.001 | 4.44E-11 | -0.003 | 0.004 | 0.542 |
| rs17781446 | T | C | 0.063 | 0.012 | 0.002 | 4.83E-08 | 0.001 | 0.007 | 0.835 |
| rs199497 | C | T | 0.150 | 0.010 | 0.002 | 1.70E-10 | -0.007 | 0.005 | 0.118 |
| rs20551 | G | A | 0.285 | 0.007 | 0.001 | 6.98E-09 | -0.005 | 0.004 | 0.205 |
| rs34340612 | A | G | 0.207 | -0.008 | 0.001 | 6.83E-09 | -0.009 | 0.004 | 0.047 |
| rs343936 | C | T | 0.172 | -0.008 | 0.001 | 3.97E-08 | -0.002 | 0.005 | 0.634 |
| rs3772556 | T | C | 0.728 | -0.007 | 0.001 | 6.47E-09 | -0.010 | 0.004 | 0.013 |
| rs4308307 | T | C | 0.622 | -0.007 | 0.001 | 5.72E-09 | -0.018 | 0.004 | 0.000 |
| rs4903249 | T | C | 0.484 | 0.006 | 0.001 | 1.85E-08 | -0.003 | 0.004 | 0.393 |
| rs58446129 | T | C | 0.164 | 0.010 | 0.002 | 1.08E-09 | 0.004 | 0.005 | 0.429 |
| rs6058104 | A | G | 0.856 | -0.010 | 0.001 | 5.69E-12 | -0.014 | 0.005 | 0.004 |
| rs7181641 | G | A | 0.153 | 0.009 | 0.002 | 2.62E-09 | 0.007 | 0.005 | 0.173 |
| rs7235757 | A | G | 0.327 | 0.008 | 0.001 | 2.13E-11 | -0.002 | 0.004 | 0.660 |
| rs776472 | T | C | 0.565 | -0.007 | 0.001 | 2.97E-09 | -0.010 | 0.004 | 0.008 |
| rs7805419 | C | T | 0.419 | 0.006 | 0.001 | 4.83E-08 | 0.012 | 0.004 | 0.001 |
| rs871852 | C | G | 0.507 | 0.006 | 0.001 | 3.05E-08 | 0.003 | 0.004 | 0.405 |
| rs9390366 | G | T | 0.228 | 0.007 | 0.001 | 4.82E-08 | 0.011 | 0.004 | 0.008 |
| rs999483 | G | T | 0.232 | 0.008 | 0.001 | 2.27E-09 | 0.009 | 0.004 | 0.026 |

Supplementary Table 21: Mendelian randomization analysis of irritability on insomnia.

| **SNP** | **A1** | **A2** | **EAF** | **irritability** | | | **insomnia** | | |
| --- | --- | --- | --- | --- | --- | --- | --- | --- | --- |
|  |  |  |  | **Beta** | **SE** | ***P-*val** | **Beta** | **SE** | ***P-*val** |
| rs10463587 | G | C | 0.218 | 0.008 | 0.001 | 4.70E-09 | 0.015 | 0.011 | 0.199 |
| rs10905638 | C | G | 0.444 | 0.006 | 0.001 | 1.15E-08 | 0.011 | 0.009 | 0.238 |
| rs12612050 | A | G | 0.455 | 0.006 | 0.001 | 3.91E-08 | 0.001 | 0.009 | 0.896 |
| rs16884419 | A | G | 0.237 | 0.009 | 0.001 | 4.44E-11 | 0.030 | 0.011 | 0.006 |
| rs17781446 | T | C | 0.069 | 0.012 | 0.002 | 4.83E-08 | 0.013 | 0.019 | 0.498 |
| rs199497 | C | T | 0.171 | 0.010 | 0.002 | 1.70E-10 | 0.006 | 0.012 | 0.599 |
| rs20551 | G | A | 0.288 | 0.007 | 0.001 | 6.98E-09 | 0.017 | 0.010 | 0.105 |
| rs34340612 | A | G | 0.216 | -0.008 | 0.001 | 6.83E-09 | -0.015 | 0.011 | 0.199 |
| rs343936 | C | T | 0.166 | -0.008 | 0.001 | 3.97E-08 | 0.006 | 0.013 | 0.633 |
| rs3772556 | T | C | 0.707 | -0.007 | 0.001 | 6.47E-09 | 0.001 | 0.010 | 0.893 |
| rs4308307 | T | C | 0.622 | -0.007 | 0.001 | 5.72E-09 | -0.029 | 0.010 | 0.003 |
| rs4903249 | T | C | 0.489 | 0.006 | 0.001 | 1.85E-08 | -0.011 | 0.009 | 0.240 |
| rs58446129 | T | C | 0.147 | 0.010 | 0.002 | 1.08E-09 | 0.014 | 0.013 | 0.275 |
| rs6058104 | A | G | 0.824 | -0.010 | 0.001 | 5.69E-12 | -0.016 | 0.012 | 0.190 |
| rs7181641 | G | A | 0.148 | 0.009 | 0.002 | 2.62E-09 | 0.038 | 0.013 | 0.005 |
| rs7235757 | A | G | 0.314 | 0.008 | 0.001 | 2.13E-11 | 0.011 | 0.010 | 0.266 |
| rs776472 | T | C | 0.566 | -0.007 | 0.001 | 2.97E-09 | -0.022 | 0.009 | 0.021 |
| rs7805419 | C | T | 0.414 | 0.006 | 0.001 | 4.83E-08 | -0.001 | 0.010 | 0.922 |
| rs871852 | C | G | 0.496 | 0.006 | 0.001 | 3.05E-08 | 0.001 | 0.009 | 0.899 |
| rs9390366 | G | T | 0.227 | 0.007 | 0.001 | 4.82E-08 | 0.002 | 0.011 | 0.839 |
| rs999483 | G | T | 0.250 | 0.008 | 0.001 | 2.27E-09 | 0.017 | 0.011 | 0.127 |

Supplementary Table 22: Mendelian randomization analysis of irritability on

depressed affect.

| **SNP** | **A1** | **A2** | **EAF** | **irritability** | | | **depressed affect** | | |
| --- | --- | --- | --- | --- | --- | --- | --- | --- | --- |
|  |  |  |  | **Beta** | **SE** | ***P-*val** | **Beta** | **SE** | ***P-*val** |
| rs10463587 | G | C | 0.217 | 0.008 | 0.001 | 4.70E-09 | 0.013 | 0.003 | 0.000 |
| rs10905638 | C | G | 0.445 | 0.006 | 0.001 | 1.15E-08 | 0.007 | 0.002 | 0.001 |
| rs12612050 | A | G | 0.455 | 0.006 | 0.001 | 3.91E-08 | 0.008 | 0.002 | 0.001 |
| rs16884419 | A | G | 0.237 | 0.009 | 0.001 | 4.44E-11 | 0.008 | 0.003 | 0.002 |
| rs17781446 | T | C | 0.066 | 0.012 | 0.002 | 4.83E-08 | 0.011 | 0.005 | 0.020 |
| rs20551 | G | A | 0.288 | 0.007 | 0.001 | 6.98E-09 | 0.011 | 0.003 | 0.000 |
| rs34340612 | A | G | 0.216 | -0.008 | 0.001 | 6.83E-09 | -0.003 | 0.003 | 0.234 |
| rs343936 | C | T | 0.166 | -0.008 | 0.001 | 3.97E-08 | -0.010 | 0.003 | 0.002 |
| rs3772556 | T | C | 0.709 | -0.007 | 0.001 | 6.47E-09 | -0.011 | 0.003 | 0.000 |
| rs4308307 | T | C | 0.624 | -0.007 | 0.001 | 5.72E-09 | -0.010 | 0.002 | 0.000 |
| rs4903249 | T | C | 0.489 | 0.006 | 0.001 | 1.85E-08 | 0.008 | 0.002 | 0.001 |
| rs58446129 | T | C | 0.146 | 0.010 | 0.002 | 1.08E-09 | 0.017 | 0.003 | 0.000 |
| rs6058104 | A | G | 0.826 | -0.010 | 0.001 | 5.69E-12 | -0.006 | 0.003 | 0.066 |
| rs7181641 | G | A | 0.146 | 0.009 | 0.002 | 2.62E-09 | 0.011 | 0.003 | 0.001 |
| rs7235757 | A | G | 0.310 | 0.008 | 0.001 | 2.13E-11 | 0.012 | 0.003 | 0.000 |
| rs776472 | T | C | 0.569 | -0.007 | 0.001 | 2.97E-09 | -0.005 | 0.002 | 0.027 |
| rs7805419 | C | T | 0.414 | 0.006 | 0.001 | 4.83E-08 | 0.015 | 0.002 | 0.000 |
| rs871852 | C | G | 0.493 | 0.006 | 0.001 | 3.05E-08 | 0.006 | 0.002 | 0.008 |
| rs9390366 | G | T | 0.225 | 0.007 | 0.001 | 4.82E-08 | 0.002 | 0.003 | 0.417 |
| rs999483 | G | T | 0.249 | 0.008 | 0.001 | 2.27E-09 | 0.010 | 0.003 | 0.000 |
|  | G | T | 0.262 | 0.008 | 0.001 | 2.27E-09 | 0.009 | 0.017 | 0.617 |

Supplementary Table 23: Mendelian randomization analysis of smoking on CAD.

| **SNP** | **A1** | **A2** | **EAF** | **smoking** | | | **CAD** | | |
| --- | --- | --- | --- | --- | --- | --- | --- | --- | --- |
|  |  |  |  | **Beta** | **SE** | ***P-*val** | **Beta** | **SE** | ***P-*val** |
| rs10001365 | A | G | 0.405 | -0.025 | 0.004 | 6.65E-12 | -0.012 | 0.007 | 0.110 |
| rs10114490 | A | G | 0.198 | -0.026 | 0.005 | 1.81E-08 | -0.007 | 0.009 | 0.450 |
| rs10260968 | A | G | 0.597 | -0.020 | 0.004 | 1.75E-08 | -0.004 | 0.007 | 0.560 |
| rs10279261 | A | G | 0.619 | -0.021 | 0.004 | 5.00E-09 | 0.014 | 0.007 | 0.062 |
| rs10498846 | T | C | 0.473 | 0.021 | 0.004 | 6.62E-09 | 0.008 | 0.007 | 0.270 |
| rs1050847 | T | C | 0.505 | -0.022 | 0.004 | 1.67E-09 | -0.007 | 0.007 | 0.360 |
| rs10905461 | C | T | 0.718 | -0.024 | 0.004 | 7.35E-09 | -0.004 | 0.008 | 0.580 |
| rs11057005 | G | A | 0.430 | -0.021 | 0.004 | 4.85E-09 | -0.001 | 0.007 | 0.860 |
| rs11078713 | G | A | 0.454 | -0.020 | 0.004 | 2.23E-08 | -0.001 | 0.007 | 0.870 |
| rs1154693 | G | A | 0.856 | 0.033 | 0.005 | 3.12E-11 | 0.014 | 0.010 | 0.150 |
| rs1160685 | G | C | 0.478 | 0.021 | 0.004 | 7.20E-09 | -0.004 | 0.007 | 0.570 |
| rs11712680 | C | A | 0.174 | -0.027 | 0.005 | 3.51E-09 | -0.023 | 0.009 | 0.011 |
| rs117143374 | C | T | 0.120 | 0.029 | 0.005 | 2.76E-08 | 0.022 | 0.010 | 0.033 |
| rs11872397 | A | G | 0.252 | -0.025 | 0.004 | 1.43E-09 | 0.000 | 0.008 | 0.970 |
| rs12025237 | C | A | 0.124 | -0.033 | 0.005 | 6.52E-10 | 0.003 | 0.011 | 0.790 |
| rs12186738 | T | G | 0.154 | -0.033 | 0.005 | 3.42E-11 | -0.016 | 0.010 | 0.100 |
| rs12356821 | C | G | 0.140 | 0.039 | 0.005 | 6.27E-15 | 0.006 | 0.010 | 0.560 |
| rs12441907 | A | C | 0.186 | -0.029 | 0.005 | 1.06E-10 | 0.033 | 0.009 | 0.000 |
| rs12474587 | T | G | 0.404 | 0.028 | 0.004 | 1.25E-14 | 0.005 | 0.007 | 0.510 |
| rs12545053 | G | A | 0.397 | 0.020 | 0.004 | 2.43E-08 | 0.004 | 0.007 | 0.540 |
| rs13030994 | A | G | 0.485 | 0.036 | 0.004 | 3.56E-24 | 0.011 | 0.007 | 0.110 |
| rs13145728 | C | G | 0.358 | -0.023 | 0.004 | 2.14E-10 | -0.012 | 0.007 | 0.100 |
| rs13261666 | T | G | 0.522 | -0.027 | 0.004 | 3.90E-14 | -0.005 | 0.007 | 0.470 |
| rs134529 | C | T | 0.349 | -0.020 | 0.004 | 4.85E-08 | -0.006 | 0.007 | 0.390 |
| rs1385108 | T | C | 0.239 | 0.025 | 0.004 | 3.00E-09 | 0.020 | 0.008 | 0.015 |
| rs1435741 | A | G | 0.425 | 0.029 | 0.004 | 2.64E-16 | -0.001 | 0.007 | 0.930 |
| rs1445649 | C | T | 0.525 | 0.024 | 0.004 | 1.68E-11 | 0.004 | 0.007 | 0.580 |
| rs1555445 | T | A | 0.337 | 0.023 | 0.004 | 3.65E-09 | 0.006 | 0.008 | 0.400 |
| rs1565735 | A | T | 0.212 | -0.038 | 0.004 | 3.42E-17 | -0.008 | 0.009 | 0.350 |
| rs1869243 | C | T | 0.481 | 0.020 | 0.004 | 2.97E-08 | 0.022 | 0.007 | 0.002 |
| rs1899896 | T | C | 0.286 | 0.026 | 0.004 | 1.04E-11 | -0.007 | 0.008 | 0.360 |
| rs1971318 | T | C | 0.141 | 0.029 | 0.005 | 7.06E-09 | -0.024 | 0.010 | 0.010 |
| rs2046850 | T | C | 0.187 | -0.025 | 0.004 | 3.03E-08 | -0.020 | 0.009 | 0.027 |
| rs2107300 | G | C | 0.845 | -0.027 | 0.005 | 3.27E-08 | -0.028 | 0.010 | 0.004 |
| rs2140114 | T | C | 0.518 | -0.023 | 0.004 | 4.70E-10 | -0.018 | 0.007 | 0.009 |
| rs2186122 | T | A | 0.561 | 0.026 | 0.004 | 3.61E-13 | 0.011 | 0.007 | 0.110 |
| rs222449 | T | A | 0.793 | -0.025 | 0.004 | 1.08E-08 | -0.007 | 0.009 | 0.450 |
| rs2378662 | A | G | 0.556 | 0.021 | 0.004 | 4.16E-09 | -0.001 | 0.007 | 0.910 |
| rs240963 | C | T | 0.836 | -0.041 | 0.005 | 2.16E-17 | -0.020 | 0.009 | 0.035 |
| rs2631024 | G | A | 0.737 | -0.023 | 0.004 | 1.18E-08 | 0.010 | 0.008 | 0.190 |
| rs266047 | A | G | 0.529 | -0.031 | 0.004 | 3.36E-16 | -0.009 | 0.007 | 0.210 |
| rs3001723 | A | G | 0.321 | 0.034 | 0.004 | 8.12E-18 | 0.001 | 0.008 | 0.940 |
| rs35702515 | T | G | 0.162 | 0.025 | 0.004 | 2.43E-09 | 0.014 | 0.009 | 0.110 |
| rs3801289 | C | A | 0.351 | -0.022 | 0.004 | 3.74E-09 | -0.007 | 0.007 | 0.320 |
| rs3904512 | A | G | 0.429 | -0.021 | 0.004 | 3.23E-09 | -0.011 | 0.007 | 0.120 |
| rs4044321 | G | A | 0.642 | -0.028 | 0.004 | 6.08E-14 | -0.023 | 0.007 | 0.002 |
| rs4236259 | G | T | 0.499 | -0.025 | 0.004 | 3.35E-12 | 0.000 | 0.007 | 0.990 |
| rs4352629 | T | C | 0.492 | -0.028 | 0.004 | 1.22E-14 | 0.008 | 0.007 | 0.230 |
| rs4523689 | G | A | 0.408 | -0.021 | 0.004 | 1.55E-08 | 0.006 | 0.007 | 0.430 |
| rs4543592 | C | T | 0.468 | 0.022 | 0.004 | 7.46E-10 | 0.011 | 0.007 | 0.110 |
| rs4674993 | G | A | 0.207 | -0.025 | 0.004 | 1.32E-08 | -0.007 | 0.009 | 0.420 |
| rs4781977 | C | T | 0.205 | -0.024 | 0.004 | 4.54E-08 | 0.002 | 0.009 | 0.850 |
| rs4785836 | C | T | 0.398 | -0.020 | 0.004 | 2.26E-08 | 0.013 | 0.007 | 0.082 |
| rs578584 | T | A | 0.605 | 0.029 | 0.004 | 1.50E-15 | 0.008 | 0.007 | 0.270 |
| rs6433897 | C | T | 0.754 | 0.022 | 0.004 | 3.16E-08 | 0.014 | 0.008 | 0.069 |
| rs6508144 | G | C | 0.563 | -0.021 | 0.004 | 7.97E-09 | -0.011 | 0.007 | 0.120 |
| rs6669839 | T | C | 0.204 | 0.026 | 0.004 | 3.36E-09 | 0.003 | 0.008 | 0.730 |
| rs6893752 | G | A | 0.766 | -0.024 | 0.004 | 3.25E-09 | 0.016 | 0.008 | 0.050 |
| rs7197072 | T | C | 0.238 | -0.025 | 0.004 | 2.77E-09 | 0.007 | 0.008 | 0.410 |
| rs7224742 | T | C | 0.595 | -0.021 | 0.004 | 1.43E-08 | -0.010 | 0.007 | 0.160 |
| rs72789632 | T | C | 0.120 | -0.033 | 0.005 | 5.02E-10 | -0.002 | 0.010 | 0.860 |
| rs72896886 | C | G | 0.144 | -0.027 | 0.005 | 2.75E-08 | -0.026 | 0.009 | 0.006 |
| rs7322872 | T | C | 0.782 | -0.026 | 0.004 | 3.58E-09 | 0.001 | 0.008 | 0.910 |
| rs7555507 | T | C | 0.496 | -0.024 | 0.004 | 1.14E-11 | -0.017 | 0.007 | 0.015 |
| rs7585579 | G | C | 0.505 | 0.022 | 0.004 | 1.88E-09 | 0.005 | 0.007 | 0.510 |
| rs76214862 | C | A | 0.202 | -0.025 | 0.005 | 3.99E-08 | 0.005 | 0.009 | 0.540 |
| rs76608582 | A | C | 0.039 | -0.050 | 0.008 | 1.94E-09 | -0.040 | 0.018 | 0.022 |
| rs7921378 | C | G | 0.463 | -0.025 | 0.004 | 8.26E-13 | 0.016 | 0.007 | 0.019 |
| rs7929518 | G | A | 0.765 | 0.024 | 0.004 | 1.56E-08 | 0.011 | 0.008 | 0.180 |
| rs7969559 | G | A | 0.688 | -0.024 | 0.004 | 7.31E-10 | -0.013 | 0.008 | 0.094 |
| rs9401770 | A | G | 0.273 | 0.028 | 0.004 | 3.47E-12 | -0.009 | 0.008 | 0.240 |
| rs9423279 | G | C | 0.641 | -0.021 | 0.004 | 3.21E-08 | 0.008 | 0.008 | 0.290 |
| rs9540729 | T | A | 0.501 | -0.020 | 0.004 | 3.82E-08 | -0.009 | 0.007 | 0.210 |
| rs962625 | G | A | 0.240 | 0.024 | 0.004 | 4.37E-09 | 0.006 | 0.008 | 0.470 |

Supplementary Table 24: Mendelian randomization analysis of smoking on CA.

| **SNP** | **A1** | **A2** | **EAF** | **smoking** | | | **CA** | | |
| --- | --- | --- | --- | --- | --- | --- | --- | --- | --- |
|  |  |  |  | **Beta** | **SE** | ***P-*val** | **Beta** | **SE** | ***P-*val** |
| rs10001365 | A | G | 0.423 | -0.025 | 0.004 | 6.65E-12 | -0.025 | 0.019 | 0.188 |
| rs10260968 | A | G | 0.567 | -0.020 | 0.004 | 1.75E-08 | -0.026 | 0.019 | 0.184 |
| rs10279261 | A | G | 0.641 | -0.021 | 0.004 | 5.00E-09 | -0.026 | 0.020 | 0.196 |
| rs10498846 | T | C | 0.516 | 0.021 | 0.004 | 6.62E-09 | 0.021 | 0.019 | 0.263 |
| rs1050847 | T | C | 0.508 | -0.022 | 0.004 | 1.67E-09 | 0.005 | 0.019 | 0.810 |
| rs10905461 | C | T | 0.840 | -0.024 | 0.004 | 7.35E-09 | 0.022 | 0.026 | 0.392 |
| rs11057005 | G | A | 0.545 | -0.021 | 0.004 | 4.85E-09 | -0.043 | 0.019 | 0.026 |
| rs11078713 | G | A | 0.534 | -0.020 | 0.004 | 2.23E-08 | -0.031 | 0.019 | 0.107 |
| rs1154693 | G | A | 0.784 | 0.033 | 0.005 | 3.12E-11 | -0.031 | 0.023 | 0.176 |
| rs1160685 | G | C | 0.378 | 0.021 | 0.004 | 7.20E-09 | -0.015 | 0.020 | 0.442 |
| rs11712680 | C | A | 0.138 | -0.027 | 0.005 | 3.51E-09 | 0.005 | 0.028 | 0.860 |
| rs117143374 | C | T | 0.106 | 0.029 | 0.005 | 2.76E-08 | 0.010 | 0.031 | 0.753 |
| rs11872397 | A | G | 0.221 | -0.025 | 0.004 | 1.43E-09 | -0.013 | 0.023 | 0.575 |
| rs12025237 | C | A | 0.199 | -0.033 | 0.005 | 6.52E-10 | -0.016 | 0.024 | 0.495 |
| rs12186738 | T | G | 0.144 | -0.033 | 0.005 | 3.42E-11 | -0.031 | 0.027 | 0.255 |
| rs12356821 | C | G | 0.154 | 0.039 | 0.005 | 6.27E-15 | -0.005 | 0.027 | 0.856 |
| rs12441907 | A | C | 0.185 | -0.029 | 0.005 | 1.06E-10 | 0.048 | 0.025 | 0.050 |
| rs12474587 | T | G | 0.347 | 0.028 | 0.004 | 1.25E-14 | 0.022 | 0.020 | 0.272 |
| rs12545053 | G | A | 0.414 | 0.020 | 0.004 | 2.43E-08 | 0.020 | 0.019 | 0.306 |
| rs13030994 | A | G | 0.571 | 0.036 | 0.004 | 3.56E-24 | 0.024 | 0.019 | 0.204 |
| rs13145728 | C | G | 0.363 | -0.023 | 0.004 | 2.14E-10 | -0.010 | 0.020 | 0.601 |
| rs13261666 | T | G | 0.548 | -0.027 | 0.004 | 3.90E-14 | -0.005 | 0.019 | 0.810 |
| rs134529 | C | T | 0.331 | -0.020 | 0.004 | 4.85E-08 | -0.012 | 0.020 | 0.543 |
| rs1385108 | T | C | 0.284 | 0.025 | 0.004 | 3.00E-09 | 0.059 | 0.021 | 0.006 |
| rs1435741 | A | G | 0.375 | 0.029 | 0.004 | 2.64E-16 | 0.046 | 0.020 | 0.018 |
| rs1445649 | C | T | 0.510 | 0.024 | 0.004 | 1.68E-11 | 0.013 | 0.019 | 0.510 |
| rs1555445 | T | A | 0.422 | 0.023 | 0.004 | 3.65E-09 | 0.017 | 0.019 | 0.390 |
| rs1565735 | A | T | 0.147 | -0.038 | 0.004 | 3.42E-17 | -0.036 | 0.027 | 0.182 |
| rs1869243 | C | T | 0.492 | 0.020 | 0.004 | 2.97E-08 | -0.017 | 0.019 | 0.382 |
| rs1899896 | T | C | 0.263 | 0.026 | 0.004 | 1.04E-11 | 0.044 | 0.022 | 0.043 |
| rs1971318 | T | C | 0.118 | 0.029 | 0.005 | 7.06E-09 | -0.019 | 0.030 | 0.527 |
| rs2046850 | T | C | 0.275 | -0.025 | 0.004 | 3.03E-08 | 0.025 | 0.021 | 0.249 |
| rs2050586 | C | G | 0.315 | -0.021 | 0.004 | 3.00E-08 | -0.004 | 0.021 | 0.836 |
| rs2107300 | G | C | 0.852 | -0.027 | 0.005 | 3.27E-08 | -0.042 | 0.027 | 0.117 |
| rs2140114 | T | C | 0.605 | -0.023 | 0.004 | 4.70E-10 | -0.006 | 0.019 | 0.765 |
| rs2186122 | T | A | 0.612 | 0.026 | 0.004 | 3.61E-13 | 0.034 | 0.020 | 0.081 |
| rs222449 | T | A | 0.773 | -0.025 | 0.004 | 1.08E-08 | -0.054 | 0.023 | 0.017 |
| rs2378662 | A | G | 0.564 | 0.021 | 0.004 | 4.16E-09 | -0.011 | 0.019 | 0.575 |
| rs240963 | C | T | 0.801 | -0.041 | 0.005 | 2.16E-17 | -0.004 | 0.024 | 0.875 |
| rs2631024 | G | A | 0.687 | -0.023 | 0.004 | 1.18E-08 | -0.032 | 0.020 | 0.119 |
| rs266047 | A | G | 0.527 | -0.031 | 0.004 | 3.36E-16 | -0.001 | 0.019 | 0.979 |
| rs3001723 | A | G | 0.261 | 0.034 | 0.004 | 8.12E-18 | -0.003 | 0.022 | 0.887 |
| rs35702515 | T | G | 0.167 | 0.025 | 0.004 | 2.43E-09 | -0.043 | 0.026 | 0.090 |
| rs3801289 | C | A | 0.434 | -0.022 | 0.004 | 3.74E-09 | -0.011 | 0.019 | 0.563 |
| rs3904512 | A | G | 0.392 | -0.021 | 0.004 | 3.23E-09 | 0.001 | 0.019 | 0.951 |
| rs4044321 | G | A | 0.661 | -0.028 | 0.004 | 6.08E-14 | -0.038 | 0.020 | 0.056 |
| rs4236259 | G | T | 0.478 | -0.025 | 0.004 | 3.35E-12 | 0.026 | 0.019 | 0.171 |
| rs4352629 | T | C | 0.455 | -0.028 | 0.004 | 1.22E-14 | -0.006 | 0.019 | 0.739 |
| rs4523689 | G | A | 0.438 | -0.021 | 0.004 | 1.55E-08 | -0.008 | 0.019 | 0.677 |
| rs4543592 | C | T | 0.394 | 0.022 | 0.004 | 7.46E-10 | -0.005 | 0.020 | 0.813 |
| rs4674993 | G | A | 0.175 | -0.025 | 0.004 | 1.32E-08 | 0.057 | 0.025 | 0.022 |
| rs4781977 | C | T | 0.092 | -0.024 | 0.004 | 4.54E-08 | 0.039 | 0.033 | 0.238 |
| rs4785836 | C | T | 0.413 | -0.020 | 0.004 | 2.26E-08 | -0.013 | 0.019 | 0.503 |
| rs578584 | T | A | 0.644 | 0.029 | 0.004 | 1.50E-15 | 0.006 | 0.020 | 0.772 |
| rs6433897 | C | T | 0.744 | 0.022 | 0.004 | 3.16E-08 | 0.032 | 0.022 | 0.144 |
| rs6508144 | G | C | 0.569 | -0.021 | 0.004 | 7.97E-09 | 0.000 | 0.019 | 0.992 |
| rs6669839 | T | C | 0.197 | 0.026 | 0.004 | 3.36E-09 | 0.018 | 0.024 | 0.446 |
| rs6893752 | G | A | 0.755 | -0.024 | 0.004 | 3.25E-09 | 0.016 | 0.022 | 0.472 |
| rs7197072 | T | C | 0.352 | -0.025 | 0.004 | 2.77E-09 | -0.017 | 0.020 | 0.401 |
| rs7224742 | T | C | 0.656 | -0.021 | 0.004 | 1.43E-08 | -0.011 | 0.020 | 0.601 |
| rs72789632 | T | C | 0.082 | -0.033 | 0.005 | 5.02E-10 | -0.025 | 0.035 | 0.468 |
| rs72896886 | C | G | 0.164 | -0.027 | 0.005 | 2.75E-08 | 0.018 | 0.026 | 0.492 |
| rs7555507 | T | C | 0.491 | -0.024 | 0.004 | 1.14E-11 | -0.030 | 0.019 | 0.117 |
| rs7585579 | G | C | 0.543 | 0.022 | 0.004 | 1.88E-09 | 0.012 | 0.019 | 0.530 |
| rs76214862 | C | A | 0.235 | -0.025 | 0.005 | 3.99E-08 | -0.022 | 0.023 | 0.323 |
| rs76608582 | A | C | 0.058 | -0.050 | 0.008 | 1.94E-09 | 0.040 | 0.041 | 0.325 |
| rs7921378 | C | G | 0.494 | -0.025 | 0.004 | 8.26E-13 | 0.004 | 0.019 | 0.846 |
| rs7929518 | G | A | 0.743 | 0.024 | 0.004 | 1.56E-08 | 0.014 | 0.022 | 0.529 |
| rs7969559 | G | A | 0.645 | -0.024 | 0.004 | 7.31E-10 | -0.007 | 0.020 | 0.708 |
| rs9401770 | A | G | 0.329 | 0.028 | 0.004 | 3.47E-12 | 0.006 | 0.020 | 0.782 |
| rs9423279 | G | C | 0.600 | -0.021 | 0.004 | 3.21E-08 | -0.037 | 0.020 | 0.062 |
| rs9540729 | T | A | 0.460 | -0.020 | 0.004 | 3.82E-08 | -0.004 | 0.019 | 0.857 |
| rs962625 | G | A | 0.199 | 0.024 | 0.004 | 4.37E-09 | -0.017 | 0.024 | 0.480 |

Supplementary Table 25: Mendelian randomization analysis of smoking on AF.

| **SNP** | **A1** | **A2** | **EAF** | **smoking** | | | **AF** | | |
| --- | --- | --- | --- | --- | --- | --- | --- | --- | --- |
|  |  |  |  | **Beta** | **SE** | ***P-*val** | **Beta** | **SE** | ***P-*val** |
| rs10001365 | A | G | 0.423 | -0.025 | 0.004 | 6.65E-12 | -0.003 | 0.015 | 0.867 |
| rs10260968 | A | G | 0.566 | -0.020 | 0.004 | 1.75E-08 | -0.015 | 0.015 | 0.319 |
| rs10279261 | A | G | 0.640 | -0.021 | 0.004 | 5.00E-09 | -0.005 | 0.015 | 0.726 |
| rs10498846 | T | C | 0.515 | 0.021 | 0.004 | 6.62E-09 | 0.018 | 0.015 | 0.225 |
| rs1050847 | T | C | 0.508 | -0.022 | 0.004 | 1.67E-09 | -0.006 | 0.015 | 0.674 |
| rs10905461 | C | T | 0.840 | -0.024 | 0.004 | 7.35E-09 | 0.008 | 0.020 | 0.694 |
| rs11057005 | G | A | 0.545 | -0.021 | 0.004 | 4.85E-09 | 0.015 | 0.015 | 0.308 |
| rs11078713 | G | A | 0.533 | -0.020 | 0.004 | 2.23E-08 | 0.007 | 0.015 | 0.660 |
| rs1154693 | G | A | 0.784 | 0.033 | 0.005 | 3.12E-11 | -0.025 | 0.018 | 0.168 |
| rs1160685 | G | C | 0.378 | 0.021 | 0.004 | 7.20E-09 | -0.028 | 0.015 | 0.070 |
| rs11712680 | C | A | 0.139 | -0.027 | 0.005 | 3.51E-09 | 0.027 | 0.022 | 0.202 |
| rs117143374 | C | T | 0.106 | 0.029 | 0.005 | 2.76E-08 | 0.064 | 0.024 | 0.008 |
| rs11872397 | A | G | 0.221 | -0.025 | 0.004 | 1.43E-09 | 0.016 | 0.018 | 0.388 |
| rs12025237 | C | A | 0.200 | -0.033 | 0.005 | 6.52E-10 | -0.001 | 0.019 | 0.943 |
| rs12186738 | T | G | 0.143 | -0.033 | 0.005 | 3.42E-11 | 0.002 | 0.021 | 0.913 |
| rs12356821 | C | G | 0.154 | 0.039 | 0.005 | 6.27E-15 | 0.012 | 0.021 | 0.577 |
| rs12441907 | A | C | 0.185 | -0.029 | 0.005 | 1.06E-10 | 0.016 | 0.019 | 0.400 |
| rs12474587 | T | G | 0.348 | 0.028 | 0.004 | 1.25E-14 | -0.002 | 0.016 | 0.903 |
| rs12545053 | G | A | 0.414 | 0.020 | 0.004 | 2.43E-08 | 0.020 | 0.015 | 0.183 |
| rs13030994 | A | G | 0.572 | 0.036 | 0.004 | 3.56E-24 | 0.009 | 0.015 | 0.548 |
| rs13145728 | C | G | 0.365 | -0.023 | 0.004 | 2.14E-10 | 0.006 | 0.015 | 0.711 |
| rs13261666 | T | G | 0.548 | -0.027 | 0.004 | 3.90E-14 | 0.010 | 0.015 | 0.507 |
| rs134529 | C | T | 0.333 | -0.020 | 0.004 | 4.85E-08 | 0.011 | 0.016 | 0.492 |
| rs1385108 | T | C | 0.283 | 0.025 | 0.004 | 3.00E-09 | 0.013 | 0.016 | 0.440 |
| rs1435741 | A | G | 0.375 | 0.029 | 0.004 | 2.64E-16 | 0.003 | 0.015 | 0.843 |
| rs1445649 | C | T | 0.511 | 0.024 | 0.004 | 1.68E-11 | 0.013 | 0.015 | 0.386 |
| rs1555445 | T | A | 0.422 | 0.023 | 0.004 | 3.65E-09 | 0.002 | 0.015 | 0.905 |
| rs1565735 | A | T | 0.148 | -0.038 | 0.004 | 3.42E-17 | -0.013 | 0.021 | 0.549 |
| rs1869243 | C | T | 0.492 | 0.020 | 0.004 | 2.97E-08 | 0.005 | 0.015 | 0.720 |
| rs1899896 | T | C | 0.264 | 0.026 | 0.004 | 1.04E-11 | 0.016 | 0.017 | 0.355 |
| rs1971318 | T | C | 0.118 | 0.029 | 0.005 | 7.06E-09 | -0.001 | 0.023 | 0.981 |
| rs2046850 | T | C | 0.275 | -0.025 | 0.004 | 3.03E-08 | -0.025 | 0.017 | 0.131 |
| rs2050586 | C | G | 0.315 | -0.021 | 0.004 | 3.00E-08 | 0.025 | 0.016 | 0.119 |
| rs2107300 | G | C | 0.852 | -0.027 | 0.005 | 3.27E-08 | -0.016 | 0.021 | 0.435 |
| rs2140114 | T | C | 0.605 | -0.023 | 0.004 | 4.70E-10 | -0.020 | 0.015 | 0.180 |
| rs2186122 | T | A | 0.613 | 0.026 | 0.004 | 3.61E-13 | 0.042 | 0.015 | 0.005 |
| rs222449 | T | A | 0.774 | -0.025 | 0.004 | 1.08E-08 | 0.000 | 0.018 | 0.987 |
| rs2378662 | A | G | 0.565 | 0.021 | 0.004 | 4.16E-09 | 0.015 | 0.015 | 0.321 |
| rs240963 | C | T | 0.801 | -0.041 | 0.005 | 2.16E-17 | -0.010 | 0.019 | 0.591 |
| rs2631024 | G | A | 0.687 | -0.023 | 0.004 | 1.18E-08 | -0.004 | 0.016 | 0.820 |
| rs266047 | A | G | 0.526 | -0.031 | 0.004 | 3.36E-16 | -0.003 | 0.015 | 0.820 |
| rs3001723 | A | G | 0.262 | 0.034 | 0.004 | 8.12E-18 | -0.022 | 0.017 | 0.188 |
| rs35702515 | T | G | 0.168 | 0.025 | 0.004 | 2.43E-09 | -0.019 | 0.020 | 0.337 |
| rs3801289 | C | A | 0.433 | -0.022 | 0.004 | 3.74E-09 | -0.017 | 0.015 | 0.267 |
| rs3904512 | A | G | 0.392 | -0.021 | 0.004 | 3.23E-09 | -0.009 | 0.015 | 0.560 |
| rs4044321 | G | A | 0.662 | -0.028 | 0.004 | 6.08E-14 | 0.018 | 0.016 | 0.255 |
| rs4236259 | G | T | 0.479 | -0.025 | 0.004 | 3.35E-12 | -0.001 | 0.015 | 0.958 |
| rs4352629 | T | C | 0.455 | -0.028 | 0.004 | 1.22E-14 | -0.010 | 0.015 | 0.508 |
| rs4523689 | G | A | 0.438 | -0.021 | 0.004 | 1.55E-08 | 0.008 | 0.015 | 0.579 |
| rs4543592 | C | T | 0.394 | 0.022 | 0.004 | 7.46E-10 | 0.013 | 0.015 | 0.389 |
| rs4674993 | G | A | 0.174 | -0.025 | 0.004 | 1.32E-08 | 0.007 | 0.020 | 0.718 |
| rs4781977 | C | T | 0.092 | -0.024 | 0.004 | 4.54E-08 | -0.003 | 0.026 | 0.901 |
| rs4785836 | C | T | 0.412 | -0.020 | 0.004 | 2.26E-08 | -0.001 | 0.015 | 0.966 |
| rs578584 | T | A | 0.644 | 0.029 | 0.004 | 1.50E-15 | 0.011 | 0.015 | 0.483 |
| rs6433897 | C | T | 0.743 | 0.022 | 0.004 | 3.16E-08 | 0.016 | 0.017 | 0.357 |
| rs6508144 | G | C | 0.570 | -0.021 | 0.004 | 7.97E-09 | -0.004 | 0.015 | 0.777 |
| rs6669839 | T | C | 0.196 | 0.026 | 0.004 | 3.36E-09 | 0.016 | 0.019 | 0.395 |
| rs6893752 | G | A | 0.755 | -0.024 | 0.004 | 3.25E-09 | 0.025 | 0.017 | 0.154 |
| rs7197072 | T | C | 0.352 | -0.025 | 0.004 | 2.77E-09 | 0.037 | 0.016 | 0.018 |
| rs7224742 | T | C | 0.655 | -0.021 | 0.004 | 1.43E-08 | -0.008 | 0.016 | 0.618 |
| rs72789632 | T | C | 0.082 | -0.033 | 0.005 | 5.02E-10 | 0.010 | 0.027 | 0.724 |
| rs72896886 | C | G | 0.164 | -0.027 | 0.005 | 2.75E-08 | -0.039 | 0.020 | 0.056 |
| rs7555507 | T | C | 0.491 | -0.024 | 0.004 | 1.14E-11 | -0.012 | 0.015 | 0.408 |
| rs7585579 | G | C | 0.543 | 0.022 | 0.004 | 1.88E-09 | -0.005 | 0.015 | 0.717 |
| rs76214862 | C | A | 0.235 | -0.025 | 0.005 | 3.99E-08 | -0.010 | 0.018 | 0.569 |
| rs76608582 | A | C | 0.058 | -0.050 | 0.008 | 1.94E-09 | -0.089 | 0.032 | 0.006 |
| rs7921378 | C | G | 0.493 | -0.025 | 0.004 | 8.26E-13 | -0.004 | 0.015 | 0.812 |
| rs7929518 | G | A | 0.743 | 0.024 | 0.004 | 1.56E-08 | 0.019 | 0.017 | 0.260 |
| rs7969559 | G | A | 0.646 | -0.024 | 0.004 | 7.31E-10 | 0.001 | 0.016 | 0.951 |
| rs9401770 | A | G | 0.328 | 0.028 | 0.004 | 3.47E-12 | 0.024 | 0.016 | 0.133 |
| rs9423279 | G | C | 0.600 | -0.021 | 0.004 | 3.21E-08 | 0.015 | 0.015 | 0.313 |
| rs9540729 | T | A | 0.460 | -0.020 | 0.004 | 3.82E-08 | 0.024 | 0.015 | 0.116 |
| rs962625 | G | A | 0.199 | 0.024 | 0.004 | 4.37E-09 | -0.013 | 0.019 | 0.499 |

Supplementary Table 26: Mendelian randomization analysis of smoking on HHD.

| **SNP** | **A1** | **A2** | **EAF** | **smoking** | | | **HHD** | | |
| --- | --- | --- | --- | --- | --- | --- | --- | --- | --- |
|  |  |  |  | **Beta** | **SE** | ***P-*val** | **Beta** | **SE** | ***P-*val** |
| rs10001365 | A | G | 0.422 | -0.025 | 0.004 | 6.65E-12 | -0.012 | 0.025 | 0.628 |
| rs10260968 | A | G | 0.567 | -0.020 | 0.004 | 1.75E-08 | -0.005 | 0.025 | 0.836 |
| rs10279261 | A | G | 0.641 | -0.021 | 0.004 | 5.00E-09 | -0.024 | 0.026 | 0.354 |
| rs10498846 | T | C | 0.516 | 0.021 | 0.004 | 6.62E-09 | -0.026 | 0.025 | 0.302 |
| rs1050847 | T | C | 0.508 | -0.022 | 0.004 | 1.67E-09 | 0.015 | 0.025 | 0.540 |
| rs10905461 | C | T | 0.840 | -0.024 | 0.004 | 7.35E-09 | 0.016 | 0.034 | 0.634 |
| rs11057005 | G | A | 0.545 | -0.021 | 0.004 | 4.85E-09 | -0.005 | 0.025 | 0.835 |
| rs11078713 | G | A | 0.533 | -0.020 | 0.004 | 2.23E-08 | 0.001 | 0.025 | 0.983 |
| rs1154693 | G | A | 0.784 | 0.033 | 0.005 | 3.12E-11 | -0.079 | 0.030 | 0.009 |
| rs1160685 | G | C | 0.379 | 0.021 | 0.004 | 7.20E-09 | -0.009 | 0.026 | 0.743 |
| rs11712680 | C | A | 0.138 | -0.027 | 0.005 | 3.51E-09 | 0.056 | 0.036 | 0.120 |
| rs117143374 | C | T | 0.105 | 0.029 | 0.005 | 2.76E-08 | 0.002 | 0.041 | 0.960 |
| rs11872397 | A | G | 0.220 | -0.025 | 0.004 | 1.43E-09 | -0.027 | 0.030 | 0.379 |
| rs12025237 | C | A | 0.200 | -0.033 | 0.005 | 6.52E-10 | -0.004 | 0.031 | 0.899 |
| rs12186738 | T | G | 0.143 | -0.033 | 0.005 | 3.42E-11 | -0.008 | 0.036 | 0.814 |
| rs12356821 | C | G | 0.152 | 0.039 | 0.005 | 6.27E-15 | -0.014 | 0.035 | 0.681 |
| rs12441907 | A | C | 0.185 | -0.029 | 0.005 | 1.06E-10 | 0.023 | 0.032 | 0.484 |
| rs12474587 | T | G | 0.348 | 0.028 | 0.004 | 1.25E-14 | 0.011 | 0.026 | 0.680 |
| rs12545053 | G | A | 0.414 | 0.020 | 0.004 | 2.43E-08 | 0.039 | 0.025 | 0.127 |
| rs13030994 | A | G | 0.571 | 0.036 | 0.004 | 3.56E-24 | -0.006 | 0.025 | 0.812 |
| rs13145728 | C | G | 0.363 | -0.023 | 0.004 | 2.14E-10 | -0.018 | 0.026 | 0.476 |
| rs13261666 | T | G | 0.548 | -0.027 | 0.004 | 3.90E-14 | 0.023 | 0.025 | 0.365 |
| rs134529 | C | T | 0.332 | -0.020 | 0.004 | 4.85E-08 | 0.005 | 0.027 | 0.840 |
| rs1385108 | T | C | 0.285 | 0.025 | 0.004 | 3.00E-09 | 0.034 | 0.028 | 0.221 |
| rs1435741 | A | G | 0.374 | 0.029 | 0.004 | 2.64E-16 | 0.008 | 0.026 | 0.757 |
| rs1445649 | C | T | 0.511 | 0.024 | 0.004 | 1.68E-11 | 0.030 | 0.025 | 0.224 |
| rs1555445 | T | A | 0.422 | 0.023 | 0.004 | 3.65E-09 | 0.028 | 0.025 | 0.262 |
| rs1565735 | A | T | 0.147 | -0.038 | 0.004 | 3.42E-17 | -0.051 | 0.035 | 0.151 |
| rs1869243 | C | T | 0.492 | 0.020 | 0.004 | 2.97E-08 | 0.000 | 0.025 | 1.000 |
| rs1899896 | T | C | 0.264 | 0.026 | 0.004 | 1.04E-11 | 0.008 | 0.028 | 0.790 |
| rs1971318 | T | C | 0.118 | 0.029 | 0.005 | 7.06E-09 | 0.026 | 0.039 | 0.508 |
| rs2046850 | T | C | 0.275 | -0.025 | 0.004 | 3.03E-08 | -0.060 | 0.028 | 0.030 |
| rs2050586 | C | G | 0.315 | -0.021 | 0.004 | 3.00E-08 | 0.011 | 0.027 | 0.696 |
| rs2107300 | G | C | 0.852 | -0.027 | 0.005 | 3.27E-08 | -0.069 | 0.036 | 0.051 |
| rs2140114 | T | C | 0.605 | -0.023 | 0.004 | 4.70E-10 | 0.005 | 0.026 | 0.858 |
| rs2186122 | T | A | 0.612 | 0.026 | 0.004 | 3.61E-13 | -0.004 | 0.026 | 0.879 |
| rs222449 | T | A | 0.774 | -0.025 | 0.004 | 1.08E-08 | 0.045 | 0.030 | 0.129 |
| rs2378662 | A | G | 0.565 | 0.021 | 0.004 | 4.16E-09 | -0.011 | 0.025 | 0.673 |
| rs240963 | C | T | 0.800 | -0.041 | 0.005 | 2.16E-17 | 0.046 | 0.031 | 0.139 |
| rs2631024 | G | A | 0.688 | -0.023 | 0.004 | 1.18E-08 | 0.001 | 0.027 | 0.959 |
| rs266047 | A | G | 0.526 | -0.031 | 0.004 | 3.36E-16 | -0.027 | 0.025 | 0.283 |
| rs3001723 | A | G | 0.262 | 0.034 | 0.004 | 8.12E-18 | -0.005 | 0.028 | 0.849 |
| rs35702515 | T | G | 0.168 | 0.025 | 0.004 | 2.43E-09 | 0.035 | 0.034 | 0.294 |
| rs3801289 | C | A | 0.433 | -0.022 | 0.004 | 3.74E-09 | -0.013 | 0.025 | 0.618 |
| rs3904512 | A | G | 0.392 | -0.021 | 0.004 | 3.23E-09 | 0.013 | 0.026 | 0.608 |
| rs4044321 | G | A | 0.661 | -0.028 | 0.004 | 6.08E-14 | 0.053 | 0.026 | 0.044 |
| rs4236259 | G | T | 0.478 | -0.025 | 0.004 | 3.35E-12 | 0.036 | 0.025 | 0.154 |
| rs4352629 | T | C | 0.455 | -0.028 | 0.004 | 1.22E-14 | 0.018 | 0.025 | 0.485 |
| rs4523689 | G | A | 0.438 | -0.021 | 0.004 | 1.55E-08 | 0.017 | 0.025 | 0.502 |
| rs4543592 | C | T | 0.394 | 0.022 | 0.004 | 7.46E-10 | -0.047 | 0.026 | 0.067 |
| rs4674993 | G | A | 0.174 | -0.025 | 0.004 | 1.32E-08 | -0.004 | 0.033 | 0.901 |
| rs4781977 | C | T | 0.092 | -0.024 | 0.004 | 4.54E-08 | -0.017 | 0.043 | 0.688 |
| rs4785836 | C | T | 0.413 | -0.020 | 0.004 | 2.26E-08 | 0.024 | 0.025 | 0.350 |
| rs578584 | T | A | 0.644 | 0.029 | 0.004 | 1.50E-15 | -0.025 | 0.026 | 0.328 |
| rs6433897 | C | T | 0.743 | 0.022 | 0.004 | 3.16E-08 | 0.037 | 0.029 | 0.192 |
| rs6508144 | G | C | 0.570 | -0.021 | 0.004 | 7.97E-09 | -0.026 | 0.025 | 0.300 |
| rs6669839 | T | C | 0.197 | 0.026 | 0.004 | 3.36E-09 | 0.016 | 0.032 | 0.620 |
| rs6893752 | G | A | 0.756 | -0.024 | 0.004 | 3.25E-09 | -0.024 | 0.029 | 0.399 |
| rs7197072 | T | C | 0.351 | -0.025 | 0.004 | 2.77E-09 | 0.018 | 0.026 | 0.495 |
| rs7224742 | T | C | 0.655 | -0.021 | 0.004 | 1.43E-08 | -0.005 | 0.026 | 0.851 |
| rs72789632 | T | C | 0.082 | -0.033 | 0.005 | 5.02E-10 | -0.040 | 0.046 | 0.381 |
| rs72896886 | C | G | 0.164 | -0.027 | 0.005 | 2.75E-08 | -0.016 | 0.034 | 0.630 |
| rs7555507 | T | C | 0.491 | -0.024 | 0.004 | 1.14E-11 | -0.008 | 0.025 | 0.753 |
| rs7585579 | G | C | 0.543 | 0.022 | 0.004 | 1.88E-09 | 0.004 | 0.025 | 0.884 |
| rs76214862 | C | A | 0.235 | -0.025 | 0.005 | 3.99E-08 | 0.024 | 0.029 | 0.423 |
| rs76608582 | A | C | 0.058 | -0.050 | 0.008 | 1.94E-09 | -0.037 | 0.053 | 0.488 |
| rs7921378 | C | G | 0.493 | -0.025 | 0.004 | 8.26E-13 | 0.017 | 0.025 | 0.499 |
| rs7929518 | G | A | 0.743 | 0.024 | 0.004 | 1.56E-08 | 0.040 | 0.028 | 0.155 |
| rs7969559 | G | A | 0.646 | -0.024 | 0.004 | 7.31E-10 | -0.087 | 0.026 | 0.001 |
| rs9401770 | A | G | 0.329 | 0.028 | 0.004 | 3.47E-12 | 0.011 | 0.027 | 0.685 |
| rs9423279 | G | C | 0.599 | -0.021 | 0.004 | 3.21E-08 | 0.031 | 0.026 | 0.224 |
| rs9540729 | T | A | 0.460 | -0.020 | 0.004 | 3.82E-08 | -0.011 | 0.025 | 0.670 |
| rs962625 | G | A | 0.200 | 0.024 | 0.004 | 4.37E-09 | -0.029 | 0.031 | 0.358 |

Supplementary Table 27: Mendelian randomization analysis of smoking on NIC.

| **SNP** | **A1** | **A2** | **EAF** | **smoking** | | | **NIC** | | |
| --- | --- | --- | --- | --- | --- | --- | --- | --- | --- |
|  |  |  |  | **Beta** | **SE** | ***P-*val** | **Beta** | **SE** | ***P-*val** |
| rs10001365 | A | G | 0.423 | -0.025 | 0.004 | 6.65E-12 | 0.010 | 0.015 | 0.519 |
| rs10260968 | A | G | 0.567 | -0.020 | 0.004 | 1.75E-08 | -0.010 | 0.015 | 0.486 |
| rs10279261 | A | G | 0.641 | -0.021 | 0.004 | 5.00E-09 | 0.003 | 0.015 | 0.842 |
| rs10498846 | T | C | 0.516 | 0.021 | 0.004 | 6.62E-09 | 0.006 | 0.015 | 0.708 |
| rs1050847 | T | C | 0.508 | -0.022 | 0.004 | 1.67E-09 | 0.003 | 0.015 | 0.844 |
| rs10905461 | C | T | 0.840 | -0.024 | 0.004 | 7.35E-09 | -0.011 | 0.020 | 0.576 |
| rs11057005 | G | A | 0.545 | -0.021 | 0.004 | 4.85E-09 | 0.017 | 0.015 | 0.242 |
| rs11078713 | G | A | 0.534 | -0.020 | 0.004 | 2.23E-08 | 0.023 | 0.015 | 0.125 |
| rs1154693 | G | A | 0.784 | 0.033 | 0.005 | 3.12E-11 | 0.008 | 0.018 | 0.669 |
| rs1160685 | G | C | 0.378 | 0.021 | 0.004 | 7.20E-09 | 0.002 | 0.015 | 0.896 |
| rs11712680 | C | A | 0.138 | -0.027 | 0.005 | 3.51E-09 | -0.023 | 0.021 | 0.283 |
| rs117143374 | C | T | 0.106 | 0.029 | 0.005 | 2.76E-08 | 0.080 | 0.024 | 0.001 |
| rs11872397 | A | G | 0.221 | -0.025 | 0.004 | 1.43E-09 | -0.014 | 0.018 | 0.424 |
| rs12025237 | C | A | 0.199 | -0.033 | 0.005 | 6.52E-10 | -0.014 | 0.018 | 0.455 |
| rs12186738 | T | G | 0.144 | -0.033 | 0.005 | 3.42E-11 | 0.015 | 0.021 | 0.464 |
| rs12356821 | C | G | 0.154 | 0.039 | 0.005 | 6.27E-15 | 0.006 | 0.020 | 0.760 |
| rs12441907 | A | C | 0.185 | -0.029 | 0.005 | 1.06E-10 | 0.006 | 0.019 | 0.753 |
| rs12474587 | T | G | 0.347 | 0.028 | 0.004 | 1.25E-14 | -0.010 | 0.015 | 0.539 |
| rs12545053 | G | A | 0.414 | 0.020 | 0.004 | 2.43E-08 | 0.011 | 0.015 | 0.478 |
| rs13030994 | A | G | 0.571 | 0.036 | 0.004 | 3.56E-24 | -0.008 | 0.015 | 0.579 |
| rs13145728 | C | G | 0.363 | -0.023 | 0.004 | 2.14E-10 | -0.018 | 0.015 | 0.247 |
| rs13261666 | T | G | 0.548 | -0.027 | 0.004 | 3.90E-14 | -0.008 | 0.015 | 0.567 |
| rs134529 | C | T | 0.331 | -0.020 | 0.004 | 4.85E-08 | -0.005 | 0.016 | 0.758 |
| rs1385108 | T | C | 0.284 | 0.025 | 0.004 | 3.00E-09 | -0.004 | 0.016 | 0.831 |
| rs1435741 | A | G | 0.374 | 0.029 | 0.004 | 2.64E-16 | 0.005 | 0.015 | 0.727 |
| rs1445649 | C | T | 0.510 | 0.024 | 0.004 | 1.68E-11 | -0.029 | 0.015 | 0.048 |
| rs1555445 | T | A | 0.422 | 0.023 | 0.004 | 3.65E-09 | 0.001 | 0.015 | 0.956 |
| rs1565735 | A | T | 0.147 | -0.038 | 0.004 | 3.42E-17 | -0.024 | 0.021 | 0.245 |
| rs1869243 | C | T | 0.492 | 0.020 | 0.004 | 2.97E-08 | -0.004 | 0.015 | 0.803 |
| rs1899896 | T | C | 0.263 | 0.026 | 0.004 | 1.04E-11 | 0.027 | 0.017 | 0.109 |
| rs1971318 | T | C | 0.119 | 0.029 | 0.005 | 7.06E-09 | -0.004 | 0.023 | 0.865 |
| rs2046850 | T | C | 0.275 | -0.025 | 0.004 | 3.03E-08 | 0.015 | 0.016 | 0.366 |
| rs2050586 | C | G | 0.315 | -0.021 | 0.004 | 3.00E-08 | 0.023 | 0.016 | 0.144 |
| rs2107300 | G | C | 0.852 | -0.027 | 0.005 | 3.27E-08 | -0.023 | 0.021 | 0.263 |
| rs2140114 | T | C | 0.605 | -0.023 | 0.004 | 4.70E-10 | 0.011 | 0.015 | 0.481 |
| rs2186122 | T | A | 0.612 | 0.026 | 0.004 | 3.61E-13 | 0.010 | 0.015 | 0.491 |
| rs222449 | T | A | 0.774 | -0.025 | 0.004 | 1.08E-08 | -0.024 | 0.018 | 0.172 |
| rs2378662 | A | G | 0.564 | 0.021 | 0.004 | 4.16E-09 | -0.005 | 0.015 | 0.740 |
| rs240963 | C | T | 0.801 | -0.041 | 0.005 | 2.16E-17 | -0.002 | 0.019 | 0.912 |
| rs2631024 | G | A | 0.687 | -0.023 | 0.004 | 1.18E-08 | -0.013 | 0.016 | 0.411 |
| rs266047 | A | G | 0.526 | -0.031 | 0.004 | 3.36E-16 | 0.002 | 0.015 | 0.892 |
| rs3001723 | A | G | 0.261 | 0.034 | 0.004 | 8.12E-18 | 0.008 | 0.017 | 0.633 |
| rs35702515 | T | G | 0.168 | 0.025 | 0.004 | 2.43E-09 | 0.012 | 0.020 | 0.556 |
| rs3801289 | C | A | 0.434 | -0.022 | 0.004 | 3.74E-09 | 0.006 | 0.015 | 0.708 |
| rs3904512 | A | G | 0.393 | -0.021 | 0.004 | 3.23E-09 | 0.004 | 0.015 | 0.804 |
| rs4044321 | G | A | 0.661 | -0.028 | 0.004 | 6.08E-14 | 0.005 | 0.016 | 0.759 |
| rs4236259 | G | T | 0.478 | -0.025 | 0.004 | 3.35E-12 | 0.035 | 0.015 | 0.019 |
| rs4352629 | T | C | 0.455 | -0.028 | 0.004 | 1.22E-14 | -0.023 | 0.015 | 0.113 |
| rs4523689 | G | A | 0.438 | -0.021 | 0.004 | 1.55E-08 | 0.002 | 0.015 | 0.917 |
| rs4543592 | C | T | 0.394 | 0.022 | 0.004 | 7.46E-10 | 0.026 | 0.015 | 0.084 |
| rs4674993 | G | A | 0.175 | -0.025 | 0.004 | 1.32E-08 | -0.024 | 0.019 | 0.207 |
| rs4781977 | C | T | 0.092 | -0.024 | 0.004 | 4.54E-08 | -0.013 | 0.026 | 0.606 |
| rs4785836 | C | T | 0.414 | -0.020 | 0.004 | 2.26E-08 | 0.008 | 0.015 | 0.581 |
| rs578584 | T | A | 0.644 | 0.029 | 0.004 | 1.50E-15 | 0.003 | 0.015 | 0.846 |
| rs6433897 | C | T | 0.744 | 0.022 | 0.004 | 3.16E-08 | 0.021 | 0.017 | 0.205 |
| rs6508144 | G | C | 0.569 | -0.021 | 0.004 | 7.97E-09 | -0.027 | 0.015 | 0.072 |
| rs6669839 | T | C | 0.197 | 0.026 | 0.004 | 3.36E-09 | 0.015 | 0.019 | 0.406 |
| rs6893752 | G | A | 0.755 | -0.024 | 0.004 | 3.25E-09 | 0.002 | 0.017 | 0.903 |
| rs7197072 | T | C | 0.352 | -0.025 | 0.004 | 2.77E-09 | 0.014 | 0.015 | 0.373 |
| rs7224742 | T | C | 0.656 | -0.021 | 0.004 | 1.43E-08 | -0.040 | 0.016 | 0.009 |
| rs72789632 | T | C | 0.082 | -0.033 | 0.005 | 5.02E-10 | 0.006 | 0.027 | 0.836 |
| rs72896886 | C | G | 0.164 | -0.027 | 0.005 | 2.75E-08 | -0.024 | 0.020 | 0.229 |
| rs7555507 | T | C | 0.491 | -0.024 | 0.004 | 1.14E-11 | 0.006 | 0.015 | 0.673 |
| rs7585579 | G | C | 0.543 | 0.022 | 0.004 | 1.88E-09 | 0.013 | 0.015 | 0.372 |
| rs76214862 | C | A | 0.235 | -0.025 | 0.005 | 3.99E-08 | -0.002 | 0.017 | 0.932 |
| rs76608582 | A | C | 0.058 | -0.050 | 0.008 | 1.94E-09 | -0.046 | 0.032 | 0.146 |
| rs7921378 | C | G | 0.494 | -0.025 | 0.004 | 8.26E-13 | 0.007 | 0.015 | 0.639 |
| rs7929518 | G | A | 0.743 | 0.024 | 0.004 | 1.56E-08 | -0.024 | 0.017 | 0.156 |
| rs7969559 | G | A | 0.645 | -0.024 | 0.004 | 7.31E-10 | -0.034 | 0.015 | 0.027 |
| rs9401770 | A | G | 0.329 | 0.028 | 0.004 | 3.47E-12 | 0.013 | 0.016 | 0.390 |
| rs9423279 | G | C | 0.600 | -0.021 | 0.004 | 3.21E-08 | -0.011 | 0.015 | 0.468 |
| rs9540729 | T | A | 0.461 | -0.020 | 0.004 | 3.82E-08 | 0.003 | 0.015 | 0.826 |
| rs962625 | G | A | 0.199 | 0.024 | 0.004 | 4.37E-09 | 0.003 | 0.018 | 0.869 |

Supplementary Table 28: Mendelian randomization analysis of smoking on HF.

| **SNP** | **A1** | **A2** | **EAF** | **smoking** | | | **HF** | | |
| --- | --- | --- | --- | --- | --- | --- | --- | --- | --- |
|  |  |  |  | **Beta** | **SE** | ***P-*val** | **Beta** | **SE** | ***P-*val** |
| rs10001365 | A | G | 0.405 | -0.025 | 0.004 | 6.65E-12 | -0.009 | 0.008 | 0.244 |
| rs10114490 | A | G | 0.198 | -0.026 | 0.005 | 1.81E-08 | -0.011 | 0.010 | 0.263 |
| rs10260968 | A | G | 0.597 | -0.020 | 0.004 | 1.75E-08 | 0.000 | 0.008 | 0.970 |
| rs10279261 | A | G | 0.619 | -0.021 | 0.004 | 5.00E-09 | -0.010 | 0.008 | 0.215 |
| rs10498846 | T | C | 0.473 | 0.021 | 0.004 | 6.62E-09 | 0.013 | 0.008 | 0.095 |
| rs1050847 | T | C | 0.505 | -0.022 | 0.004 | 1.67E-09 | -0.010 | 0.008 | 0.208 |
| rs10905461 | C | T | 0.718 | -0.024 | 0.004 | 7.35E-09 | -0.001 | 0.009 | 0.943 |
| rs11057005 | G | A | 0.430 | -0.021 | 0.004 | 4.85E-09 | -0.003 | 0.008 | 0.701 |
| rs11078713 | G | A | 0.454 | -0.020 | 0.004 | 2.23E-08 | -0.011 | 0.009 | 0.249 |
| rs1154693 | G | A | 0.856 | 0.033 | 0.005 | 3.12E-11 | 0.007 | 0.011 | 0.540 |
| rs1160685 | G | C | 0.478 | 0.021 | 0.004 | 7.20E-09 | 0.004 | 0.008 | 0.622 |
| rs11712680 | C | A | 0.174 | -0.027 | 0.005 | 3.51E-09 | -0.026 | 0.010 | 0.012 |
| rs117143374 | C | T | 0.120 | 0.029 | 0.005 | 2.76E-08 | 0.010 | 0.012 | 0.393 |
| rs11872397 | A | G | 0.252 | -0.025 | 0.004 | 1.43E-09 | -0.004 | 0.009 | 0.658 |
| rs12025237 | C | A | 0.124 | -0.033 | 0.005 | 6.52E-10 | -0.010 | 0.012 | 0.403 |
| rs12186738 | T | G | 0.154 | -0.033 | 0.005 | 3.42E-11 | 0.001 | 0.011 | 0.962 |
| rs12356821 | C | G | 0.140 | 0.039 | 0.005 | 6.27E-15 | -0.004 | 0.011 | 0.719 |
| rs12441907 | A | C | 0.186 | -0.029 | 0.005 | 1.06E-10 | 0.000 | 0.010 | 0.964 |
| rs12474587 | T | G | 0.404 | 0.028 | 0.004 | 1.25E-14 | 0.003 | 0.008 | 0.684 |
| rs12545053 | G | A | 0.397 | 0.020 | 0.004 | 2.43E-08 | -0.011 | 0.008 | 0.188 |
| rs13030994 | A | G | 0.485 | 0.036 | 0.004 | 3.56E-24 | -0.002 | 0.008 | 0.762 |
| rs13145728 | C | G | 0.358 | -0.023 | 0.004 | 2.14E-10 | -0.019 | 0.008 | 0.018 |
| rs13261666 | T | G | 0.522 | -0.027 | 0.004 | 3.90E-14 | 0.001 | 0.008 | 0.917 |
| rs134529 | C | T | 0.349 | -0.020 | 0.004 | 4.85E-08 | -0.016 | 0.008 | 0.047 |
| rs1385108 | T | C | 0.239 | 0.025 | 0.004 | 3.00E-09 | 0.007 | 0.009 | 0.446 |
| rs1435741 | A | G | 0.425 | 0.029 | 0.004 | 2.64E-16 | 0.011 | 0.008 | 0.184 |
| rs1445649 | C | T | 0.525 | 0.024 | 0.004 | 1.68E-11 | 0.014 | 0.008 | 0.076 |
| rs1555445 | T | A | 0.337 | 0.023 | 0.004 | 3.65E-09 | 0.017 | 0.008 | 0.040 |
| rs1565735 | A | T | 0.212 | -0.038 | 0.004 | 3.42E-17 | -0.015 | 0.010 | 0.135 |
| rs1869243 | C | T | 0.481 | 0.020 | 0.004 | 2.97E-08 | -0.006 | 0.008 | 0.438 |
| rs1899896 | T | C | 0.286 | 0.026 | 0.004 | 1.04E-11 | -0.005 | 0.009 | 0.560 |
| rs1971318 | T | C | 0.141 | 0.029 | 0.005 | 7.06E-09 | -0.008 | 0.013 | 0.536 |
| rs2046850 | T | C | 0.187 | -0.025 | 0.004 | 3.03E-08 | -0.010 | 0.010 | 0.302 |
| rs2107300 | G | C | 0.845 | -0.027 | 0.005 | 3.27E-08 | -0.034 | 0.011 | 0.002 |
| rs2140114 | T | C | 0.518 | -0.023 | 0.004 | 4.70E-10 | 0.000 | 0.008 | 0.980 |
| rs2186122 | T | A | 0.561 | 0.026 | 0.004 | 3.61E-13 | 0.001 | 0.008 | 0.861 |
| rs222449 | T | A | 0.793 | -0.025 | 0.004 | 1.08E-08 | 0.009 | 0.010 | 0.358 |
| rs2378662 | A | G | 0.556 | 0.021 | 0.004 | 4.16E-09 | 0.014 | 0.008 | 0.072 |
| rs240963 | C | T | 0.836 | -0.041 | 0.005 | 2.16E-17 | -0.010 | 0.011 | 0.361 |
| rs2631024 | G | A | 0.737 | -0.023 | 0.004 | 1.18E-08 | -0.004 | 0.009 | 0.668 |
| rs266047 | A | G | 0.529 | -0.031 | 0.004 | 3.36E-16 | -0.012 | 0.008 | 0.121 |
| rs3001723 | A | G | 0.321 | 0.034 | 0.004 | 8.12E-18 | 0.003 | 0.009 | 0.718 |
| rs35702515 | T | G | 0.162 | 0.025 | 0.004 | 2.43E-09 | 0.001 | 0.010 | 0.926 |
| rs3801289 | C | A | 0.351 | -0.022 | 0.004 | 3.74E-09 | -0.005 | 0.008 | 0.566 |
| rs3904512 | A | G | 0.429 | -0.021 | 0.004 | 3.23E-09 | -0.003 | 0.008 | 0.707 |
| rs4044321 | G | A | 0.642 | -0.028 | 0.004 | 6.08E-14 | -0.003 | 0.008 | 0.736 |
| rs4236259 | G | T | 0.499 | -0.025 | 0.004 | 3.35E-12 | 0.020 | 0.008 | 0.015 |
| rs4352629 | T | C | 0.492 | -0.028 | 0.004 | 1.22E-14 | -0.010 | 0.008 | 0.221 |
| rs4523689 | G | A | 0.408 | -0.021 | 0.004 | 1.55E-08 | 0.011 | 0.008 | 0.176 |
| rs4543592 | C | T | 0.468 | 0.022 | 0.004 | 7.46E-10 | 0.007 | 0.008 | 0.355 |
| rs4674993 | G | A | 0.207 | -0.025 | 0.004 | 1.32E-08 | 0.007 | 0.010 | 0.491 |
| rs4781977 | C | T | 0.205 | -0.024 | 0.004 | 4.54E-08 | 0.003 | 0.010 | 0.737 |
| rs4785836 | C | T | 0.398 | -0.020 | 0.004 | 2.26E-08 | 0.005 | 0.008 | 0.559 |
| rs578584 | T | A | 0.605 | 0.029 | 0.004 | 1.50E-15 | 0.009 | 0.008 | 0.267 |
| rs6433897 | C | T | 0.754 | 0.022 | 0.004 | 3.16E-08 | 0.010 | 0.009 | 0.288 |
| rs6508144 | G | C | 0.563 | -0.021 | 0.004 | 7.97E-09 | 0.007 | 0.008 | 0.369 |
| rs6669839 | T | C | 0.204 | 0.026 | 0.004 | 3.36E-09 | 0.015 | 0.010 | 0.118 |
| rs6893752 | G | A | 0.766 | -0.024 | 0.004 | 3.25E-09 | -0.013 | 0.009 | 0.158 |
| rs7197072 | T | C | 0.238 | -0.025 | 0.004 | 2.77E-09 | 0.021 | 0.009 | 0.023 |
| rs7224742 | T | C | 0.595 | -0.021 | 0.004 | 1.43E-08 | 0.005 | 0.008 | 0.548 |
| rs72789632 | T | C | 0.120 | -0.033 | 0.005 | 5.02E-10 | 0.018 | 0.012 | 0.143 |
| rs72896886 | C | G | 0.144 | -0.027 | 0.005 | 2.75E-08 | -0.016 | 0.011 | 0.152 |
| rs7322872 | T | C | 0.782 | -0.026 | 0.004 | 3.58E-09 | -0.009 | 0.010 | 0.333 |
| rs7555507 | T | C | 0.496 | -0.024 | 0.004 | 1.14E-11 | -0.009 | 0.008 | 0.230 |
| rs7585579 | G | C | 0.505 | 0.022 | 0.004 | 1.88E-09 | 0.002 | 0.009 | 0.850 |
| rs76214862 | C | A | 0.202 | -0.025 | 0.005 | 3.99E-08 | 0.004 | 0.010 | 0.723 |
| rs76608582 | A | C | 0.039 | -0.050 | 0.008 | 1.94E-09 | -0.008 | 0.021 | 0.719 |
| rs7921378 | C | G | 0.463 | -0.025 | 0.004 | 8.26E-13 | 0.005 | 0.008 | 0.548 |
| rs7929518 | G | A | 0.765 | 0.024 | 0.004 | 1.56E-08 | 0.008 | 0.009 | 0.397 |
| rs7969559 | G | A | 0.688 | -0.024 | 0.004 | 7.31E-10 | -0.008 | 0.009 | 0.347 |
| rs9401770 | A | G | 0.273 | 0.028 | 0.004 | 3.47E-12 | 0.017 | 0.009 | 0.045 |
| rs9423279 | G | C | 0.641 | -0.021 | 0.004 | 3.21E-08 | 0.005 | 0.009 | 0.579 |
| rs9540729 | T | A | 0.501 | -0.020 | 0.004 | 3.82E-08 | -0.004 | 0.008 | 0.584 |
| rs962625 | G | A | 0.240 | 0.024 | 0.004 | 4.37E-09 | 0.031 | 0.009 | 0.001 |

Supplementary Table 29: Mendelian randomization analysis of smoking on ISla.

| **SNP** | **A1** | **A2** | **EAF** | **smoking** | | | ISla | | |
| --- | --- | --- | --- | --- | --- | --- | --- | --- | --- |
|  |  |  |  | **Beta** | **SE** | ***P-*val** | **Beta** | **SE** | ***P-*val** |
| rs10001365 | A | G | 0.398 | -0.025 | 0.004 | 6.65E-12 | 0.014 | 0.026 | 0.599 |
| rs10114490 | A | G | 0.202 | -0.026 | 0.005 | 1.81E-08 | -0.010 | 0.031 | 0.754 |
| rs10260968 | A | G | 0.590 | -0.020 | 0.004 | 1.75E-08 | -0.021 | 0.025 | 0.392 |
| rs10279261 | A | G | 0.607 | -0.021 | 0.004 | 5.00E-09 | 0.003 | 0.025 | 0.920 |
| rs10498846 | T | C | 0.497 | 0.021 | 0.004 | 6.62E-09 | 0.031 | 0.025 | 0.207 |
| rs1050847 | T | C | 0.556 | -0.022 | 0.004 | 1.67E-09 | -0.023 | 0.026 | 0.362 |
| rs10905461 | C | T | 0.747 | -0.024 | 0.004 | 7.35E-09 | 0.010 | 0.029 | 0.720 |
| rs11057005 | G | A | 0.445 | -0.021 | 0.004 | 4.85E-09 | 0.010 | 0.025 | 0.698 |
| rs11078713 | G | A | 0.416 | -0.020 | 0.004 | 2.23E-08 | -0.014 | 0.025 | 0.586 |
| rs1154693 | G | A | 0.848 | 0.033 | 0.005 | 3.12E-11 | 0.019 | 0.034 | 0.585 |
| rs1160685 | G | C | 0.458 | 0.021 | 0.004 | 7.20E-09 | -0.007 | 0.025 | 0.767 |
| rs11712680 | C | A | 0.182 | -0.027 | 0.005 | 3.51E-09 | 0.010 | 0.033 | 0.761 |
| rs117143374 | C | T | 0.131 | 0.029 | 0.005 | 2.76E-08 | -0.023 | 0.037 | 0.542 |
| rs11872397 | A | G | 0.246 | -0.025 | 0.004 | 1.43E-09 | -0.002 | 0.029 | 0.956 |
| rs12025237 | C | A | 0.126 | -0.033 | 0.005 | 6.52E-10 | -0.002 | 0.037 | 0.954 |
| rs12186738 | T | G | 0.157 | -0.033 | 0.005 | 3.42E-11 | -0.021 | 0.034 | 0.538 |
| rs12356821 | C | G | 0.152 | 0.039 | 0.005 | 6.27E-15 | 0.012 | 0.036 | 0.748 |
| rs12441907 | A | C | 0.188 | -0.029 | 0.005 | 1.06E-10 | -0.032 | 0.032 | 0.314 |
| rs12474587 | T | G | 0.422 | 0.028 | 0.004 | 1.25E-14 | -0.009 | 0.025 | 0.722 |
| rs12545053 | G | A | 0.393 | 0.020 | 0.004 | 2.43E-08 | 0.000 | 0.025 | 0.991 |
| rs13030994 | A | G | 0.488 | 0.036 | 0.004 | 3.56E-24 | 0.004 | 0.025 | 0.882 |
| rs13145728 | C | G | 0.376 | -0.023 | 0.004 | 2.14E-10 | 0.011 | 0.026 | 0.679 |
| rs13261666 | T | G | 0.524 | -0.027 | 0.004 | 3.90E-14 | -0.018 | 0.025 | 0.469 |
| rs134529 | C | T | 0.373 | -0.020 | 0.004 | 4.85E-08 | -0.008 | 0.026 | 0.757 |
| rs1385108 | T | C | 0.237 | 0.025 | 0.004 | 3.00E-09 | 0.031 | 0.030 | 0.289 |
| rs1435741 | A | G | 0.443 | 0.029 | 0.004 | 2.64E-16 | 0.058 | 0.025 | 0.019 |
| rs1445649 | C | T | 0.536 | 0.024 | 0.004 | 1.68E-11 | 0.043 | 0.025 | 0.086 |
| rs1555445 | T | A | 0.325 | 0.023 | 0.004 | 3.65E-09 | -0.009 | 0.028 | 0.759 |
| rs1565735 | A | T | 0.202 | -0.038 | 0.004 | 3.42E-17 | -0.011 | 0.031 | 0.713 |
| rs1869243 | C | T | 0.462 | 0.020 | 0.004 | 2.97E-08 | 0.036 | 0.025 | 0.155 |
| rs1899896 | T | C | 0.308 | 0.026 | 0.004 | 1.04E-11 | -0.004 | 0.027 | 0.896 |
| rs1971318 | T | C | 0.151 | 0.029 | 0.005 | 7.06E-09 | -0.001 | 0.035 | 0.971 |
| rs2046850 | T | C | 0.197 | -0.025 | 0.004 | 3.03E-08 | -0.016 | 0.031 | 0.616 |
| rs2107300 | G | C | 0.848 | -0.027 | 0.005 | 3.27E-08 | -0.067 | 0.034 | 0.049 |
| rs2140114 | T | C | 0.524 | -0.023 | 0.004 | 4.70E-10 | 0.002 | 0.025 | 0.946 |
| rs2186122 | T | A | 0.562 | 0.026 | 0.004 | 3.61E-13 | 0.012 | 0.025 | 0.621 |
| rs222449 | T | A | 0.795 | -0.025 | 0.004 | 1.08E-08 | -0.032 | 0.033 | 0.334 |
| rs2378662 | A | G | 0.538 | 0.021 | 0.004 | 4.16E-09 | 0.019 | 0.025 | 0.449 |
| rs240963 | C | T | 0.841 | -0.041 | 0.005 | 2.16E-17 | -0.016 | 0.034 | 0.637 |
| rs2631024 | G | A | 0.731 | -0.023 | 0.004 | 1.18E-08 | 0.055 | 0.028 | 0.045 |
| rs266047 | A | G | 0.530 | -0.031 | 0.004 | 3.36E-16 | -0.050 | 0.025 | 0.045 |
| rs3001723 | A | G | 0.297 | 0.034 | 0.004 | 8.12E-18 | 0.047 | 0.027 | 0.077 |
| rs35702515 | T | G | 0.209 | 0.025 | 0.004 | 2.43E-09 | -0.001 | 0.031 | 0.987 |
| rs3801289 | C | A | 0.361 | -0.022 | 0.004 | 3.74E-09 | -0.018 | 0.028 | 0.531 |
| rs3904512 | A | G | 0.441 | -0.021 | 0.004 | 3.23E-09 | -0.016 | 0.026 | 0.519 |
| rs4044321 | G | A | 0.644 | -0.028 | 0.004 | 6.08E-14 | -0.011 | 0.026 | 0.681 |
| rs4236259 | G | T | 0.490 | -0.025 | 0.004 | 3.35E-12 | 0.009 | 0.027 | 0.730 |
| rs4352629 | T | C | 0.468 | -0.028 | 0.004 | 1.22E-14 | -0.025 | 0.024 | 0.309 |
| rs4523689 | G | A | 0.404 | -0.021 | 0.004 | 1.55E-08 | -0.006 | 0.025 | 0.799 |
| rs4543592 | C | T | 0.478 | 0.022 | 0.004 | 7.46E-10 | -0.035 | 0.027 | 0.199 |
| rs4674993 | G | A | 0.201 | -0.025 | 0.004 | 1.32E-08 | 0.014 | 0.031 | 0.646 |
| rs4781977 | C | T | 0.209 | -0.024 | 0.004 | 4.54E-08 | 0.012 | 0.031 | 0.697 |
| rs4785836 | C | T | 0.387 | -0.020 | 0.004 | 2.26E-08 | -0.011 | 0.026 | 0.656 |
| rs578584 | T | A | 0.582 | 0.029 | 0.004 | 1.50E-15 | -0.002 | 0.026 | 0.926 |
| rs6433897 | C | T | 0.744 | 0.022 | 0.004 | 3.16E-08 | -0.034 | 0.028 | 0.222 |
| rs6508144 | G | C | 0.557 | -0.021 | 0.004 | 7.97E-09 | -0.011 | 0.025 | 0.649 |
| rs6669839 | T | C | 0.202 | 0.026 | 0.004 | 3.36E-09 | 0.027 | 0.030 | 0.379 |
| rs6893752 | G | A | 0.752 | -0.024 | 0.004 | 3.25E-09 | 0.012 | 0.028 | 0.682 |
| rs7197072 | T | C | 0.247 | -0.025 | 0.004 | 2.77E-09 | 0.042 | 0.029 | 0.152 |
| rs7224742 | T | C | 0.607 | -0.021 | 0.004 | 1.43E-08 | 0.009 | 0.026 | 0.729 |
| rs72789632 | T | C | 0.126 | -0.033 | 0.005 | 5.02E-10 | 0.029 | 0.038 | 0.454 |
| rs72896886 | C | G | 0.160 | -0.027 | 0.005 | 2.75E-08 | -0.026 | 0.035 | 0.460 |
| rs7322872 | T | C | 0.778 | -0.026 | 0.004 | 3.58E-09 | 0.022 | 0.034 | 0.518 |
| rs7555507 | T | C | 0.493 | -0.024 | 0.004 | 1.14E-11 | -0.022 | 0.024 | 0.373 |
| rs7585579 | G | C | 0.501 | 0.022 | 0.004 | 1.88E-09 | -0.010 | 0.025 | 0.697 |
| rs76214862 | C | A | 0.196 | -0.025 | 0.005 | 3.99E-08 | 0.038 | 0.031 | 0.223 |
| rs76608582 | A | C | 0.054 | -0.050 | 0.008 | 1.94E-09 | -0.035 | 0.080 | 0.664 |
| rs7921378 | C | G | 0.486 | -0.025 | 0.004 | 8.26E-13 | -0.012 | 0.025 | 0.643 |
| rs7929518 | G | A | 0.772 | 0.024 | 0.004 | 1.56E-08 | 0.013 | 0.029 | 0.661 |
| rs7969559 | G | A | 0.709 | -0.024 | 0.004 | 7.31E-10 | -0.002 | 0.027 | 0.940 |
| rs9401770 | A | G | 0.277 | 0.028 | 0.004 | 3.47E-12 | -0.007 | 0.027 | 0.804 |
| rs9423279 | G | C | 0.638 | -0.021 | 0.004 | 3.21E-08 | 0.043 | 0.032 | 0.187 |
| rs9540729 | T | A | 0.512 | -0.020 | 0.004 | 3.82E-08 | 0.008 | 0.025 | 0.740 |
| rs962625 | G | A | 0.258 | 0.024 | 0.004 | 4.37E-09 | 0.008 | 0.028 | 0.783 |

Supplementary Table 30: Mendelian randomization analysis of insomnia on CAD.

| **SNP** | **A1** | **A2** | **EAF** | **insomnia** | | | **CAD** | | |
| --- | --- | --- | --- | --- | --- | --- | --- | --- | --- |
|  |  |  |  | **Beta** | **SE** | ***P-*val** | **Beta** | **SE** | ***P-*val** |
| rs112383419 | A | G | 0.061 | 0.094 | 0.020 | 2.40E-06 | 0.031 | 0.015 | 0.041 |
| rs113851554 | T | G | 0.056 | 0.178 | 0.020 | 2.14E-18 | 0.010 | 0.016 | 0.520 |
| rs114740697 | G | A | 0.026 | 0.143 | 0.031 | 4.58E-06 | -0.021 | 0.024 | 0.380 |
| rs115780514 | G | T | 0.019 | -0.166 | 0.034 | 1.17E-06 | -0.002 | 0.026 | 0.950 |
| rs116523352 | T | G | 0.019 | -0.174 | 0.036 | 1.76E-06 | -0.030 | 0.028 | 0.280 |
| rs117149835 | C | T | 0.013 | 0.198 | 0.043 | 3.49E-06 | -0.027 | 0.028 | 0.330 |
| rs140007258 | A | T | 0.017 | 0.177 | 0.038 | 2.85E-06 | 0.027 | 0.030 | 0.370 |
| rs145265872 | T | C | 0.046 | -0.107 | 0.023 | 3.02E-06 | -0.016 | 0.017 | 0.340 |
| rs147256205 | A | C | 0.014 | 0.188 | 0.041 | 4.63E-06 | -0.025 | 0.031 | 0.420 |
| rs149251474 | G | C | 0.022 | 0.158 | 0.034 | 2.77E-06 | 0.016 | 0.027 | 0.560 |
| rs1547668 | G | A | 0.796 | -0.054 | 0.012 | 3.61E-06 | -0.020 | 0.009 | 0.020 |
| rs1552291 | G | A | 0.629 | 0.047 | 0.010 | 1.93E-06 | -0.004 | 0.007 | 0.620 |
| rs208827 | G | C | 0.636 | 0.051 | 0.010 | 2.24E-07 | 0.007 | 0.007 | 0.370 |
| rs2206301 | C | T | 0.625 | -0.050 | 0.010 | 3.22E-07 | -0.007 | 0.007 | 0.370 |
| rs2673609 | G | C | 0.360 | 0.045 | 0.010 | 4.98E-06 | 0.004 | 0.007 | 0.580 |
| rs3923386 | T | C | 0.521 | -0.044 | 0.009 | 2.93E-06 | 0.004 | 0.007 | 0.600 |
| rs6790885 | A | G | 0.503 | -0.044 | 0.010 | 3.59E-06 | 0.003 | 0.007 | 0.650 |
| rs72802225 | G | A | 0.014 | 0.204 | 0.043 | 1.89E-06 | 0.031 | 0.033 | 0.350 |
| rs7332332 | G | A | 0.497 | -0.044 | 0.009 | 3.52E-06 | -0.021 | 0.007 | 0.003 |
| rs738475 | G | C | 0.259 | 0.051 | 0.011 | 2.54E-06 | -0.001 | 0.008 | 0.940 |
| rs79278977 | T | C | 0.014 | -0.194 | 0.042 | 3.53E-06 | -0.034 | 0.032 | 0.280 |
| rs865199 | G | A | 0.932 | 0.086 | 0.019 | 4.08E-06 | -0.001 | 0.013 | 0.960 |

Supplementary Table 31: Mendelian randomization analysis of insomnia on CA.

| **SNP** | **A1** | **A2** | **EAF** | **insomnia** | | | **CA** | | |
| --- | --- | --- | --- | --- | --- | --- | --- | --- | --- |
|  |  |  |  | **Beta** | **SE** | ***P-*val** | **Beta** | **SE** | ***P-*val** |
| rs112383419 | A | G | 0.038 | 0.094 | 0.020 | 2.40E-06 | -0.032 | 0.051 | 0.534 |
| rs113851554 | T | G | 0.071 | 0.178 | 0.020 | 2.14E-18 | -0.019 | 0.037 | 0.600 |
| rs114740697 | G | A | 0.014 | 0.143 | 0.031 | 4.58E-06 | -0.027 | 0.082 | 0.740 |
| rs115780514 | G | T | 0.035 | -0.166 | 0.034 | 1.17E-06 | -0.001 | 0.051 | 0.991 |
| rs116523352 | T | G | 0.001 | -0.174 | 0.036 | 1.76E-06 | -0.267 | 0.431 | 0.535 |
| rs140007258 | A | T | 0.012 | 0.177 | 0.038 | 2.85E-06 | 0.021 | 0.087 | 0.809 |
| rs145265872 | T | C | 0.044 | -0.107 | 0.023 | 3.02E-06 | -0.039 | 0.046 | 0.406 |
| rs147256205 | A | C | 0.020 | 0.188 | 0.041 | 4.63E-06 | -0.083 | 0.068 | 0.224 |
| rs149251474 | G | C | 0.017 | 0.158 | 0.034 | 2.77E-06 | -0.025 | 0.076 | 0.739 |
| rs1547668 | G | A | 0.857 | -0.054 | 0.012 | 3.61E-06 | -0.026 | 0.028 | 0.341 |
| rs1552291 | G | A | 0.651 | 0.047 | 0.010 | 1.93E-06 | 0.002 | 0.020 | 0.940 |
| rs1994140 | G | A | 0.605 | -0.046 | 0.009 | 9.99E-07 | 0.003 | 0.023 | 0.912 |
| rs208827 | G | C | 0.642 | 0.051 | 0.010 | 2.24E-07 | 0.023 | 0.020 | 0.244 |
| rs2206301 | C | T | 0.615 | -0.050 | 0.010 | 3.22E-07 | -0.014 | 0.020 | 0.476 |
| rs2673609 | G | C | 0.337 | 0.045 | 0.010 | 4.98E-06 | 0.023 | 0.020 | 0.260 |
| rs3923386 | T | C | 0.547 | -0.044 | 0.009 | 2.93E-06 | -0.011 | 0.019 | 0.582 |
| rs6790885 | A | G | 0.477 | -0.044 | 0.010 | 3.59E-06 | 0.020 | 0.019 | 0.299 |
| rs72802225 | G | A | 0.010 | 0.204 | 0.043 | 1.89E-06 | -0.034 | 0.095 | 0.719 |
| rs7332332 | G | A | 0.529 | -0.044 | 0.009 | 3.52E-06 | 0.036 | 0.019 | 0.061 |
| rs738475 | G | C | 0.273 | 0.051 | 0.011 | 2.54E-06 | -0.015 | 0.022 | 0.474 |
| rs79278977 | T | C | 0.007 | -0.194 | 0.042 | 3.53E-06 | -0.294 | 0.120 | 0.014 |
| rs865199 | G | A | 0.881 | 0.086 | 0.019 | 4.08E-06 | 0.014 | 0.029 | 0.639 |

Supplementary Table 32: Mendelian randomization analysis of insomnia on AF.

| **SNP** | **A1** | **A2** | **EAF** | **insomnia** | | | **AF** | | |
| --- | --- | --- | --- | --- | --- | --- | --- | --- | --- |
|  |  |  |  | **Beta** | **SE** | ***P-*val** | **Beta** | **SE** | ***P-*val** |
| rs112383419 | A | G | 0.038 | 0.094 | 0.020 | 2.40E-06 | 0.083 | 0.039 | 0.035 |
| rs113851554 | T | G | 0.071 | 0.178 | 0.020 | 2.14E-18 | -0.011 | 0.029 | 0.698 |
| rs114740697 | G | A | 0.014 | 0.143 | 0.031 | 4.58E-06 | 0.098 | 0.063 | 0.117 |
| rs115780514 | G | T | 0.034 | -0.166 | 0.034 | 1.17E-06 | -0.001 | 0.041 | 0.974 |
| rs116523352 | T | G | 0.001 | -0.174 | 0.036 | 1.76E-06 | -0.104 | 0.337 | 0.758 |
| rs140007258 | A | T | 0.012 | 0.177 | 0.038 | 2.85E-06 | 0.106 | 0.069 | 0.122 |
| rs145265872 | T | C | 0.044 | -0.107 | 0.023 | 3.02E-06 | -0.075 | 0.036 | 0.038 |
| rs147256205 | A | C | 0.020 | 0.188 | 0.041 | 4.63E-06 | -0.039 | 0.052 | 0.459 |
| rs149251474 | G | C | 0.017 | 0.158 | 0.034 | 2.77E-06 | 0.026 | 0.059 | 0.660 |
| rs1547668 | G | A | 0.857 | -0.054 | 0.012 | 3.61E-06 | 0.014 | 0.021 | 0.507 |
| rs1552291 | G | A | 0.650 | 0.047 | 0.010 | 1.93E-06 | -0.027 | 0.016 | 0.089 |
| rs1994140 | G | A | 0.604 | -0.046 | 0.009 | 9.99E-07 | -0.010 | 0.018 | 0.559 |
| rs208827 | G | C | 0.641 | 0.051 | 0.010 | 2.24E-07 | 0.004 | 0.016 | 0.816 |
| rs2206301 | C | T | 0.616 | -0.050 | 0.010 | 3.22E-07 | 0.022 | 0.015 | 0.160 |
| rs2673609 | G | C | 0.337 | 0.045 | 0.010 | 4.98E-06 | -0.014 | 0.016 | 0.370 |
| rs3923386 | T | C | 0.547 | -0.044 | 0.009 | 2.93E-06 | -0.032 | 0.015 | 0.031 |
| rs6790885 | A | G | 0.478 | -0.044 | 0.010 | 3.59E-06 | 0.016 | 0.015 | 0.283 |
| rs72802225 | G | A | 0.010 | 0.204 | 0.043 | 1.89E-06 | 0.072 | 0.075 | 0.337 |
| rs7332332 | G | A | 0.529 | -0.044 | 0.009 | 3.52E-06 | 0.017 | 0.015 | 0.259 |
| rs738475 | G | C | 0.272 | 0.051 | 0.011 | 2.54E-06 | 0.020 | 0.017 | 0.235 |
| rs79278977 | T | C | 0.007 | -0.194 | 0.042 | 3.53E-06 | 0.130 | 0.095 | 0.170 |
| rs865199 | G | A | 0.881 | 0.086 | 0.019 | 4.08E-06 | -0.027 | 0.023 | 0.244 |

Supplementary Table 33: Mendelian randomization analysis of insomnia on HHD.

| **SNP** | **A1** | **A2** | **EAF** | **insomnia** | | | **HHD** | | |
| --- | --- | --- | --- | --- | --- | --- | --- | --- | --- |
|  |  |  |  | **Beta** | **SE** | ***P-*val** | **Beta** | **SE** | ***P-*val** |
| rs112383419 | A | G | 0.038 | 0.094 | 0.020 | 2.40E-06 | 0.013 | 0.067 | 0.846 |
| rs113851554 | T | G | 0.071 | 0.178 | 0.020 | 2.14E-18 | 0.001 | 0.048 | 0.990 |
| rs114740697 | G | A | 0.014 | 0.143 | 0.031 | 4.58E-06 | 0.158 | 0.108 | 0.144 |
| rs115780514 | G | T | 0.034 | -0.166 | 0.034 | 1.17E-06 | 0.110 | 0.068 | 0.107 |
| rs116523352 | T | G | 0.001 | -0.174 | 0.036 | 1.76E-06 | -0.205 | 0.610 | 0.737 |
| rs140007258 | A | T | 0.012 | 0.177 | 0.038 | 2.85E-06 | 0.172 | 0.115 | 0.133 |
| rs145265872 | T | C | 0.045 | -0.107 | 0.023 | 3.02E-06 | 0.087 | 0.060 | 0.147 |
| rs147256205 | A | C | 0.020 | 0.188 | 0.041 | 4.63E-06 | -0.056 | 0.089 | 0.528 |
| rs149251474 | G | C | 0.017 | 0.158 | 0.034 | 2.77E-06 | 0.156 | 0.099 | 0.113 |
| rs1547668 | G | A | 0.857 | -0.054 | 0.012 | 3.61E-06 | -0.018 | 0.036 | 0.608 |
| rs1552291 | G | A | 0.651 | 0.047 | 0.010 | 1.93E-06 | -0.003 | 0.026 | 0.909 |
| rs1994140 | G | A | 0.604 | -0.046 | 0.009 | 9.99E-07 | -0.015 | 0.030 | 0.602 |
| rs208827 | G | C | 0.642 | 0.051 | 0.010 | 2.24E-07 | 0.053 | 0.026 | 0.042 |
| rs2206301 | C | T | 0.615 | -0.050 | 0.010 | 3.22E-07 | 0.055 | 0.026 | 0.035 |
| rs2673609 | G | C | 0.338 | 0.045 | 0.010 | 4.98E-06 | -0.008 | 0.026 | 0.759 |
| rs3923386 | T | C | 0.548 | -0.044 | 0.009 | 2.93E-06 | -0.051 | 0.025 | 0.039 |
| rs6790885 | A | G | 0.476 | -0.044 | 0.010 | 3.59E-06 | 0.011 | 0.025 | 0.649 |
| rs72802225 | G | A | 0.010 | 0.204 | 0.043 | 1.89E-06 | 0.198 | 0.123 | 0.107 |
| rs7332332 | G | A | 0.528 | -0.044 | 0.009 | 3.52E-06 | 0.019 | 0.025 | 0.438 |
| rs738475 | G | C | 0.272 | 0.051 | 0.011 | 2.54E-06 | 0.006 | 0.028 | 0.843 |
| rs79278977 | T | C | 0.007 | -0.194 | 0.042 | 3.53E-06 | 0.159 | 0.155 | 0.305 |
| rs865199 | G | A | 0.881 | 0.086 | 0.019 | 4.08E-06 | -0.052 | 0.038 | 0.176 |

Supplementary Table 34: Mendelian randomization analysis of insomnia on NIC.

| **SNP** | **A1** | **A2** | **EAF** | **insomnia** | | | **NIC** | | |
| --- | --- | --- | --- | --- | --- | --- | --- | --- | --- |
|  |  |  |  | **Beta** | **SE** | ***P-*val** | **Beta** | **SE** | ***P-*val** |
| rs112383419 | A | G | 0.038 | 0.094 | 0.020 | 2.40E-06 | -0.012 | 0.039 | 0.769 |
| rs113851554 | T | G | 0.071 | 0.178 | 0.020 | 2.14E-18 | 0.079 | 0.029 | 0.006 |
| rs114740697 | G | A | 0.014 | 0.143 | 0.031 | 4.58E-06 | 0.135 | 0.062 | 0.031 |
| rs115780514 | G | T | 0.035 | -0.166 | 0.034 | 1.17E-06 | -0.010 | 0.040 | 0.809 |
| rs116523352 | T | G | 0.001 | -0.174 | 0.036 | 1.76E-06 | 0.075 | 0.337 | 0.823 |
| rs140007258 | A | T | 0.012 | 0.177 | 0.038 | 2.85E-06 | -0.044 | 0.068 | 0.518 |
| rs145265872 | T | C | 0.044 | -0.107 | 0.023 | 3.02E-06 | -0.014 | 0.036 | 0.702 |
| rs147256205 | A | C | 0.020 | 0.188 | 0.041 | 4.63E-06 | 0.033 | 0.052 | 0.528 |
| rs149251474 | G | C | 0.017 | 0.158 | 0.034 | 2.77E-06 | 0.048 | 0.058 | 0.413 |
| rs1547668 | G | A | 0.858 | -0.054 | 0.012 | 3.61E-06 | -0.020 | 0.021 | 0.349 |
| rs1552291 | G | A | 0.651 | 0.047 | 0.010 | 1.93E-06 | 0.011 | 0.016 | 0.464 |
| rs1994140 | G | A | 0.605 | -0.046 | 0.009 | 9.99E-07 | -0.018 | 0.017 | 0.299 |
| rs208827 | G | C | 0.642 | 0.051 | 0.010 | 2.24E-07 | 0.013 | 0.015 | 0.408 |
| rs2206301 | C | T | 0.615 | -0.050 | 0.010 | 3.22E-07 | 0.012 | 0.015 | 0.433 |
| rs2673609 | G | C | 0.337 | 0.045 | 0.010 | 4.98E-06 | 0.012 | 0.016 | 0.433 |
| rs3923386 | T | C | 0.547 | -0.044 | 0.009 | 2.93E-06 | -0.023 | 0.015 | 0.120 |
| rs6790885 | A | G | 0.477 | -0.044 | 0.010 | 3.59E-06 | 0.026 | 0.015 | 0.083 |
| rs72802225 | G | A | 0.010 | 0.204 | 0.043 | 1.89E-06 | 0.106 | 0.074 | 0.154 |
| rs7332332 | G | A | 0.528 | -0.044 | 0.009 | 3.52E-06 | 0.005 | 0.015 | 0.717 |
| rs738475 | G | C | 0.273 | 0.051 | 0.011 | 2.54E-06 | 0.008 | 0.017 | 0.634 |
| rs79278977 | T | C | 0.007 | -0.194 | 0.042 | 3.53E-06 | 0.074 | 0.092 | 0.418 |
| rs865199 | G | A | 0.881 | 0.086 | 0.019 | 4.08E-06 | -0.011 | 0.023 | 0.641 |

Supplementary Table 35: Mendelian randomization analysis of insomnia on HF.

| **SNP** | **A1** | **A2** | **EAF** | **insomnia** | | | **HF** | | |
| --- | --- | --- | --- | --- | --- | --- | --- | --- | --- |
|  |  |  |  | **Beta** | **SE** | ***P-*val** | **Beta** | **SE** | ***P-*val** |
| rs112383419 | A | G | 0.061 | 0.094 | 0.020 | 2.40E-06 | -0.003 | 0.018 | 0.859 |
| rs113851554 | T | G | 0.056 | 0.178 | 0.020 | 2.14E-18 | 0.035 | 0.017 | 0.042 |
| rs114740697 | G | A | 0.026 | 0.143 | 0.031 | 4.58E-06 | -0.055 | 0.035 | 0.116 |
| rs115780514 | G | T | 0.019 | -0.166 | 0.034 | 1.17E-06 | -0.010 | 0.027 | 0.709 |
| rs116523352 | T | G | 0.019 | -0.174 | 0.036 | 1.76E-06 | 0.060 | 0.038 | 0.115 |
| rs117149835 | C | T | 0.013 | 0.198 | 0.043 | 3.49E-06 | -0.012 | 0.037 | 0.747 |
| rs140007258 | A | T | 0.017 | 0.177 | 0.038 | 2.85E-06 | 0.038 | 0.044 | 0.390 |
| rs145265872 | T | C | 0.046 | -0.107 | 0.023 | 3.02E-06 | -0.025 | 0.019 | 0.181 |
| rs147256205 | A | C | 0.014 | 0.188 | 0.041 | 4.63E-06 | 0.014 | 0.035 | 0.677 |
| rs149251474 | G | C | 0.022 | 0.158 | 0.034 | 2.77E-06 | -0.007 | 0.030 | 0.815 |
| rs1547668 | G | A | 0.796 | -0.054 | 0.012 | 3.61E-06 | -0.003 | 0.010 | 0.742 |
| rs1552291 | G | A | 0.629 | 0.047 | 0.010 | 1.93E-06 | -0.016 | 0.008 | 0.050 |
| rs208827 | G | C | 0.636 | 0.051 | 0.010 | 2.24E-07 | -0.006 | 0.008 | 0.495 |
| rs2206301 | C | T | 0.625 | -0.050 | 0.010 | 3.22E-07 | 0.001 | 0.008 | 0.923 |
| rs2673609 | G | C | 0.360 | 0.045 | 0.010 | 4.98E-06 | 0.011 | 0.008 | 0.188 |
| rs3923386 | T | C | 0.521 | -0.044 | 0.009 | 2.93E-06 | -0.018 | 0.008 | 0.025 |
| rs62515690 | A | G | 0.015 | 0.199 | 0.043 | 4.56E-06 | 0.026 | 0.035 | 0.454 |
| rs6790885 | A | G | 0.503 | -0.044 | 0.010 | 3.59E-06 | 0.002 | 0.008 | 0.785 |
| rs72802225 | G | A | 0.014 | 0.204 | 0.043 | 1.89E-06 | -0.011 | 0.036 | 0.755 |
| rs7332332 | G | A | 0.497 | -0.044 | 0.009 | 3.52E-06 | -0.014 | 0.008 | 0.076 |
| rs738475 | G | C | 0.259 | 0.051 | 0.011 | 2.54E-06 | -0.007 | 0.009 | 0.420 |
| rs865199 | G | A | 0.932 | 0.086 | 0.019 | 4.08E-06 | -0.008 | 0.015 | 0.597 |

Supplementary Table 36: Mendelian randomization analysis of insomnia on ISla.

| **SNP** | **A1** | **A2** | **EAF** | **insomnia** | | | **ISla** | | |
| --- | --- | --- | --- | --- | --- | --- | --- | --- | --- |
|  |  |  |  | **Beta** | **SE** | ***P-*val** | **Beta** | **SE** | ***P-*val** |
| rs112383419 | A | G | 0.056 | 0.094 | 0.020 | 2.40E-06 | 0.077 | 0.060 | 0.197 |
| rs113851554 | T | G | 0.063 | 0.178 | 0.020 | 2.14E-18 | 0.028 | 0.056 | 0.612 |
| rs114740697 | G | A | 0.026 | 0.143 | 0.031 | 4.58E-06 | 0.166 | 0.106 | 0.116 |
| rs115780514 | G | T | 0.020 | -0.166 | 0.034 | 1.17E-06 | 0.162 | 0.098 | 0.100 |
| rs116523352 | T | G | 0.020 | -0.174 | 0.036 | 1.76E-06 | 0.086 | 0.131 | 0.515 |
| rs117149835 | C | T | 0.012 | 0.198 | 0.043 | 3.49E-06 | 0.127 | 0.152 | 0.403 |
| rs140007258 | A | T | 0.017 | 0.177 | 0.038 | 2.85E-06 | -0.210 | 0.137 | 0.125 |
| rs145265872 | T | C | 0.050 | -0.107 | 0.023 | 3.02E-06 | 0.011 | 0.066 | 0.863 |
| rs147256205 | A | C | 0.015 | 0.188 | 0.041 | 4.63E-06 | -0.204 | 0.141 | 0.148 |
| rs149251474 | G | C | 0.021 | 0.158 | 0.034 | 2.77E-06 | 0.001 | 0.111 | 0.991 |
| rs1547668 | G | A | 0.800 | -0.054 | 0.012 | 3.61E-06 | -0.054 | 0.031 | 0.083 |
| rs1552291 | G | A | 0.649 | 0.047 | 0.010 | 1.93E-06 | 0.035 | 0.027 | 0.188 |
| rs208827 | G | C | 0.650 | 0.051 | 0.010 | 2.24E-07 | 0.004 | 0.027 | 0.892 |
| rs2206301 | C | T | 0.629 | -0.050 | 0.010 | 3.22E-07 | 0.015 | 0.026 | 0.575 |
| rs2673609 | G | C | 0.358 | 0.045 | 0.010 | 4.98E-06 | 0.057 | 0.026 | 0.030 |
| rs3923386 | T | C | 0.533 | -0.044 | 0.009 | 2.93E-06 | -0.068 | 0.025 | 0.006 |
| rs62515690 | A | G | 0.018 | 0.199 | 0.043 | 4.56E-06 | 0.292 | 0.132 | 0.027 |
| rs6790885 | A | G | 0.477 | -0.044 | 0.010 | 3.59E-06 | 0.049 | 0.026 | 0.061 |
| rs72802225 | G | A | 0.017 | 0.204 | 0.043 | 1.89E-06 | 0.190 | 0.135 | 0.160 |
| rs7332332 | G | A | 0.499 | -0.044 | 0.009 | 3.52E-06 | 0.022 | 0.025 | 0.374 |
| rs738475 | G | C | 0.279 | 0.051 | 0.011 | 2.54E-06 | -0.041 | 0.028 | 0.145 |
| rs79278977 | T | C | 0.016 | -0.194 | 0.042 | 3.53E-06 | 0.157 | 0.165 | 0.341 |
| rs865199 | G | A | 0.929 | 0.086 | 0.019 | 4.08E-06 | -0.042 | 0.051 | 0.412 |

Supplementary Table 37: Mendelian randomization analysis of depressed affect on CAD.

| **SNP** | **A1** | **A2** | **EAF** | **depressed affect** | | | **CAD** | | |
| --- | --- | --- | --- | --- | --- | --- | --- | --- | --- |
|  |  |  |  | **Beta** | **SE** | ***P-*val** | **Beta** | **SE** | ***P-*val** |
| rs10020288 | A | G | 0.360 | -0.013 | 0.002 | 2.56E-08 | -0.010 | 0.007 | 0.170 |
| rs10144845 | T | C | 0.683 | 0.016 | 0.002 | 1.05E-10 | 0.016 | 0.007 | 0.037 |
| rs10156548 | C | G | 0.643 | -0.018 | 0.002 | 2.55E-13 | 0.018 | 0.007 | 0.014 |
| rs11209175 | T | C | 0.635 | -0.015 | 0.002 | 1.65E-09 | -0.004 | 0.007 | 0.570 |
| rs11605020 | A | G | 0.514 | 0.014 | 0.002 | 3.59E-09 | 0.006 | 0.007 | 0.380 |
| rs11693031 | G | A | 0.321 | -0.016 | 0.003 | 4.59E-10 | -0.011 | 0.008 | 0.160 |
| rs12030991 | C | G | 0.762 | 0.015 | 0.003 | 3.05E-08 | 0.014 | 0.008 | 0.075 |
| rs12137936 | G | C | 0.314 | 0.014 | 0.002 | 2.94E-08 | 0.007 | 0.008 | 0.390 |
| rs12938775 | A | G | 0.503 | -0.013 | 0.002 | 2.49E-08 | -0.005 | 0.007 | 0.490 |
| rs13122395 | A | G | 0.326 | 0.014 | 0.002 | 3.63E-08 | -0.007 | 0.007 | 0.320 |
| rs1782179 | C | T | 0.727 | 0.015 | 0.003 | 1.50E-08 | -0.001 | 0.008 | 0.890 |
| rs2042555 | A | G | 0.415 | 0.014 | 0.002 | 9.45E-10 | -0.003 | 0.007 | 0.630 |
| rs2149351 | G | T | 0.758 | -0.016 | 0.003 | 2.27E-09 | -0.011 | 0.008 | 0.170 |
| rs28893517 | G | A | 0.068 | 0.028 | 0.005 | 5.16E-10 | -0.004 | 0.014 | 0.770 |
| rs297346 | G | A | 0.638 | -0.016 | 0.002 | 1.81E-11 | -0.009 | 0.007 | 0.230 |
| rs35738585 | G | T | 0.434 | -0.017 | 0.002 | 1.73E-13 | -0.014 | 0.007 | 0.043 |
| rs35755513 | T | C | 0.074 | 0.024 | 0.004 | 4.12E-08 | 0.011 | 0.014 | 0.420 |
| rs3843954 | C | G | 0.292 | 0.014 | 0.003 | 3.82E-08 | 0.014 | 0.008 | 0.080 |
| rs4578918 | C | T | 0.740 | -0.018 | 0.003 | 1.03E-11 | -0.003 | 0.008 | 0.730 |
| rs4632195 | T | C | 0.513 | 0.016 | 0.002 | 6.63E-12 | 0.013 | 0.007 | 0.061 |
| rs55965054 | T | C | 0.559 | -0.014 | 0.002 | 2.67E-09 | -0.002 | 0.007 | 0.810 |
| rs59382200 | G | A | 0.401 | -0.016 | 0.002 | 2.44E-11 | -0.007 | 0.007 | 0.330 |
| rs599550 | A | G | 0.850 | 0.027 | 0.003 | 3.69E-17 | 0.008 | 0.010 | 0.440 |
| rs60393230 | G | A | 0.596 | 0.014 | 0.002 | 3.57E-09 | 0.026 | 0.007 | 0.000 |
| rs62172117 | A | G | 0.357 | -0.014 | 0.002 | 6.19E-09 | 0.004 | 0.007 | 0.630 |
| rs6818081 | T | C | 0.148 | 0.018 | 0.003 | 2.83E-08 | 0.009 | 0.010 | 0.380 |
| rs6900114 | A | G | 0.265 | -0.015 | 0.003 | 2.30E-08 | 0.004 | 0.008 | 0.610 |
| rs7175083 | C | T | 0.522 | -0.015 | 0.002 | 2.21E-10 | 0.005 | 0.007 | 0.460 |
| rs75650221 | T | C | 0.038 | -0.036 | 0.006 | 3.08E-09 | 0.011 | 0.018 | 0.540 |
| rs76923064 | C | T | 0.013 | 0.056 | 0.010 | 3.24E-08 | 0.000 | 0.031 | 1.000 |
| rs77087420 | G | A | 0.055 | -0.033 | 0.005 | 1.06E-10 | -0.005 | 0.016 | 0.770 |
| rs7714426 | G | C | 0.849 | -0.018 | 0.003 | 1.41E-08 | -0.004 | 0.010 | 0.700 |
| rs77607745 | C | A | 0.190 | 0.016 | 0.003 | 4.95E-08 | 0.009 | 0.009 | 0.300 |
| rs7912226 | A | T | 0.397 | -0.015 | 0.002 | 8.12E-10 | -0.002 | 0.007 | 0.810 |
| rs836927 | A | C | 0.427 | 0.015 | 0.002 | 4.11E-10 | -0.006 | 0.007 | 0.400 |
| rs9852417 | A | C | 0.590 | -0.013 | 0.002 | 2.84E-08 | -0.015 | 0.007 | 0.042 |
| rs9858071 | C | T | 0.288 | 0.016 | 0.003 | 3.25E-10 | -0.013 | 0.008 | 0.081 |
| rs9930139 | C | A | 0.588 | 0.014 | 0.002 | 1.19E-08 | -0.006 | 0.007 | 0.420 |

Supplementary Table 38: Mendelian randomization analysis of depressed affect on CA.

| **SNP** | **A1** | **A2** | **EAF** | **depressed affect** | | | **CA** | | |
| --- | --- | --- | --- | --- | --- | --- | --- | --- | --- |
|  |  |  |  | **Beta** | **SE** | ***P-*val** | **Beta** | **SE** | ***P-*val** |
| rs10020288 | A | G | 0.354 | -0.013 | 0.002 | 2.56E-08 | 0.002 | 0.020 | 0.917 |
| rs10144845 | T | C | 0.611 | 0.016 | 0.002 | 1.05E-10 | -0.026 | 0.019 | 0.185 |
| rs10156548 | C | G | 0.656 | -0.018 | 0.002 | 2.55E-13 | 0.007 | 0.020 | 0.736 |
| rs11209175 | T | C | 0.486 | -0.015 | 0.002 | 1.65E-09 | -0.037 | 0.019 | 0.051 |
| rs11605020 | A | G | 0.399 | 0.014 | 0.002 | 3.59E-09 | 0.009 | 0.019 | 0.651 |
| rs11693031 | G | A | 0.326 | -0.016 | 0.003 | 4.59E-10 | -0.003 | 0.020 | 0.889 |
| rs12137936 | G | C | 0.292 | 0.014 | 0.002 | 2.94E-08 | 0.016 | 0.021 | 0.436 |
| rs13122395 | A | G | 0.240 | 0.014 | 0.002 | 3.63E-08 | -0.032 | 0.022 | 0.151 |
| rs2042555 | A | G | 0.495 | 0.014 | 0.002 | 9.45E-10 | -0.005 | 0.019 | 0.781 |
| rs2149351 | G | T | 0.818 | -0.016 | 0.003 | 2.27E-09 | 0.008 | 0.025 | 0.746 |
| rs28893517 | G | A | 0.026 | 0.028 | 0.005 | 5.16E-10 | -0.024 | 0.060 | 0.691 |
| rs297346 | G | A | 0.753 | -0.016 | 0.002 | 1.81E-11 | -0.020 | 0.022 | 0.360 |
| rs35738585 | G | T | 0.261 | -0.017 | 0.002 | 1.73E-13 | -0.040 | 0.022 | 0.067 |
| rs35755513 | T | C | 0.062 | 0.024 | 0.004 | 4.12E-08 | 0.030 | 0.040 | 0.443 |
| rs3843954 | C | G | 0.263 | 0.014 | 0.003 | 3.82E-08 | 0.031 | 0.022 | 0.150 |
| rs4578918 | C | T | 0.730 | -0.018 | 0.003 | 1.03E-11 | 0.007 | 0.021 | 0.753 |
| rs4632195 | T | C | 0.468 | 0.016 | 0.002 | 6.63E-12 | 0.023 | 0.019 | 0.236 |
| rs55965054 | T | C | 0.534 | -0.014 | 0.002 | 2.67E-09 | -0.026 | 0.019 | 0.175 |
| rs59382200 | G | A | 0.453 | -0.016 | 0.002 | 2.44E-11 | -0.021 | 0.019 | 0.281 |
| rs599550 | A | G | 0.870 | 0.027 | 0.003 | 3.69E-17 | 0.016 | 0.028 | 0.586 |
| rs60393230 | G | A | 0.627 | 0.014 | 0.002 | 3.57E-09 | 0.007 | 0.020 | 0.719 |
| rs62172117 | A | G | 0.327 | -0.014 | 0.002 | 6.19E-09 | -0.004 | 0.020 | 0.833 |
| rs6818081 | T | C | 0.153 | 0.018 | 0.003 | 2.83E-08 | 0.013 | 0.026 | 0.611 |
| rs6900114 | A | G | 0.266 | -0.015 | 0.003 | 2.30E-08 | 0.000 | 0.022 | 0.998 |
| rs7175083 | C | T | 0.484 | -0.015 | 0.002 | 2.21E-10 | -0.016 | 0.019 | 0.396 |
| rs75650221 | T | C | 0.032 | -0.036 | 0.006 | 3.08E-09 | 0.007 | 0.054 | 0.898 |
| rs76923064 | C | T | 0.023 | 0.056 | 0.010 | 3.24E-08 | 0.039 | 0.064 | 0.542 |
| rs77087420 | G | A | 0.048 | -0.033 | 0.005 | 1.06E-10 | 0.010 | 0.044 | 0.824 |
| rs7714426 | G | C | 0.793 | -0.018 | 0.003 | 1.41E-08 | 0.021 | 0.023 | 0.377 |
| rs77607745 | C | A | 0.172 | 0.016 | 0.003 | 4.95E-08 | -0.015 | 0.025 | 0.561 |
| rs836927 | A | C | 0.473 | 0.015 | 0.002 | 4.11E-10 | -0.001 | 0.019 | 0.952 |
| rs9852417 | A | C | 0.594 | -0.013 | 0.002 | 2.84E-08 | -0.057 | 0.019 | 0.004 |
| rs9858071 | C | T | 0.291 | 0.016 | 0.003 | 3.25E-10 | -0.033 | 0.021 | 0.110 |
| rs9930139 | C | A | 0.594 | 0.014 | 0.002 | 1.19E-08 | -0.004 | 0.019 | 0.832 |

Supplementary Table 39: Mendelian randomization analysis of depressed affect on AF.

| **SNP** | **A1** | **A2** | **EAF** | **depressed affect** | | | **AF** | | |
| --- | --- | --- | --- | --- | --- | --- | --- | --- | --- |
|  |  |  |  | **Beta** | **SE** | ***P-*val** | **Beta** | **SE** | ***P-*val** |
| rs10020288 | A | G | 0.353 | -0.013 | 0.002 | 2.56E-08 | -0.006 | 0.016 | 0.711 |
| rs10144845 | T | C | 0.611 | 0.016 | 0.002 | 1.05E-10 | -0.003 | 0.015 | 0.836 |
| rs10156548 | C | G | 0.655 | -0.018 | 0.002 | 2.55E-13 | 0.013 | 0.016 | 0.395 |
| rs11209175 | T | C | 0.487 | -0.015 | 0.002 | 1.65E-09 | -0.029 | 0.015 | 0.052 |
| rs11605020 | A | G | 0.399 | 0.014 | 0.002 | 3.59E-09 | -0.001 | 0.015 | 0.931 |
| rs11693031 | G | A | 0.327 | -0.016 | 0.003 | 4.59E-10 | 0.011 | 0.016 | 0.504 |
| rs12137936 | G | C | 0.290 | 0.014 | 0.002 | 2.94E-08 | -0.014 | 0.016 | 0.387 |
| rs13122395 | A | G | 0.240 | 0.014 | 0.002 | 3.63E-08 | 0.010 | 0.017 | 0.573 |
| rs2042555 | A | G | 0.493 | 0.014 | 0.002 | 9.45E-10 | -0.009 | 0.015 | 0.565 |
| rs2149351 | G | T | 0.818 | -0.016 | 0.003 | 2.27E-09 | 0.002 | 0.019 | 0.934 |
| rs28893517 | G | A | 0.027 | 0.028 | 0.005 | 5.16E-10 | -0.050 | 0.046 | 0.278 |
| rs297346 | G | A | 0.752 | -0.016 | 0.002 | 1.81E-11 | 0.014 | 0.017 | 0.408 |
| rs35738585 | G | T | 0.261 | -0.017 | 0.002 | 1.73E-13 | -0.034 | 0.017 | 0.047 |
| rs35755513 | T | C | 0.062 | 0.024 | 0.004 | 4.12E-08 | 0.030 | 0.031 | 0.328 |
| rs3843954 | C | G | 0.263 | 0.014 | 0.003 | 3.82E-08 | 0.030 | 0.017 | 0.073 |
| rs4578918 | C | T | 0.730 | -0.018 | 0.003 | 1.03E-11 | -0.011 | 0.017 | 0.501 |
| rs4632195 | T | C | 0.467 | 0.016 | 0.002 | 6.63E-12 | 0.025 | 0.015 | 0.088 |
| rs55965054 | T | C | 0.535 | -0.014 | 0.002 | 2.67E-09 | -0.026 | 0.015 | 0.086 |
| rs59382200 | G | A | 0.452 | -0.016 | 0.002 | 2.44E-11 | -0.010 | 0.015 | 0.507 |
| rs599550 | A | G | 0.870 | 0.027 | 0.003 | 3.69E-17 | -0.058 | 0.022 | 0.009 |
| rs60393230 | G | A | 0.626 | 0.014 | 0.002 | 3.57E-09 | 0.020 | 0.015 | 0.194 |
| rs62172117 | A | G | 0.327 | -0.014 | 0.002 | 6.19E-09 | -0.033 | 0.016 | 0.037 |
| rs6818081 | T | C | 0.153 | 0.018 | 0.003 | 2.83E-08 | -0.003 | 0.021 | 0.902 |
| rs6900114 | A | G | 0.265 | -0.015 | 0.003 | 2.30E-08 | -0.035 | 0.017 | 0.037 |
| rs7175083 | C | T | 0.483 | -0.015 | 0.002 | 2.21E-10 | 0.009 | 0.015 | 0.542 |
| rs75650221 | T | C | 0.033 | -0.036 | 0.006 | 3.08E-09 | -0.005 | 0.042 | 0.909 |
| rs76923064 | C | T | 0.023 | 0.056 | 0.010 | 3.24E-08 | 0.076 | 0.050 | 0.130 |
| rs77087420 | G | A | 0.048 | -0.033 | 0.005 | 1.06E-10 | -0.002 | 0.034 | 0.952 |
| rs7714426 | G | C | 0.793 | -0.018 | 0.003 | 1.41E-08 | 0.044 | 0.018 | 0.016 |
| rs77607745 | C | A | 0.172 | 0.016 | 0.003 | 4.95E-08 | -0.006 | 0.020 | 0.755 |
| rs836927 | A | C | 0.473 | 0.015 | 0.002 | 4.11E-10 | 0.001 | 0.015 | 0.929 |
| rs9852417 | A | C | 0.594 | -0.013 | 0.002 | 2.84E-08 | -0.024 | 0.015 | 0.116 |
| rs9858071 | C | T | 0.291 | 0.016 | 0.003 | 3.25E-10 | 0.002 | 0.016 | 0.891 |
| rs9930139 | C | A | 0.594 | 0.014 | 0.002 | 1.19E-08 | 0.009 | 0.015 | 0.567 |

Supplementary Table 40: Mendelian randomization analysis of depressed affect on HHD.

| **SNP** | **A1** | **A2** | **EAF** | **depressed affect** | | | **HHD** | | |
| --- | --- | --- | --- | --- | --- | --- | --- | --- | --- |
|  |  |  |  | **Beta** | **SE** | ***P-*val** | **Beta** | **SE** | ***P-*val** |
| rs10020288 | A | G | 0.353 | -0.013 | 0.002 | 2.56E-08 | -0.023 | 0.026 | 0.381 |
| rs10144845 | T | C | 0.611 | 0.016 | 0.002 | 1.05E-10 | -0.009 | 0.026 | 0.739 |
| rs10156548 | C | G | 0.655 | -0.018 | 0.002 | 2.55E-13 | -0.023 | 0.026 | 0.381 |
| rs11209175 | T | C | 0.487 | -0.015 | 0.002 | 1.65E-09 | -0.043 | 0.025 | 0.088 |
| rs11605020 | A | G | 0.399 | 0.014 | 0.002 | 3.59E-09 | -0.034 | 0.026 | 0.188 |
| rs11693031 | G | A | 0.327 | -0.016 | 0.003 | 4.59E-10 | 0.038 | 0.027 | 0.156 |
| rs12137936 | G | C | 0.291 | 0.014 | 0.002 | 2.94E-08 | 0.018 | 0.028 | 0.511 |
| rs13122395 | A | G | 0.240 | 0.014 | 0.002 | 3.63E-08 | 0.036 | 0.029 | 0.216 |
| rs2042555 | A | G | 0.495 | 0.014 | 0.002 | 9.45E-10 | 0.002 | 0.025 | 0.948 |
| rs2149351 | G | T | 0.819 | -0.016 | 0.003 | 2.27E-09 | -0.006 | 0.033 | 0.853 |
| rs28893517 | G | A | 0.026 | 0.028 | 0.005 | 5.16E-10 | -0.068 | 0.078 | 0.386 |
| rs297346 | G | A | 0.753 | -0.016 | 0.002 | 1.81E-11 | -0.028 | 0.029 | 0.343 |
| rs35738585 | G | T | 0.261 | -0.017 | 0.002 | 1.73E-13 | -0.071 | 0.029 | 0.013 |
| rs35755513 | T | C | 0.062 | 0.024 | 0.004 | 4.12E-08 | -0.043 | 0.051 | 0.404 |
| rs3843954 | C | G | 0.263 | 0.014 | 0.003 | 3.82E-08 | 0.070 | 0.029 | 0.014 |
| rs4578918 | C | T | 0.731 | -0.018 | 0.003 | 1.03E-11 | -0.020 | 0.028 | 0.466 |
| rs4632195 | T | C | 0.469 | 0.016 | 0.002 | 6.63E-12 | -0.007 | 0.025 | 0.792 |
| rs55965054 | T | C | 0.535 | -0.014 | 0.002 | 2.67E-09 | 0.023 | 0.025 | 0.364 |
| rs59382200 | G | A | 0.452 | -0.016 | 0.002 | 2.44E-11 | 0.010 | 0.025 | 0.705 |
| rs599550 | A | G | 0.870 | 0.027 | 0.003 | 3.69E-17 | -0.005 | 0.037 | 0.893 |
| rs60393230 | G | A | 0.626 | 0.014 | 0.002 | 3.57E-09 | 0.001 | 0.026 | 0.958 |
| rs62172117 | A | G | 0.327 | -0.014 | 0.002 | 6.19E-09 | -0.043 | 0.027 | 0.109 |
| rs6818081 | T | C | 0.153 | 0.018 | 0.003 | 2.83E-08 | 0.029 | 0.035 | 0.404 |
| rs6900114 | A | G | 0.266 | -0.015 | 0.003 | 2.30E-08 | -0.014 | 0.028 | 0.613 |
| rs7175083 | C | T | 0.483 | -0.015 | 0.002 | 2.21E-10 | 0.046 | 0.025 | 0.063 |
| rs75650221 | T | C | 0.032 | -0.036 | 0.006 | 3.08E-09 | -0.003 | 0.071 | 0.964 |
| rs76923064 | C | T | 0.023 | 0.056 | 0.010 | 3.24E-08 | 0.052 | 0.083 | 0.532 |
| rs77087420 | G | A | 0.049 | -0.033 | 0.005 | 1.06E-10 | 0.006 | 0.058 | 0.916 |
| rs7714426 | G | C | 0.793 | -0.018 | 0.003 | 1.41E-08 | -0.038 | 0.031 | 0.220 |
| rs77607745 | C | A | 0.172 | 0.016 | 0.003 | 4.95E-08 | 0.025 | 0.033 | 0.454 |
| rs836927 | A | C | 0.473 | 0.015 | 0.002 | 4.11E-10 | 0.004 | 0.025 | 0.873 |
| rs9852417 | A | C | 0.594 | -0.013 | 0.002 | 2.84E-08 | -0.044 | 0.025 | 0.084 |
| rs9858071 | C | T | 0.291 | 0.016 | 0.003 | 3.25E-10 | 0.051 | 0.028 | 0.065 |
| rs9930139 | C | A | 0.594 | 0.014 | 0.002 | 1.19E-08 | -0.004 | 0.025 | 0.871 |

Supplementary Table 41: Mendelian randomization analysis of depressed affect on NIC.

| **SNP** | **A1** | **A2** | **EAF** | **depressed affect** | | | **NIC** | | |
| --- | --- | --- | --- | --- | --- | --- | --- | --- | --- |
|  |  |  |  | **Beta** | **SE** | ***P-*val** | **Beta** | **SE** | ***P-*val** |
| rs10020288 | A | G | 0.354 | -0.013 | 0.002 | 2.56E-08 | -0.016 | 0.015 | 0.296 |
| rs10144845 | T | C | 0.611 | 0.016 | 0.002 | 1.05E-10 | -0.019 | 0.015 | 0.199 |
| rs10156548 | C | G | 0.656 | -0.018 | 0.002 | 2.55E-13 | 0.014 | 0.015 | 0.371 |
| rs11209175 | T | C | 0.487 | -0.015 | 0.002 | 1.65E-09 | -0.022 | 0.015 | 0.132 |
| rs11605020 | A | G | 0.399 | 0.014 | 0.002 | 3.59E-09 | 0.005 | 0.015 | 0.752 |
| rs11693031 | G | A | 0.326 | -0.016 | 0.003 | 4.59E-10 | 0.006 | 0.016 | 0.720 |
| rs12137936 | G | C | 0.291 | 0.014 | 0.002 | 2.94E-08 | -0.017 | 0.016 | 0.304 |
| rs13122395 | A | G | 0.240 | 0.014 | 0.002 | 3.63E-08 | -0.002 | 0.017 | 0.927 |
| rs2042555 | A | G | 0.495 | 0.014 | 0.002 | 9.45E-10 | -0.026 | 0.015 | 0.079 |
| rs2149351 | G | T | 0.818 | -0.016 | 0.003 | 2.27E-09 | 0.010 | 0.019 | 0.599 |
| rs28893517 | G | A | 0.026 | 0.028 | 0.005 | 5.16E-10 | -0.040 | 0.046 | 0.385 |
| rs297346 | G | A | 0.753 | -0.016 | 0.002 | 1.81E-11 | 0.017 | 0.017 | 0.330 |
| rs35738585 | G | T | 0.262 | -0.017 | 0.002 | 1.73E-13 | -0.026 | 0.017 | 0.121 |
| rs35755513 | T | C | 0.062 | 0.024 | 0.004 | 4.12E-08 | -0.005 | 0.031 | 0.871 |
| rs3843954 | C | G | 0.263 | 0.014 | 0.003 | 3.82E-08 | 0.003 | 0.017 | 0.876 |
| rs4578918 | C | T | 0.730 | -0.018 | 0.003 | 1.03E-11 | -0.001 | 0.017 | 0.944 |
| rs4632195 | T | C | 0.468 | 0.016 | 0.002 | 6.63E-12 | 0.024 | 0.015 | 0.108 |
| rs55965054 | T | C | 0.535 | -0.014 | 0.002 | 2.67E-09 | 0.011 | 0.015 | 0.473 |
| rs59382200 | G | A | 0.453 | -0.016 | 0.002 | 2.44E-11 | -0.025 | 0.015 | 0.090 |
| rs599550 | A | G | 0.870 | 0.027 | 0.003 | 3.69E-17 | -0.065 | 0.022 | 0.003 |
| rs60393230 | G | A | 0.627 | 0.014 | 0.002 | 3.57E-09 | 0.046 | 0.015 | 0.002 |
| rs62172117 | A | G | 0.327 | -0.014 | 0.002 | 6.19E-09 | -0.028 | 0.016 | 0.072 |
| rs6818081 | T | C | 0.153 | 0.018 | 0.003 | 2.83E-08 | -0.023 | 0.020 | 0.269 |
| rs6900114 | A | G | 0.266 | -0.015 | 0.003 | 2.30E-08 | -0.020 | 0.017 | 0.233 |
| rs7175083 | C | T | 0.484 | -0.015 | 0.002 | 2.21E-10 | 0.018 | 0.015 | 0.216 |
| rs75650221 | T | C | 0.033 | -0.036 | 0.006 | 3.08E-09 | -0.037 | 0.042 | 0.374 |
| rs76923064 | C | T | 0.023 | 0.056 | 0.010 | 3.24E-08 | 0.017 | 0.050 | 0.735 |
| rs77087420 | G | A | 0.048 | -0.033 | 0.005 | 1.06E-10 | 0.019 | 0.034 | 0.579 |
| rs7714426 | G | C | 0.793 | -0.018 | 0.003 | 1.41E-08 | -0.017 | 0.018 | 0.344 |
| rs77607745 | C | A | 0.172 | 0.016 | 0.003 | 4.95E-08 | 0.000 | 0.020 | 0.991 |
| rs836927 | A | C | 0.473 | 0.015 | 0.002 | 4.11E-10 | 0.043 | 0.015 | 0.004 |
| rs9852417 | A | C | 0.594 | -0.013 | 0.002 | 2.84E-08 | -0.031 | 0.015 | 0.039 |
| rs9858071 | C | T | 0.291 | 0.016 | 0.003 | 3.25E-10 | 0.026 | 0.016 | 0.109 |
| rs9930139 | C | A | 0.594 | 0.014 | 0.002 | 1.19E-08 | 0.009 | 0.015 | 0.568 |

Supplementary Table 42: Mendelian randomization analysis of depressed affect on HF.

| **SNP** | **A1** | **A2** | **EAF** | **depressed affect** | | | **HF** | | |
| --- | --- | --- | --- | --- | --- | --- | --- | --- | --- |
|  |  |  |  | **Beta** | **SE** | ***P-*val** | **Beta** | **SE** | ***P-*val** |
| rs10020288 | A | G | 0.360 | -0.013 | 0.002 | 2.56E-08 | 0.001 | 0.008 | 0.903 |
| rs10144845 | T | C | 0.683 | 0.016 | 0.002 | 1.05E-10 | -0.010 | 0.008 | 0.216 |
| rs10156548 | C | G | 0.643 | -0.018 | 0.002 | 2.55E-13 | 0.019 | 0.008 | 0.018 |
| rs11209175 | T | C | 0.635 | -0.015 | 0.002 | 1.65E-09 | -0.007 | 0.008 | 0.365 |
| rs11605020 | A | G | 0.514 | 0.014 | 0.002 | 3.59E-09 | 0.006 | 0.008 | 0.474 |
| rs11693031 | G | A | 0.321 | -0.016 | 0.003 | 4.59E-10 | -0.012 | 0.009 | 0.150 |
| rs12030991 | C | G | 0.762 | 0.015 | 0.003 | 3.05E-08 | 0.008 | 0.009 | 0.395 |
| rs12137936 | G | C | 0.314 | 0.014 | 0.002 | 2.94E-08 | 0.013 | 0.008 | 0.126 |
| rs12938775 | A | G | 0.503 | -0.013 | 0.002 | 2.49E-08 | -0.002 | 0.008 | 0.798 |
| rs13122395 | A | G | 0.326 | 0.014 | 0.002 | 3.63E-08 | -0.010 | 0.008 | 0.237 |
| rs1782179 | C | T | 0.727 | 0.015 | 0.003 | 1.50E-08 | -0.001 | 0.010 | 0.917 |
| rs2042555 | A | G | 0.415 | 0.014 | 0.002 | 9.45E-10 | -0.010 | 0.008 | 0.211 |
| rs2149351 | G | T | 0.758 | -0.016 | 0.003 | 2.27E-09 | -0.007 | 0.009 | 0.443 |
| rs28893517 | G | A | 0.068 | 0.028 | 0.005 | 5.16E-10 | 0.023 | 0.017 | 0.178 |
| rs297346 | G | A | 0.638 | -0.016 | 0.002 | 1.81E-11 | -0.010 | 0.008 | 0.208 |
| rs35738585 | G | T | 0.434 | -0.017 | 0.002 | 1.73E-13 | -0.007 | 0.008 | 0.373 |
| rs35755513 | T | C | 0.074 | 0.024 | 0.004 | 4.12E-08 | 0.002 | 0.017 | 0.887 |
| rs3843954 | C | G | 0.292 | 0.014 | 0.003 | 3.82E-08 | 0.013 | 0.009 | 0.143 |
| rs4578918 | C | T | 0.740 | -0.018 | 0.003 | 1.03E-11 | -0.017 | 0.009 | 0.054 |
| rs4632195 | T | C | 0.513 | 0.016 | 0.002 | 6.63E-12 | 0.002 | 0.008 | 0.843 |
| rs55965054 | T | C | 0.559 | -0.014 | 0.002 | 2.67E-09 | -0.001 | 0.008 | 0.943 |
| rs59382200 | G | A | 0.401 | -0.016 | 0.002 | 2.44E-11 | 0.001 | 0.008 | 0.931 |
| rs599550 | A | G | 0.850 | 0.027 | 0.003 | 3.69E-17 | -0.004 | 0.011 | 0.717 |
| rs60393230 | G | A | 0.596 | 0.014 | 0.002 | 3.57E-09 | -0.004 | 0.008 | 0.626 |
| rs62172117 | A | G | 0.357 | -0.014 | 0.002 | 6.19E-09 | -0.001 | 0.008 | 0.945 |
| rs6818081 | T | C | 0.148 | 0.018 | 0.003 | 2.83E-08 | 0.010 | 0.011 | 0.351 |
| rs6900114 | A | G | 0.265 | -0.015 | 0.003 | 2.30E-08 | -0.014 | 0.009 | 0.119 |
| rs7175083 | C | T | 0.522 | -0.015 | 0.002 | 2.21E-10 | 0.015 | 0.008 | 0.062 |
| rs75650221 | T | C | 0.038 | -0.036 | 0.006 | 3.08E-09 | -0.016 | 0.021 | 0.455 |
| rs76923064 | C | T | 0.013 | 0.056 | 0.010 | 3.24E-08 | -0.004 | 0.032 | 0.896 |
| rs77087420 | G | A | 0.055 | -0.033 | 0.005 | 1.06E-10 | -0.034 | 0.018 | 0.064 |
| rs7714426 | G | C | 0.849 | -0.018 | 0.003 | 1.41E-08 | -0.014 | 0.011 | 0.203 |
| rs77607745 | C | A | 0.190 | 0.016 | 0.003 | 4.95E-08 | 0.001 | 0.010 | 0.937 |
| rs7912226 | A | T | 0.397 | -0.015 | 0.002 | 8.12E-10 | -0.012 | 0.010 | 0.203 |
| rs836927 | A | C | 0.427 | 0.015 | 0.002 | 4.11E-10 | 0.002 | 0.008 | 0.785 |
| rs9852417 | A | C | 0.590 | -0.013 | 0.002 | 2.84E-08 | -0.017 | 0.008 | 0.031 |
| rs9858071 | C | T | 0.288 | 0.016 | 0.003 | 3.25E-10 | 0.025 | 0.009 | 0.004 |
| rs9930139 | C | A | 0.588 | 0.014 | 0.002 | 1.19E-08 | -0.009 | 0.008 | 0.260 |

Supplementary Table 43: Mendelian randomization analysis of depressed affect on ISla.

| **SNP** | **A1** | **A2** | **EAF** | **depressed affect** | | | **ISla** | | |
| --- | --- | --- | --- | --- | --- | --- | --- | --- | --- |
|  |  |  |  | **Beta** | **SE** | ***P-*val** | **Beta** | **SE** | ***P-*val** |
| rs10020288 | A | G | 0.353 | -0.013 | 0.002 | 2.56E-08 | 0.082 | 0.026 | 0.002 |
| rs10144845 | T | C | 0.683 | 0.016 | 0.002 | 1.05E-10 | 0.008 | 0.026 | 0.755 |
| rs10156548 | C | G | 0.643 | -0.018 | 0.002 | 2.55E-13 | -0.020 | 0.026 | 0.434 |
| rs11209175 | T | C | 0.630 | -0.015 | 0.002 | 1.65E-09 | -0.013 | 0.026 | 0.614 |
| rs11605020 | A | G | 0.497 | 0.014 | 0.002 | 3.59E-09 | -0.052 | 0.025 | 0.039 |
| rs11693031 | G | A | 0.334 | -0.016 | 0.003 | 4.59E-10 | -0.003 | 0.029 | 0.926 |
| rs12030991 | C | G | 0.751 | 0.015 | 0.003 | 3.05E-08 | 0.017 | 0.029 | 0.551 |
| rs12137936 | G | C | 0.314 | 0.014 | 0.002 | 2.94E-08 | 0.037 | 0.026 | 0.159 |
| rs12938775 | A | G | 0.504 | -0.013 | 0.002 | 2.49E-08 | -0.012 | 0.026 | 0.651 |
| rs13122395 | A | G | 0.317 | 0.014 | 0.002 | 3.63E-08 | 0.006 | 0.027 | 0.827 |
| rs1782179 | C | T | 0.738 | 0.015 | 0.003 | 1.50E-08 | 0.016 | 0.028 | 0.577 |
| rs2042555 | A | G | 0.406 | 0.014 | 0.002 | 9.45E-10 | -0.007 | 0.025 | 0.774 |
| rs2149351 | G | T | 0.767 | -0.016 | 0.003 | 2.27E-09 | 0.023 | 0.030 | 0.438 |
| rs28893517 | G | A | 0.071 | 0.028 | 0.005 | 5.16E-10 | 0.060 | 0.073 | 0.409 |
| rs297346 | G | A | 0.639 | -0.016 | 0.002 | 1.81E-11 | 0.009 | 0.026 | 0.729 |
| rs35738585 | G | T | 0.427 | -0.017 | 0.002 | 1.73E-13 | -0.040 | 0.025 | 0.107 |
| rs35755513 | T | C | 0.083 | 0.024 | 0.004 | 4.12E-08 | 0.059 | 0.058 | 0.312 |
| rs3843954 | C | G | 0.291 | 0.014 | 0.003 | 3.82E-08 | -0.013 | 0.027 | 0.619 |
| rs4578918 | C | T | 0.730 | -0.018 | 0.003 | 1.03E-11 | -0.078 | 0.028 | 0.004 |
| rs4632195 | T | C | 0.531 | 0.016 | 0.002 | 6.63E-12 | -0.007 | 0.024 | 0.791 |
| rs55965054 | T | C | 0.563 | -0.014 | 0.002 | 2.67E-09 | -0.019 | 0.025 | 0.444 |
| rs59382200 | G | A | 0.395 | -0.016 | 0.002 | 2.44E-11 | 0.050 | 0.026 | 0.048 |
| rs599550 | A | G | 0.860 | 0.027 | 0.003 | 3.69E-17 | -0.006 | 0.036 | 0.874 |
| rs60393230 | G | A | 0.612 | 0.014 | 0.002 | 3.57E-09 | 0.033 | 0.026 | 0.205 |
| rs62172117 | A | G | 0.351 | -0.014 | 0.002 | 6.19E-09 | 0.005 | 0.026 | 0.847 |
| rs6818081 | T | C | 0.151 | 0.018 | 0.003 | 2.83E-08 | -0.021 | 0.035 | 0.539 |
| rs6900114 | A | G | 0.271 | -0.015 | 0.003 | 2.30E-08 | 0.036 | 0.028 | 0.203 |
| rs7175083 | C | T | 0.508 | -0.015 | 0.002 | 2.21E-10 | 0.026 | 0.025 | 0.297 |
| rs75650221 | T | C | 0.040 | -0.036 | 0.006 | 3.08E-09 | -0.019 | 0.069 | 0.786 |
| rs76923064 | C | T | 0.019 | 0.056 | 0.010 | 3.24E-08 | 0.072 | 0.110 | 0.514 |
| rs77087420 | G | A | 0.050 | -0.033 | 0.005 | 1.06E-10 | 0.004 | 0.059 | 0.943 |
| rs7714426 | G | C | 0.839 | -0.018 | 0.003 | 1.41E-08 | 0.018 | 0.035 | 0.604 |
| rs77607745 | C | A | 0.187 | 0.016 | 0.003 | 4.95E-08 | -0.045 | 0.032 | 0.163 |
| rs7912226 | A | T | 0.397 | -0.015 | 0.002 | 8.12E-10 | -0.008 | 0.026 | 0.744 |
| rs836927 | A | C | 0.434 | 0.015 | 0.002 | 4.11E-10 | -0.003 | 0.025 | 0.902 |
| rs9852417 | A | C | 0.595 | -0.013 | 0.002 | 2.84E-08 | -0.026 | 0.025 | 0.307 |
| rs9858071 | C | T | 0.297 | 0.016 | 0.003 | 3.25E-10 | -0.015 | 0.027 | 0.576 |
| rs9930139 | C | A | 0.573 | 0.014 | 0.002 | 1.19E-08 | 0.010 | 0.025 | 0.689 |

| **Supplementary Table 44. MR results for the relationship between irritability and mediators** | | | | |
| --- | --- | --- | --- | --- |
|  | Number of SNPs | F-  statistic | OR (95% CI) | P-value |
| **Irritability on Smoking** | | | | |
| IVW | 21 | 37 | 1.919(1.276,2.886) | 0.002 |
| Weighted median |  |  | 1.602(1.049,2.447) | 0.029 |
| MR-Egger |  |  | 0.515(0.049,5.358) | 0.585 |
| MR-Egger intercept |  |  | - | 0.278 |
| Q statistic |  |  | - | <0.001 |
| **Irritability on Insomnia** | | | | |
| IVW | 21 | 37 | 4.324(2.359,7.929) | <0.001 |
| Weighted median |  |  | 4.462(1.837,10.839) | 0.001 |
| MR-Egger |  |  | 30.730(0.688,1372) | 0.093 |
| MR-Egger intercept |  |  | - | 0.317 |
| Q statistic |  |  | - | 0.26 |
| **Irritability on Depressed affect** | | | | |
| IVW | 20 | 37 | 3.327(2.682,4.127) | <0.001 |
| Weighted median |  |  | 3.312(2.548,4.304) | <0.001 |
| MR-Egger |  |  | 1.587(0.443,5.689) | 0.487 |
| MR-Egger intercept |  |  | - | 0.264 |
| Q statistic |  |  | - | 0.008 |

IVW, inverse variance weighted; SNP, single nucleotide polymorphism; OR, odds ratio; CI, confidence intervals; All statistical tests were two-sided. A P-value< 0.05 was considered significant.

| **Supplementary Table 45. MR results for the relationship between smoking and CVDs** | | | | |
| --- | --- | --- | --- | --- |
|  | Number of SNPs | F-statistic | OR (95% CI) | P-value |
| **Smoking on Coronary artery disease** | | | | |
| IVW | 74 | 41 | 1.218(1.104,1.343) | <0.001 |
| Weighted median |  |  | 1.253(1.123,1.398) | <0.001 |
| MR-Egger |  |  | 1.346(0.781,2.318) | 0.661 |
| MR-Egger ***intercept*** |  |  | - | 0.716 |
| Q statistic |  |  | - | <0.001 |
| **Smoking on Coronary angiopasty** | | | | |
| IVW | 73 | 41 | 1.443(1.192,1.748) | <0.001 |
| Weighted median |  |  | 1.503(1.134,1.993) | 0.005 |
| MR-Egger |  |  | 0.783(0.264,2.325) | 0.661 |
| MR-Egger ***intercept*** |  |  | - | 0.266 |
| Q statistic |  |  | - | 0.207 |
| **Smoking on Atrial fibrillation** | | | | |
| IVW | 73 | 41 | 1.126(0.970,1.307) | 0.119 |
| Weighted median |  |  | 1.150(0.923,1.432) | 0.214 |
| MR-Egger |  |  | 1.785(0.782,4.076) | 0.173 |
| MR-Egger ***intercept*** |  |  | - | 0.269 |
| Q statistic |  |  | - | 0.326 |
| **Smoking on Hypertensive heart disease** | | | | |
| IVW | 73 | 41 | 1.034(0.804,1.330) | 0.795 |
| Weighted median |  |  | 1.044(0.719,1.516) | 0.82 |
| MR-Egger |  |  | 0.696(0.174,2.782) | 0.61 |
| MR-Egger ***intercept*** |  |  | - | 0.571 |
| Q statistic |  |  | - | 0.355 |
| **Smoking on Non-ischemic cardiomyopathy** | | | | |
| IVW | 73 | 41 | 1.164(1.004,1.349) | 0.044 |
| Weighted median |  |  | 1.105(0.899,1.358) | 0.342 |
| MR-Egger |  |  | 1.572(0.700,3.529) | 0.277 |
| MR-Egger ***intercept*** |  |  | - | 0.461 |
| Q statistic |  |  | - | 0.391 |
| **Smoking on Heart failure** | | | | |
| IVW | 73 | 41 | 1.197(1.107,1.295) | <0.001 |
| Weighted median |  |  | 1.198(1.070,1.341) | 0.002 |
| MR-Egger |  |  | 1.154(0.707,1.883) | 0.569 |
| MR-Egger ***intercept*** |  |  | - | 0.88 |
| Q statistic |  |  | - | 0.071 |
| **Smoking on Ischemic stroke (large artery atherosclerosis)** | | | | |
| IVW | 74 | 41 | 1.302(1.015,1.672) | 0.038 |
| Weighted median |  |  | 1.358(0.947,1.946) | 0.096 |
| MR-Egger |  |  | 2.809(0.698,11.30) | 0.150 |
| MR-Egger ***intercept*** |  |  | - | 0.275 |
| Q statistic |  |  | - | 0.990 |

IVW, inverse variance weighted; SNP, single nucleotide polymorphism; OR, odds ratio; CI, confidence intervals; All statistical tests were two-sided. A P-value< 0.05 was considered significant.

| **Supplementary Table 46. MR results for the relationship between insomnia and CVDs** | | | | |
| --- | --- | --- | --- | --- |
|  | Number of SNPs | F-statistic | OR (95% CI) | P-value |
| **Insomnia on Coronary artery disease** | | | | |
| IVW | 22 | 25 | 1.082(1.015,1.152) | 0.015 |
| Weighted median |  |  | 1.074(0.979,1.179) | 0.132 |
| MR-Egger |  |  | 1.041(0.913,1.187) | 0.555 |
| MR-Egger ***intercept*** |  |  | - | 0.513 |
| Q statistic |  |  | - | 0.333 |
| **Insomnia on Coronary angiopasty** | | | | |
| IVW | 22 | 25 | 1.007(0.849,1.195) | 0.934 |
| Weighted median |  |  | 0.953(0.749,1.213) | 0.695 |
| MR-Egger |  |  | 0.976(0.693,1.376) | 0.893 |
| MR-Egger ***intercept*** |  |  | - | 0.839 |
| Q statistic |  |  | - | 0.564 |
| **Insomnia on Atrial fibrillation** | | | | |
| IVW | 22 | 25 | 1.011(0.854,1.198) | 0.898 |
| Weighted median |  |  | 0.950(0.774,1.164) | 0.619 |
| MR-Egger |  |  | 1.149(0.817,1.617) | 0.433 |
| MR-Egger ***intercept*** |  |  | - | 0.406 |
| Q statistic |  |  | - | 0.039 |
| **Insomnia on Hypertensive heart disease** | | | | |
| IVW | 22 | 25 | 1.004(0.802,1.255) | 0.975 |
| Weighted median |  |  | 0.344(0.965,0.660) | 0.194 |
| MR-Egger |  |  | 0.531(0.966,0.549) | 0.289 |
| MR-Egger ***intercept*** |  |  | - | 0.88 |
| Q statistic |  |  | - | 0.059 |
| **Insomnia on Non-ischemic cardiomyopathy** | | | | |
| IVW | 22 | 25 | 1.180(1.034,1.347) | 0.014 |
| Weighted median |  |  | 1.221(1.014,1.471) | 0.035 |
| MR-Egger |  |  | 1.340(1.028,1.748) | 0.043 |
| MR-Egger ***intercept*** |  |  | - | 0.29 |
| Q statistic |  |  | - | 0.443 |
| **Insomnia on Heart failure** | | | | |
| IVW | 22 | 25 | 1.038(0.966,1.116) | 0.310 |
| Weighted median |  |  | 1.032(0.924,1.152) | 0.577 |
| MR-Egger |  |  | 1.039(0.880,1.226) | 0.657 |
| MR-Egger ***intercept*** |  |  | - | 0.992 |
| Q statistic |  |  | - | 0.165 |
| **Insomnia on Ischemic stroke (large artery atherosclerosis)** | | | | |
| IVW | 23 | 25 | 1.125(0.801,1.580) | 0.496 |
| Weighted median |  |  | 1.086(0.727,1.623) | 0.687 |
| MR-Egger |  |  | 0.992(0.494,1.992) | 0.981 |
| MR-Egger ***intercept*** |  |  | - | 0.687 |
| Q statistic |  |  | - | 0.003 |

IVW, inverse variance weighted; SNP, single nucleotide polymorphism; OR, odds ratio; CI, confidence intervals; All statistical tests were two-sided. A P-value< 0.05 was considered significant.

| **Supplementary Table 47. MR results for the relationship between depressed affect and CVDs** | | | | |
| --- | --- | --- | --- | --- |
|  | Number of SNPs | F-statistic | OR (95% CI) | P-value |
| **Depressed affect on Coronary artery disease** | | | | |
| IVW | 38 | 38 | 1.279(1.094,1.495) | 0.002 |
| Weighted median |  |  | 1.316(1.052,1.645) | 0.016 |
| MR-Egger |  |  | 0.852(0.375,1.934) | 0.704 |
| MR-Egger ***intercept*** |  |  | - | 0.325 |
| Q statistic |  |  | - | 0.043 |
| **Depressed affect on Coronary angiopasty** | | | | |
| IVW | 34 | 38 | 1.480(0.939,2.333) | 0.091 |
| Weighted median |  |  | 1.341(0.706,2.549) | 0.370 |
| MR-Egger |  |  | 0.756(0.117,4.862) | 0.770 |
| MR-Egger ***intercept*** |  |  | - | 0.471 |
| Q statistic |  |  | - | 0.540 |
| **Depressed affect on Atrial fibrillation** | | | | |
| IVW | 34 | 38 | 1.352(0.877,2.084) | 0.173 |
| Weighted median |  |  | 1.144(0.652,2.006) | 0.640 |
| MR-Egger |  |  | 0.383(0.067,2.183) | 0.288 |
| MR-Egger ***intercept*** |  |  | - | 0.153 |
| Q statistic |  |  | - | 0.034 |
| **Depressed affect on Hypertensive heart disease** | | | | |
| IVW | 34 | 38 | 1.950(1.074,3.540) | 0.028 |
| Weighted median |  |  | 1.434(0.594,3.459) | 0.422 |
| MR-Egger |  |  | 0.795(0.059,10.731) | 0.864 |
| MR-Egger ***intercept*** |  |  | - | 0.491 |
| Q statistic |  |  | - | 0.275 |
| **Depressed affect on Non-ischemic cardiomyopathy** | | | | |
| IVW | 34 | 38 | 1.285(0.795,2.079) | 0.306 |
| Weighted median |  |  | 1.138(0.660,1.961) | 0.641 |
| MR-Egger |  |  | 0.245(0.036,1.651) | 0.158 |
| MR-Egger ***intercept*** |  |  | - | 0.089 |
| Q statistic |  |  | - | 0.002 |
| **Depressed affect on Heart failure** | | | | |
| IVW | 34 | 38 | 1.260(1.055,1.505) | 0.011 |
| Weighted median |  |  | 1.171(0.899,1.524) | 0.241 |
| MR-Egger |  |  | 1.543(0.622,3.831) | 0.356 |
| MR-Egger ***intercept*** |  |  | - | 0.656 |
| Q statistic |  |  | - | 0.079 |
| **Depressed affect on Ischemic stroke (large artery atherosclerosis)** | | | | |
| IVW | 38 | 38 | 1.047(0.595,1.843) | 0.873 |
| Weighted median |  |  | 1.084(0.477,2.462) | 0.847 |
| MR-Egger |  |  | 4.405(0.253,76.698) | 0.316 |
| MR-Egger ***intercept*** |  |  | - | 0.319 |
| Q statistic |  |  | - | 0.172 |

IVW, inverse variance weighted; SNP, single nucleotide polymorphism; OR, odds ratio; CI, confidence intervals; All statistical tests were two-sided. A P-value< 0.05 was considered significant.


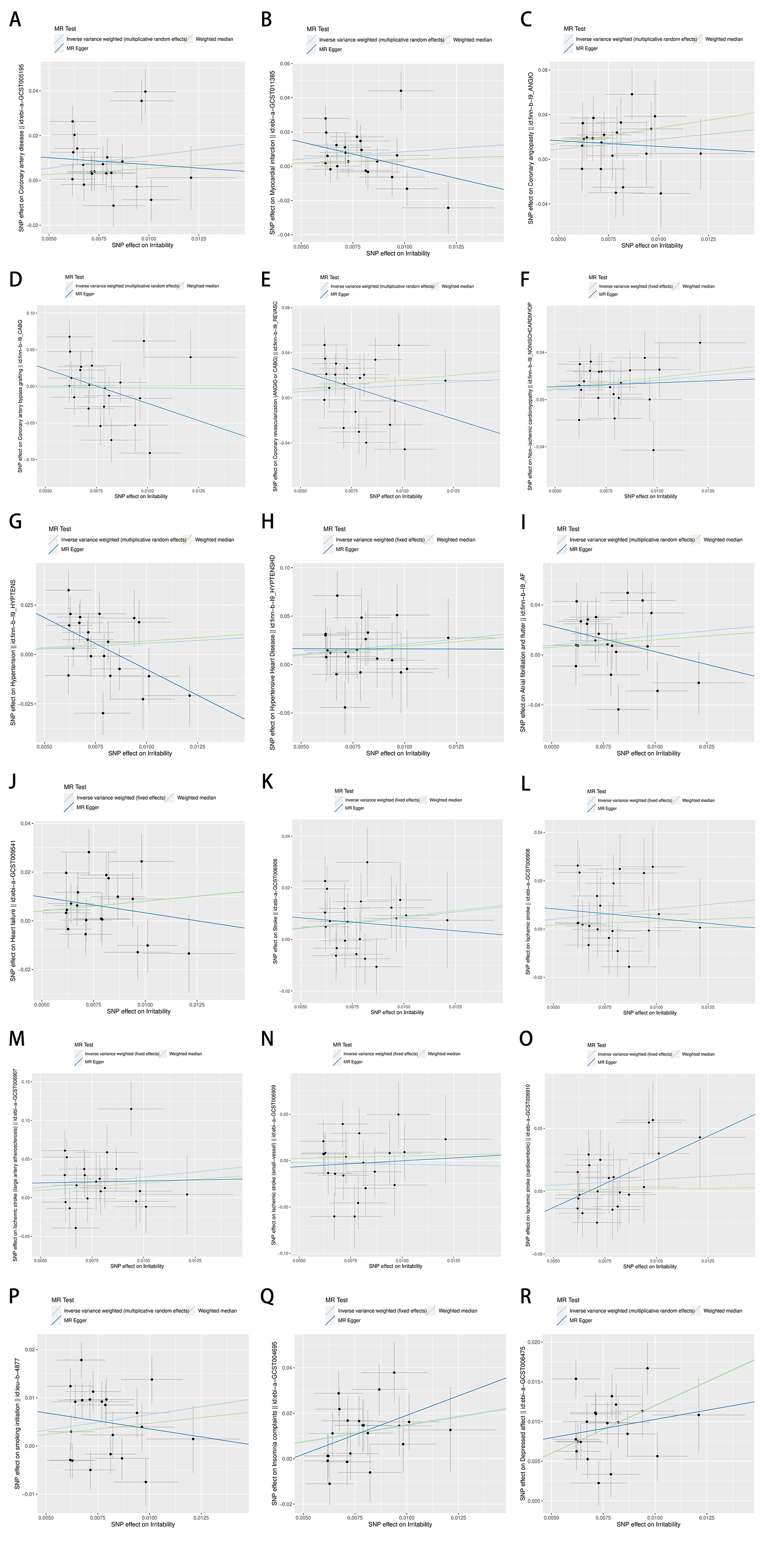


Supplementary Figure 1. Scatter plot using all IVs of irritability on CVDs. (A) irritability and CAD; (B) irritability and MI; (C) irritability and CA; (D) irritability and CABG; (E) irritability and RE; (F) irritability and NIC; (G) irritability and HTN; (H) irritability and HHD; (I) irritability and AF; (J) irritability and HF; (K) irritability and stroke; (L) irritability and IS; (M) irritability and ISla; (N) irritability and ISsv; (O) irritability and ISce; (P) irritability and smoking; (Q) irritability and insomnia; (R) irritability and depressed affect.


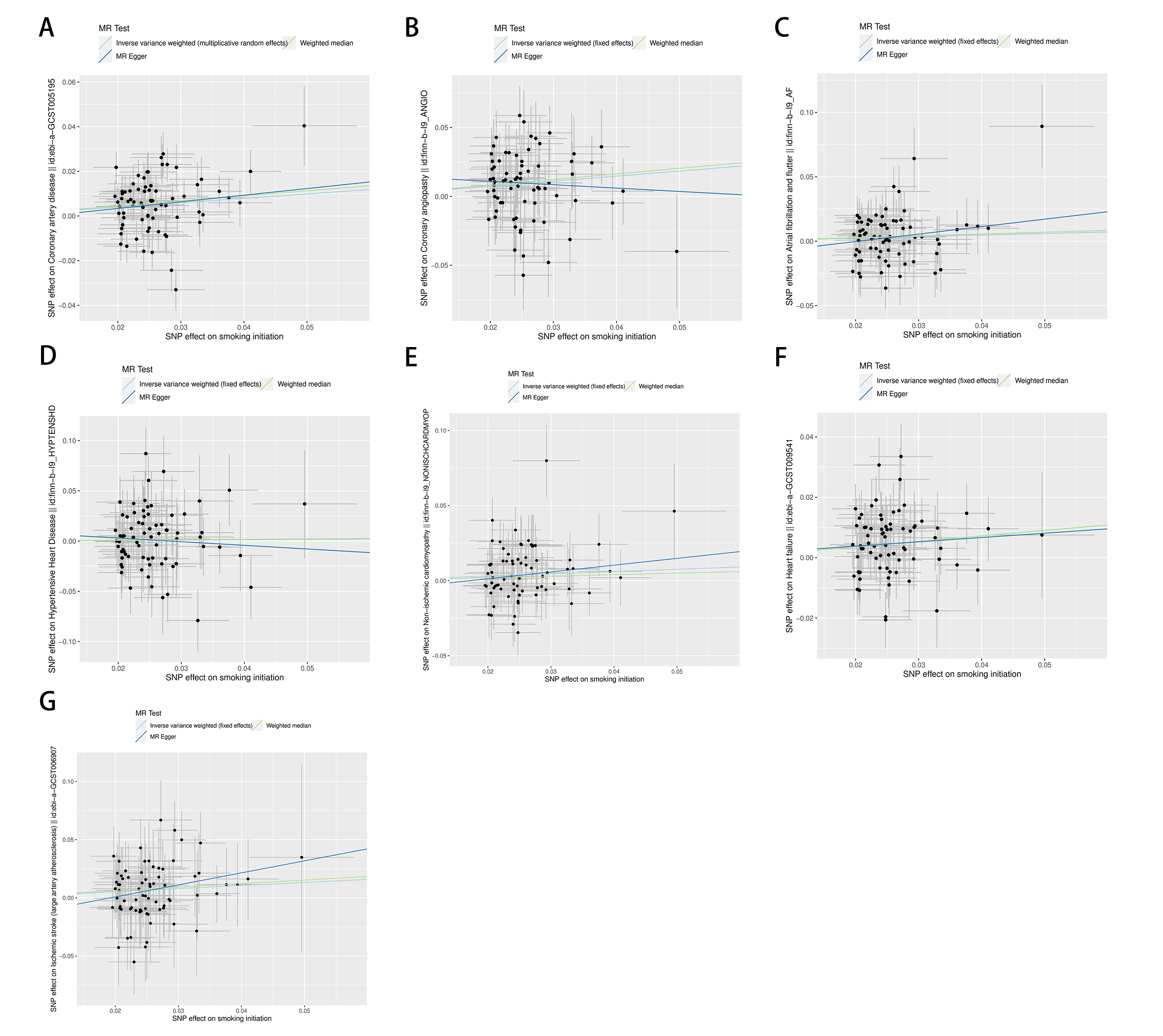


Supplementary Figure 2. Scatter plot using all IVs of smoking on CVDs. (A) smoking and CAD; (B) smoking and CA; (C) smoking and AF; (D) smoking and HHD; (E) smoking and NIC; (F) smoking and HF; (G) smoking and ISla.


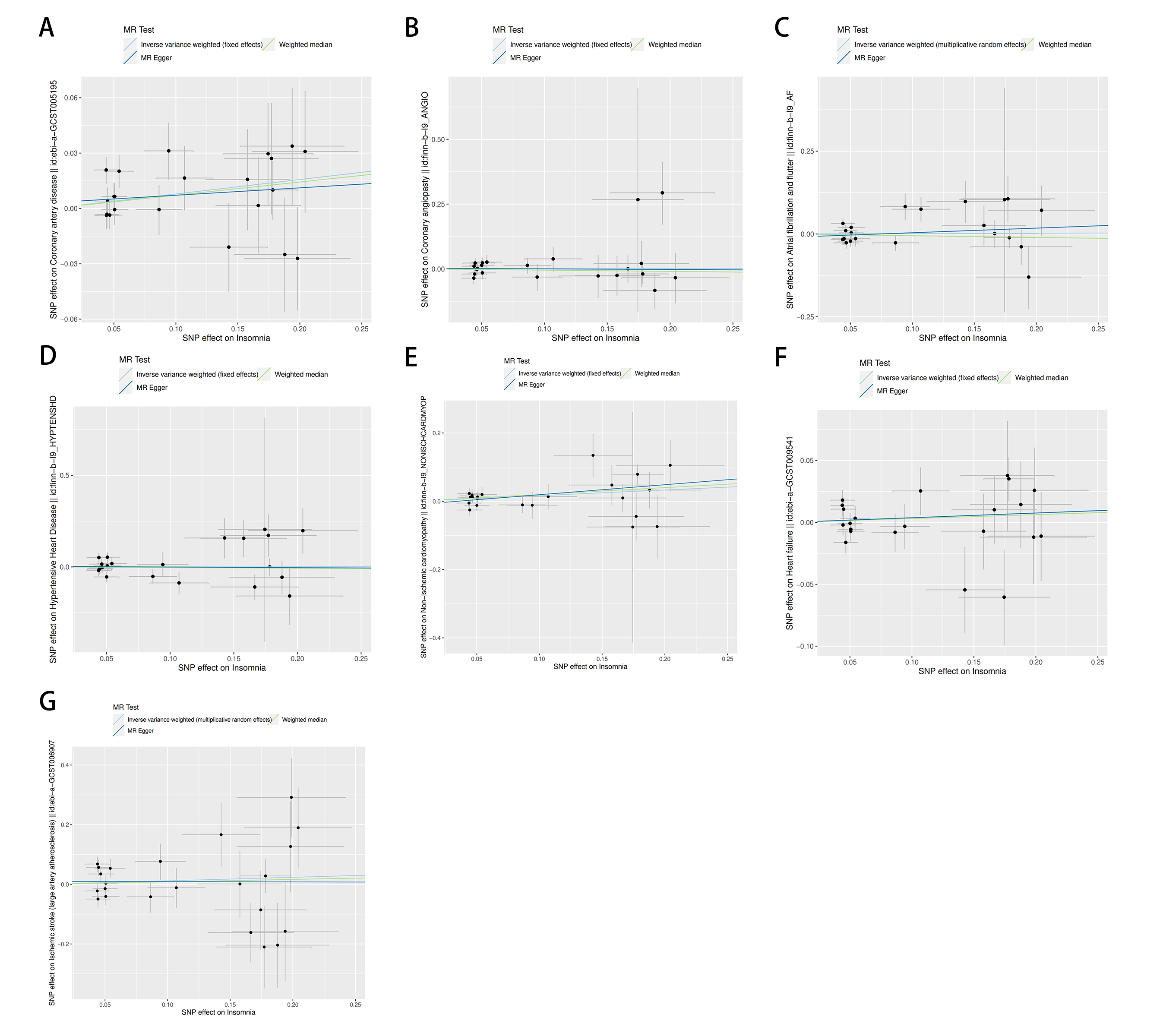


Supplementary Figure 3. Scatter plot using all IVs of insomnia on CVDs. (A) insomnia and CAD; (B) insomnia and CA; (C) insomnia and AF; (D) insomnia and HHD; (E) insomnia and NIC; (F) insomnia and HF; (G) insomnia and ISla.


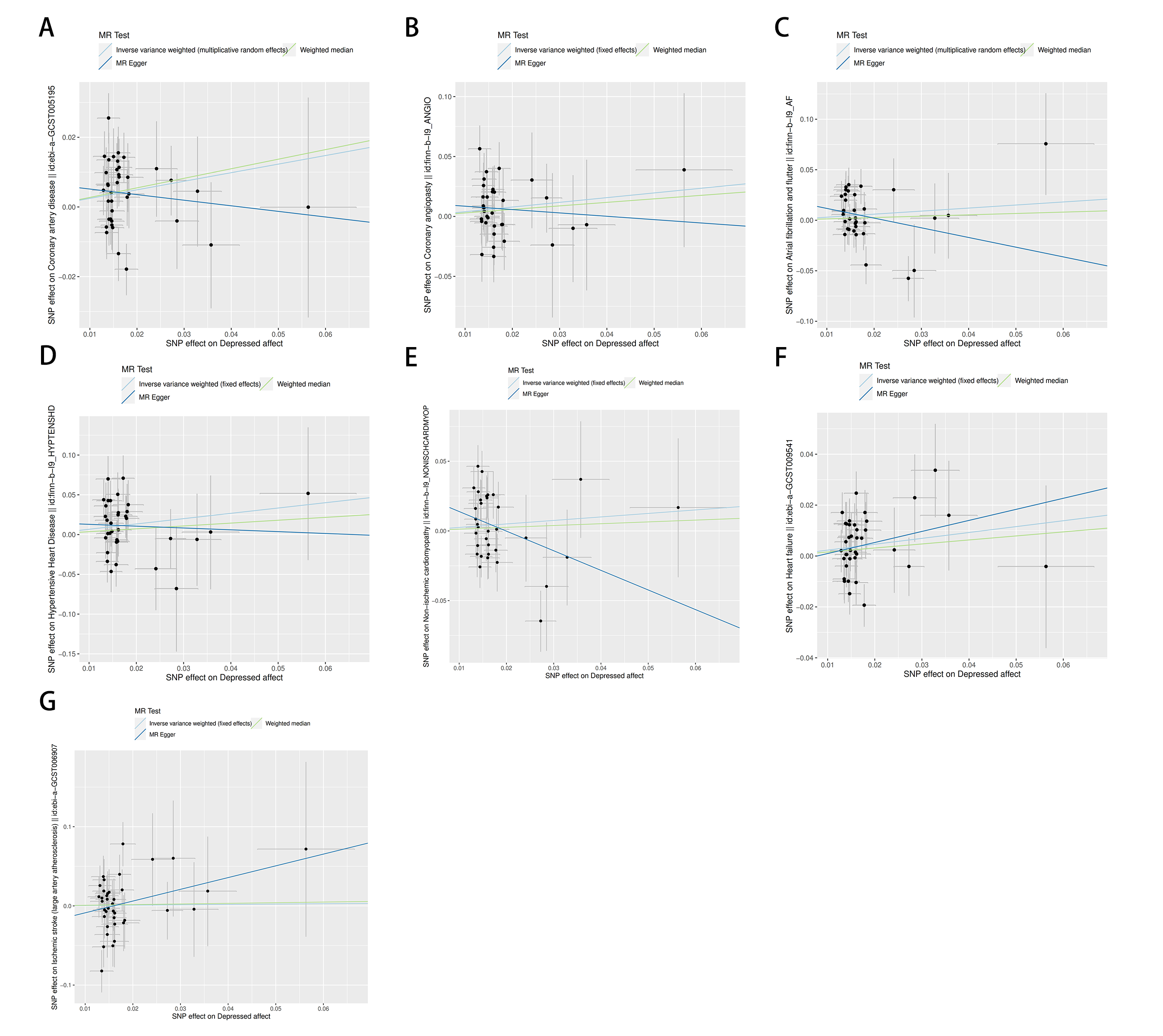


Supplementary Figure 4. Scatter plot using all IVs of depressed affect on CVDs. (A) depressed affect and CAD; (B) depressed affect and CA; (C) depressed affect and AF; (D) depressed affect and HHD; (E) depressed affect and NIC; (F) depressed affect and HF; (G) depressed affect and ISla.
